# Inter-comparison of measurements of inorganic chemical components in precipitation from NADP and CAPMoN at collocated sites in the USA and Canada during 1986–2019

**DOI:** 10.1007/s10661-023-11771-z

**Published:** 2023-10-18

**Authors:** Jian Feng, Amanda Cole, Gregory A. Wetherbee, Kulbir Banwait

**Affiliations:** 1 Air Quality Measurement and Analysis Research Section, Atmospheric Science and Technology Directorate, Environment and Climate Change, 4905 Dufferin Street, Toronto, ON M3H 5T4 Canada; 2 U.S. Geological Survey, Water Mission Area - Observing Systems Division, Denver Federal Center, Mail Stop 401, Bldg. 95, Box 25046, Denver, CO 80225 USA

**Keywords:** Canadian Air and Precipitation Monitoring Network, National Atmospheric Deposition Program, Wet deposition

## Abstract

Wet deposition monitoring is a critical part of the long-term monitoring of acid deposition, which aims to assess the ecological impact of anthropogenic emissions of SO_2_ and NO_x_. In North America, long-term wet deposition has been monitored through two national networks: the Canadian Air and Precipitation Monitoring Network (CAPMoN) and the US National Atmospheric Deposition Program (NADP), for Canada and the USA, respectively. In order to assess the comparability of measurements from the two networks, collocated measurements have been made at two sites, one in each country, since 1986 (Sirois et al., in *Environmental Monitoring and Assessment, 62*, 273–303, 2000; Wetherbee et al., in *Environmental Monitoring and Assessment*, 1995–2004, 2010). In this study, we compared the measurements from NADP and CAPMoN instrumentation at the collocated sites at the Pennsylvania State University (Penn State), USA, from 1989 to 2016, and Frelighsburg, Quebec, Canada, from 2002 to 2019. We also included in the study the collocated daily-vs-weekly measurements by the CAPMoN network during 1999–2001 and 2016–2017 in order to evaluate the differences in wet concentration of ions due to sampling frequency alone. The study serves as an extension to two previous CAPMoN-NADP inter-comparisons by Sirois et al. (*Environmental Monitoring and Assessment, 62*, 273–303, 2000) and Wetherbee et al., in (*Environmental Monitoring and Assessment*, 1995–2004, 2010). At the Penn State University site, for 1986–2019, CAPMoN was higher than NADP for all ions, in terms of weekly concentration, precipitation-weighted annual mean concentration, and annual wet deposition. The precipitation-weighted annual mean concentrations were higher for SO_4_^2−^ (2%), NO_3_^−^ (12%), NH_4_^+^ (16%), H^+^ (6%), and base cations and Cl^−^ (11–15%). For annual wet deposition, CAPMoN was higher for SO_4_^−2^, NO_3_^−^, NH_4_^+^ and H^+^ (5–17%), and base cations and Cl^−^ (12–17%) during 1986–2019. At the Frelighsburg site, NADP changed the sample collector in October 2011. For 2002–2011, the relative differences at the Frelighsburg site were positive and similar in magnitude to those at the Penn State site. For 2012–2019, the precipitation-weighted annual mean concentrations were 5–27% lower than NADP, except for H^+^, which was 23% higher. The change in sample collector by NADP had the largest effect on between-network biases. The comparisons of daily-vs-weekly measurements conducted by the CAPMoN network during 1999–2001 and 2016–2017 show that the weekly measurements were higher than the daily measurements by 1–3% for SO_4_^2−^, NO_3_^−^, and NH_4_^+^; 3–9% for Ca^2+^, Mg^2+^, Na^+^, and Cl^−^; 10–24% for K^+^; and lower for H^+^ by 8–30% in terms of precipitation-weighted mean concentration. Thus, differences in sampling frequencies did not contribute to the systematically higher CAPMoN measurements. Understanding the biases in the data for these networks is important for interpretation of continental scale deposition models and transboundary comparison of wet deposition trends.

**Supplementary Information:**

The online version contains supplementary material available at 10.1007/s10661-023-11771-z.

## Introduction

Anthropogenic emissions of SO_2_ and NO_x_ (NO_x_ = NO + NO_2_) have significant impact on ecological systems. Long-term and routine measurements of chemical components of precipitation are critical parts of the long-term monitoring of acid deposition, and have been carried out around the world, e.g., the European Monitoring and Evaluation Programme (EMEP) and the Acid Deposition Monitoring Network in East Asia (EANET) (Duan et al., 2016; Torseth et al., 2012; Vet et al., 2014; Yagishita, 1995). In North America, long-term wet deposition monitoring is carried out through two national networks: the Canadian Air and Precipitation Monitoring Network (CAPMoN) and the United States National Atmospheric Deposition Program (NADP), for Canada and the USA, respectively (Blanchard et al., 1996; Feng et al., 2021; Likens et al., 2021; Lynch et al., 1995; Vet et al., 2014).

The measurements of wet deposition by NADP go back as far as 1978 through the establishment of the National Trend Network (NTN). The NTN is the only network providing long-term measurements of chemical components in precipitation across the USA (https://nadp.slh.wisc.edu/networks/national-trends-network). NADP also operated the Atmospheric Integrated Research Monitoring Network (AIRMoN) during 1992–2019. Samples from the NTN are collected on a weekly basis, while samples from the AIRMoN were collected on a 24-h basis, starting from the onset of a precipitation event. The NADP samples are sent to the Central Analytical Laboratory (CAL), which was within the University of Illinois prior to 2018 and currently is at the Wisconsin State Laboratory of Hygiene (WSLH).

The CAPMoN network has been operated by Environment and Climate Change Canada (ECCC) since 1983 (Sirois et al., 2000). CAPMoN measures pollutants in both the air through integrated air filter pack sampling and in precipitation through bulk precipitation sampling at rural and remote sites across Canada. Precipitation samples are collected on a 24-h basis, within 1 h of 8:00 AM local time each day. Starting in 2017, weekly precipitation sampling began at a subset of CAPMoN sites. As of February 2023, wet deposition samples are collected at 24 sites, 7 of which are operating on a weekly basis. All samples are analyzed at ECCC’s laboratory in Toronto, Ontario, Canada.

The measurement data from CAPMoN and NADP have been extensively used in over 100 studies annually (David Gay, NADP Coordinator, personal communication, 2022; Feng et al., 2021; Cheng et al., 2022), some of which included data from both networks. In addition, these data are combined to jointly report on the effectiveness of the Acid Rain Annex of the binational Air Quality Agreement (e.g., Canada – United States Air Quality Agreement Progress Report, 2018, https://publications.gc.ca/site/eng/9.506241/publication.html). In order to assess the comparability of data from the two networks, collocated CAPMoN and NADP measurements have been made since 1986 at two sites, one in each country. The measurements were analyzed by Sirois et al. (2000) and by Wetherbee et al. (2010) to quantify between-network biases up to 2004.

Sirois et al. (2000) found statistically significant biases for weekly concentrations of all ions except for SO_4_^2−^ for 1986–1993. Overall, the wet deposition estimated with NADP measurements was 7–35% lower than with CAPMoN measurements. Wetherbee et al. (2010) found the same to slightly lower between-network biases for most ions except for SO_4_^2−^ and NO_3_^−^. The annual deposition estimated with NADP measurements was 16–31% lower than that estimated with CAPMoN measurements for 1995–2004 (Wetherbee et al., 2010). Both Sirois et al. (2000) and Wetherbee et al. (2010) discussed potential causes for between-network biases, namely that the measured concentrations from CAPMoN were systematically higher than those from NADP. The potential factors contributing to the observed between-network biases include the lids of CAPMoN’s collectors opening earlier than the lids of NADP’s collectors, difference in sampling frequency (i.e., daily vs. weekly), analytical laboratory bias, background contamination and sample contamination, sample filtering by NADP vs. no sample filtering by CAPMoN. However, no decisive conclusion was reached for the causes of the between-network biases in Sirois et al. (2000) and Wetherbee et al. (2010).

For the current study, besides more collocated data measured for 2005–2019, we also include collocated daily-vs-weekly measurements carried out by the CAPMoN network during 1999–2001 and 2016–2017. The current study is an extension to the two previous studies by Sirois et al. (2000) and Wetherbee et al. (2010), for continued evaluation of the comparability of measurements from the two networks. Additionally, this study investigates the change of between-network biases over 1986–2019 and causes of the between-network biases.

## Experimental design and measurement protocols

### Experimental design

Experimental design and measurement protocols have been described in detail in Sirois et al. (2000) and Wetherbee et al. (2010) under the same title. Here, we describe briefly the experimental design for completeness and update some measurement protocols that have evolved since the two previous reports.

CAPMoN and NADP have operated collocated measurements since 1986 (Sirois et al., 2000; Wetherbee et al., 2010), one site within each country. In the USA, the collocated site is located at Pennsylvania State University, Center County, PA (thereafter, the site is referred to as the Penn State site, coded as PA15 for NADP, and PEN for CAPMoN). The site is about 6 km from the town of State College in rural Pennsylvania and is the middle of a broad valley between two ridges of the Appalachian Mountains, as described in Sirois et al. (2000). The site is an approximate 180 m by 180 m square covered with mown grass within a forested area, with latitude, longitude, and altitude of 40° 47′ 18″ N, 77*°* 56′ 48″ W, and 393 m above sea level. In Canada, the collocated measurement site was situated at Sutton, Quebec, from 1986 to 2001. The Sutton site (coded as CAN4 by NADP, and SUT by CAPMoN) was in a rural area of southwestern Quebec, 5 km from the Canada-US border (45° 4′ 35″ N, 72° 40′ 35″ W, and 243 m above sea level). The instruments were situated on a grassy open field surrounded by mixed forest. In late 2001, the Sutton site was relocated by 15 km to Frelighsburg, Quebec (coded as CAN5 by NADP, and FRE by CAPMoN, 45° 3′ 6″ N, 72° 51′ 42″ W, and 203 m above sea level). The Frelighsburg site is in a rural area with some agricultural land use, well away from influence of industrial and urban activities, as described by Wetherbee et al. (2010). In early 2016, the Frelighsburg site was moved 1 km south to 45° 02′ 28″ N, 72° 51′ 29″ W, with altitude 235 m above sea level.

### Measurement protocols

CAPMoN and NADP samples are analyzed for nine major ions: sulfate (SO_4_^2−^), nitrate (NO_3_^−^), ammonium (NH_4_^+^), hydrogen ion (H^+^ from pH), calcium (Ca^2+^), magnesium (Mg^2+^), sodium (Na^+^), potassium (K^+^), and chloride (Cl^−^). Specific conductance is also measured by NADP. CAPMoN also analyzes samples for total nitrogen.

CAPMoN precipitation samples were collected using the wet-only MIC C300 precipitation collectors manufactured by Meteorological Instruments of Canada (Wetherbee et al., 2010) at the Penn State site for the entire study period, and at Frelighsburg up to January 2019. The Xancon D400 samplers replaced the C300 samplers in the network in 2019 and provide near real-time monitoring, onboard data storage, data-logger connectivity, and improved precipitation sensors. The collection bucket opening of each CAPMoN sampler is 1.5 ± 0.1 m above the ground. Samples are collected on a 24-h basis within 1 h of 8:00 AM local standard time. Precipitation depth is measured with the Meteorological Service of Canada (MSC) Type-B rain gauge for liquid and freezing precipitation. The rain gauge has a capacity to capture over 250 mm of rainfall. A MSC Nipher-shielded snow gauge (Wetherbee et al., 2010) is used to measure precipitation depth of snowfall. Sample depth is calculated from sample volume, and used only when precipitation depth from the gauges is not available or considered to be unreliable. Each sample is collected in a sample collection bucket lined with a plastic bag. The bag contains a thin virgin polyethylene inner layer, thus reducing sample contamination and sample absorption. The sample bag is changed daily regardless of occurrence or absence of precipitation within the 24-h sampling period. No sample preservation, sample transfer (except sample amounts greater than 2 L are transferred to a second sample bag and sealed to avoid sample bag rupture and leaks during transport), or parameter measurements (e.g., pH or conductivity) are conducted in the field. All sampled bags, whether they represent non-event dry bags, wet deposition, or bulk (wet plus dry) deposition, are removed from the sampling buckets and heat-sealed, weighed, and immediately stored at 4 °C until being shipped in icepack-cooled containers to the CAPMoN analytical laboratory in Toronto, Ontario. Shipments from the field occur once every 2 weeks (Sirois et al., 2000). A full description of CAPMoN precipitation operational procedures is available at http://publications.gc.ca/site/fra/9.883036/publication.html. Although most of the electronic equipment and some analytical methods have changed since the stated reference, the non-electronic equipment, collection, observation, and operational methods remain the same in principle.

The analytical laboratory receives and logs the samples, examining them for leaks, noting weight and other attributes. Samples are kept at 4 °C until analysis, normally within 1 week. Samples are transferred from sample bags to pre-cleaned 4 oz polyethylene bottles for easy handling between different analytical stations. The remaining portions of samples are discarded. Ion chromatography (IC) is utilized for Cl^−^, SO_4_^2−^, and NO_3_^−^ . Na^+^ and K^+^ were also analyzed by IC until 2017, and thereafter by inductively coupled plasma atomic emission spectroscopy (ICP-AES). Flame atomic absorption spectrometry was used for Ca^2+^and Mg^2+^ until 2013, when they began to be analyzed by ICP-AES. Flow injection analysis (FIA) colorimetry is utilized for total nitrogen (TN) and NH_4_^+^, and electrometry for pH. Trace samples (<= 0.2 mm of precipitation depth) are not analyzed. Sample analysis is prioritized for analysis of anions first, followed by base cations, NH_4_^+^, pH, and TN. Non-trace samples with minimum pipette volume of 0.5 mL or 1 mL were diluted to 9 or 5 times dilution, respectively, enough for anion analysis only. Samples with enough volume for complete analytical analysis are also diluted when the samples look highly contaminated prior to analysis or when they are determined to be outside the calibration range. pH analysis is not performed on diluted samples. Data quality assurance and quality control are performed using the Research Data Management and Quality Assurance (RDMQ) system (McMillan et al., 2000) developed by Environment and Climate Change Canada (ECCC).

Prior to 2018, weekly composited NADP samples were collected in deionized water (DI)–cleaned, high-density polyethylene (HDPE) buckets and transferred to DI-cleaned, 1-L HDPE bottles for shipment to the NADP Central Analytical Laboratory (CAL) at the University of Illinois-Urbana/Champaign (UI). In 2018, the NADP and CAL moved from UI to the Wisconsin State Laboratory of Hygiene, University of Wisconsin-Madison (UW). The NADP transitioned to a bag-lined bucket sampling system in 2020, the data for which are from beyond the study period. N-CON Systems, Inc. Model ADS 00-120 (NCON) sample collectors replaced the Aerochem Metrics Model 301 (ACM) collectors in a subset of NADP sites since 2010 (Wetherbee, 2017).

Samples were shipped unpreserved and not refrigerated, but as soon after collection as possible (1–2 days). Prior to 2016, samples were analyzed for pH and specific conductance upon receipt, then filtered for the remaining analyses. During the study period, anions were measured by IC, major cations by inductively coupled argon plasma optical emission spectrometry (ICPOES), ammonium by FIA, and electrometry for pH and specific conductance. At the start of the study period, pH and specific conductance were measured first, and then samples of trace quantity were diluted to suitable volumes for remaining analyses. This analytical priority changed in 2016, and the order became FIA > IC > ICPOES, and then pH > specific conductance if undiluted sample remained. In 2017, the CAL transitioned to a new IC method that uses a separation column and eluent that are both different from those used by the previous method.

Methods of analysis and other quality assurance information for the NADP sample data are available from the NADP web site (https://nadp.slh.wisc.edu/networks/national-trends-network/) or by request. The NADP data are internally quality assured (NADP, 2019), and data collection systems are independently quality assured by the U.S. Geological Survey (USGS; Wetherbee & Martin, 2020).

## Data and analysis methods

### Data

Weekly precipitation-weighted concentration from CAPMoN daily samples is calculated using the following equation, following Sirois et al. (2000) and Wetherbee et al. (2010):

1 $${C}^{WM}=\left(\frac{\sum_{i=1}^n{C}_i{P}_i}{\sum_{i=1}^n{P}_i}\right)$$

where *n* is the number of valid daily measurements within a weekly interval, and *C*_*i*_ and *P*_*i*_ are daily concentration and daily precipitation amount from precipitation gauge for a given day. The units for *C*_*i*_ and *P*_*i*_ are mg L^−1^ and mm, respectively.

Seasonal or annual deposition is calculated as:

2 $${Deposition}_{seasonal/ annual}\left(\textrm{kg}/\textrm{ha}\right)=\left({\sum}_{j=1}^m{C}_j{P}_j\right)/100,$$

and seasonal and annual precipitation-weighted mean concentration is calculated as:

3 $${C}^{WM}=\left(\frac{\sum_{j=1}^m{C}_j{P}_j}{\sum_{j=1}^m{P}_j}\right),$$

where *C*_*j*_ and *P*_*j*_ are weekly concentration and weekly precipitation amount, in unit of mg L^−1^ and mm, respectively; *m* is the number of weeks within a year or a season. When *m* is the number of weeks within a study period, *C*^*WM*^ is the precipitation-weighted mean concentration for that study period. Precipitation-weighted seasonal and annual mean concentrations as well as seasonal and annual deposition were not calculated for the Frelighsburg site for 2001 due to limited measurement data available for that year. As mentioned in Wetherbee et al. (2010), CAPMoN and NADP data are censored to make the inter-comparison free from influence of missing data; therefore, the calculated annual or seasonal deposition underestimates the true value of deposition and should not be interpreted as a final estimation of seasonal or annual deposition.

### Analysis methods

A number of statistical metrics are calculated to assess the differences between CAPMoN and NADP measurements, mainly following Sirois et al. (2000) and Wetherbee et al. (2010). The metrics include arithmetic mean difference, median difference, relative differences in arithmetic mean and median, 1% Winsorized standard deviation and relative 1% Winsorized standard deviation, 90th percentile of difference, Pearson correlation coefficient, modified median absolute deviation (MMAD), and coefficient of variation (CoV). Two statistical tests, *t*-test and Wilcoxon signed-rank test, are applied to detect the statistical significance of the between-network biases. Most statistical metrics have been detailed in Sirois et al. (2000) and Wetherbee et al. (2010), and are briefly described as follows for completeness:

- The relative difference in arithmetic mean is defined as:

4 $$\frac{Mean\left({C}_{CAPMoN}-{C}_{NADP}\right)}{Mean\left({C}_{CAPMoN}\right)+ Mean\left({C}_{NADP}\right)}\times 2\times 100,$$

where:

*C*_*CAPMoN*_weekly precipitation-weighted mean CAPMoN concentration and

*C*_*NADP*_weekly NADP concentration.

In the above calculation, first we calculate the mean value of the differences of paired CAPMoN-NADP weekly concentrations, then we normalize this mean value with the average of the mean weekly concentrations of CAPMoN and NADP.

- Similarly, the relative difference in median is defined as:

5 $$\frac{Median\left({C}_{CAPMoN}-{C}_{NADP}\right)}{Median\left({C}_{CAPMoN}\right)+ Median\left({C}_{NADP}\right)}\times 2\times 100.$$

- Winsorized standard deviation (Barnett & Lewis, 1984) is a more robust estimation of variation that is determined by replacing values less than 1st percentile or greater than 99^th^ percentile with those percentiles, respectively, and then calculating the standard deviation.
  The relative Winsorized standard deviation is computed as:

6 $$\frac{Std\left({C}_{CAPMoN}-{C}_{NADP}\right)}{Median\left({C}_{CAPMoN}\right)+ Median\left({C}_{NADP}\right)}\times 2\times 100,$$

where *Std*(*C*_*CAPMoN*_ − *C*_*NADP*_) is the calculated Winsorized standard deviation.

- Modified median absolute deviation (MMAD) (Randles and Wolf, 1979) is a robust, non-parametric alternative to the traditional standard deviation, and is defined as:

7 $$\begin{aligned}MMAD=&\ \left(\frac{1}{0.6745}\right)\times median\left[\left|\left({C}_{CAPMoN}\right.\right.\right.\\&-\left.\left.\left.{C}_{NADP}\right)- median\left({C}_{CAPMoN}-{C}_{NADP}\right)\right|\right].\end{aligned}$$

In computing MMAD, first the median of a list of values, which in this study is the difference of each paired CAPMoN-NADP weekly concentration, is found; secondly, this median is subtracted from each value of the list; finally, a new median is determined from the new list. A coefficient 1/0.6745 is applied to the new median so that the calculated MMAD is the same as the traditional standard deviation when the list of value follows a normal distribution.

- CoV is calculated by normalizing MMAD with the average of CAPMoN and NADP medians, similar to the calculation of relative median and relative Winsorized standard deviation. It is a non-parametric coefficient of variation, and a good indicator for measuring the spread of a one-to-one scatter plot.
- *t*-test is commonly used to detect if the difference from two measurements is statistically significant. Implicitly, a *t*-test assumes that the difference from two measurements follows a normal distribution (Kim & Park, 2019). For the current study, differences between CAPMoN and NADP do not necessarily follow normal distributions (Sirois et al., 2000), especially for weekly concentrations. This is because the differences of weekly concentrations from the two measurements are sensitive to trace precipitation amount. Wilcoxon signed-rank test, which is a non-parametric test and does not require a normal distribution for the variable (Woolson, 2008), is also applied in the study. *t*-test and Wilcoxon signed-rank test are used to test whether the biases in mean or median are significantly (α=0.05) different from zero in statistics, respectively.

## Results

### Inter-network comparability of weekly data

In comparing weekly concentration, both arithmetic mean and median value are calculated and presented in Tables 1, 2, 3, and 4 and Table S.2. However, because high concentrations of ions are usually associated with low precipitation amounts, and the distribution of concentration vs. sample depth is not in Gaussian distribution (Sirois et al., 2000), the arithmetic mean is biased by those samples with low precipitation amount and is always higher than the median value in this inter-comparison study. Therefore, in discussion of inter-network comparability of weekly data, the relative difference of median value is used. As mentioned in Wetherbee et al. (2010), bias typically means the difference from the true or accepted value. In this study, we use bias to refer to the difference of CAPMoN-minus-NADP, and does not imply that either CAPMoN or NADP has more accurate measurements. In calculation of biases or relative biases, we use CAPMoN values minus NADP values through the study. Therefore, a positive bias indicates that CAPMoN has higher value than NADP. The cold and warm seasons are defined as November–April and May–October, respectively, in this study.

**Table 1:** Statistics for inter-comparisons of weekly ion concentrations and precipitation (Ppt) depths measured from co-located National Atmospheric Deposition Program (NADP) and Canadian Air and Precipitation Monitoring Network CAPMoN) sites at Pennsylvania State University, PA, USA, during period 1986–2019 [units are in milligrams per liter (mg L^−1^), percent (%), and millimeters (mm), or unitless (R, *p*-value), as indicated. *Diff.*, difference; *SD*, standard deviation from mean; *RSD*, percent standard deviation relative to mean of NADP and CAPMoN median values; *MMAD*, modified median absolute deviation between NADP and CAPMoN values; *CoV*, non-parametric coefficient of variation; *P90*, 90^th^ percentile value; Pearson’s *r*, coefficient for correlation of NADP and CAPMoN values; *p*-value, the probability of a null hypothesis is true. The null hypothesis here is that there is no difference between the means (or medians) of CAPMoN and NADP weekly concentrations. *p*-values shown are for Wilcoxon’s signed-rank test; *SO*_*4*_^*2*−^, sulfate; *NO*_*3*_^*−*^, nitrate; *NH*_*4*_^*+*^, ammonium; *H*^*+*^, hydrogen ion; *Ca*^*2+*^, calcium; *Cl*^*−*^, chloride; *K*^*+*^, potassium; *Mg*^*2+*^, magnesium; *Na*^*+*^, sodium. Data obtained from NADP, Wisconsin State Laboratory of Hygiene at https://nadp.slh.wisc.edu/networks/national-trends-network and Environment and Climate Change Canada at https://www.canada.ca/en/environment-climate-change/services/air-pollution/monitoring-networks-data/canadian-air-precipitation.html, last accessed August 2023]

	Mean of NADP	Mean of CAPMoN	Diff. of mean	Relative diff. of mean	Median of NADP	Median of CAPMoN	Diff. of median	Relative diff. of median	SD	RSD	MMAD	CoV	P90	Pearson’s *r*	*p*-value
Ion	mg L^−1^	mg L^−1^	mg L^−1^	%	mg L^−1^	mg L^−1^	mg L^−1^	%	mg L^−1^	%	mg L^−1^	%	mg L^−1^		
SO_4_^2−^	2.205	2.336	0.131	5.8	1.698	1.787	0.067	3.7	0.418	24.0	0.166	9.5	0.565	0.93	<0.001
NO_3_^−−^	1.847	2.128	0.281	14.1	1.450	1.687	0.148	8.8	0.447	28.5	0.184	11.7	0.747	0.93	<0.001
NH_4_^+^	0.333	0.401	0.069	18.7	0.258	0.325	0.052	16.0	0.102	35.1	0.053	18.3	0.177	0.83	<0.001
H^+^	0.045	0.052	0.007	13.8	0.036	0.040	0.003	7.6	0.013	34.0	0.007	17.1	0.021	0.95	<0.001
Ca^2+^	0.162	0.188	0.026	14.7	0.100	0.118	0.012	10.2	0.081	73.9	0.027	24.5	0.097	0.85	<0.001
Cl^−^	0.163	0.190	0.027	15.3	0.120	0.136	0.013	9.6	0.060	46.6	0.025	19.7	0.094	0.91	<0.001
K^+^	0.024	0.028	0.004	14.4	0.014	0.019	0.004	21.1	0.024	145.9	0.007	44.9	0.023	0.43	<0.001
Mg^2+^	0.025	0.030	0.005	18.0	0.016	0.019	0.003	15.8	0.013	76.7	0.004	25.4	0.016	0.84	<0.001
Na^+^	0.063	0.067	0.004	6.5	0.036	0.036	0.002	5.6	0.036	99.6	0.012	32.9	0.035	0.84	<0.001

Depth	mm	mm	mm	%	mm	mm	mm	%	mm	%	mm	%	mm		
Ppt	23.499	24.344	0.846	3.5	18.415	18.900	0.452	2.4	2.408	12.9	1.293	6.9	3.020	0.96	<0.001

**Table 2:** Statistics for inter-comparisons of weekly ion concentrations and precipitation (Ppt) depths measured from co-located National Atmospheric Deposition Program (NADP) and Canadian Air and Precipitation Monitoring Network CAPMoN) sites at Pennsylvania State University, PA, USA, during period 2005–2019 [units are in milligrams per liter (mg L^−1^), percent (%), and millimeters (mm), or unitless (R, *p*-value), as indicated. *Diff.*, difference; *SD*, standard deviation from mean; *RSD*, percent standard deviation relative to mean of NADP and CAPMoN median values; *MMAD*, modified median absolute deviation between NADP and CAPMoN values; *CoV*, non-parametric coefficient of variation; *P90*, 90^th^ percentile value; Pearson’s *r*, coefficient for correlation of NADP and CAPMoN values; *p*-value, the probability of a null hypothesis is true. The null hypothesis here is that there is no difference between the means (or medians) of CAPMoN and NADP weekly concentrations. *p*-values shown are for Wilcoxon’s signed-rank test; SO_4_^2−^, sulfate; NO_3_^−^, nitrate; NH_4_^+^, ammonium; H^+^, hydrogen ion; Ca^2+^, calcium; Cl^−^, chloride; K^+^, potassium; Mg^2+^, magnesium; Na^+^, sodium. Data obtained from NADP, Wisconsin State Laboratory of Hygiene at https://nadp.slh.wisc.edu/networks/national-trends-network and Environment and Climate Change Canada at https://www.canada.ca/en/environment-climate-change/services/air-pollution/monitoring-networks-data/canadian-air-precipitation.html, last accessed August 2023]

	Mean of NADP	Mean of CAPMoN	Diff. of mean	Relative diff. of mean	Median of NADP	Median of CAPMoN	Diff. of median	Relative diff. of median	SD	RSD	MMAD	CoV	P90	Pearson *r*	*p*-value
Ion	mg L^−1^	mg L^−1^	mg L^−1^	%	mg L^−1^	mg L^−1^	mg L^−1^	%	mg L^−1^	%	mg L^−1^	%	mg L^−1^		
SO_4_^2−^	1.422	1.491	0.069	4.7	0.918	1.005	0.056	5.6	0.282	29.4	0.099	10.3	0.327	0.81	<0.001
NO_3_^−^	1.326	1.513	0.187	13.2	1.004	1.175	0.115	9.8	0.333	30.6	0.132	12.1	0.533	0.87	<0.001
NH_4_^+^	0.351	0.400	0.048	12.8	0.258	0.321	0.043	13.4	0.095	32.8	0.047	16.4	0.145	0.81	<0.001
H^+^	0.024	0.026	0.002	7.9	0.016	0.018	0.002	8.8	0.006	37.0	0.004	22.0	0.009	0.96	<0.001
Ca^2+^	0.170	0.191	0.021	11.4	0.104	0.118	0.013	11.0	0.088	79.5	0.025	22.7	0.101	0.83	<0.001
Cl^−^	0.126	0.159	0.033	23.0	0.084	0.103	0.015	14.6	0.059	63.4	0.019	20.6	0.095	0.88	<0.001
K^+^	0.023	0.027	0.004	17.5	0.015	0.018	0.003	16.7	0.018	109.4	0.006	35.9	0.020	0.54	<0.001
Mg^2+^	0.026	0.030	0.005	16.7	0.015	0.020	0.003	15.0	0.013	76.3	0.004	25.4	0.016	0.82	<0.001
Na^+^	0.055	0.071	0.016	24.8	0.029	0.037	0.005	13.5	0.032	96.0	0.009	27.0	0.047	0.89	<0.001

Depth	mm	mm	mm	%	mm	mm	mm	%	mm	%	mm	%	mm		
Ppt	23.0	24.2	1.1	4.8	17.8	18.4	0.6	3.1	1.8	9.7	1.1	5.9	2.8	0.95	<0.001

**Table 3:** Statistics for inter-comparisons of weekly ion concentrations and precipitation (Ppt) depths measured from co-located National Atmospheric Deposition Program (NADP) and Canadian Air and Precipitation Monitoring Network CAPMoN) sites at Frelighsburg, Quebec, Canada, during the period 2002–2011 [units are in milligrams per liter (mg L^−1^), percent (%), and millimeters (mm), or unitless (R, *p*-value), as indicated. *Diff.*, difference; *SD*, standard deviation from mean; *RSD*, percent standard deviation relative to mean of NADP and CAPMoN median values; *MMAD*, modified median absolute deviation between NADP and CAPMoN values; *CoV*, non-parametric coefficient of variation; *P90*, 90^th^ percentile value; Pearson’s *r*, coefficient for correlation of NADP and CAPMoN values; *p*-value, the probability of a null hypothesis is true. The null hypothesis here is that there is no difference between the means (or medians) of CAPMoN and NADP weekly concentrations. *p*-values shown are for Wilcoxon’s signed-rank test; *SO*_*4*_^*2−*^, sulfate; *NO*_*3*_^*−*^, nitrate; *NH*_*4*_^*+*^, ammonium; *H*^*+*^, hydrogen ion; *Ca*^*2+*^, calcium; *Cl*^*−*^, chloride; *K*^*+*^, potassium; *Mg*^*2+*^, magnesium; *Na*^*+*^, sodium. Data obtained from NADP, Wisconsin State Laboratory of Hygiene at https://nadp.slh.wisc.edu/networks/national-trends-network and Environment and Climate Change Canada at https://www.canada.ca/en/environment-climate-change/services/air-pollution/monitoring-networks-data/canadian-air-precipitation.html, last accessed August 2023]

	Mean of NADP	Mean of CAPMoN	Diff. of mean	Relative diff. of mean	Median of NADP	Median of CAPMoN	Diff. of median	Relative diff. of median	SD	RSD	MMAD	CoV	P90	Pearson’s *r*	*p*-value
Ion	mg L^−1^	mg L^−1^	mg L^−1^	%	mg L^−1^	mg L^−1^	mg L^−1^	%	mg L^−1^	%	mg L^−1^	%	mg L^−1^		
SO_4_^2−^	1.359	1.361	0.002	0.1	0.980	1.013	0.030	3.0	0.324	32.5	0.123	12.4	0.263	0.96	<0.001
NO_3_^−^	1.500	1.593	0.093	6.0	1.020	1.128	0.071	6.3	0.516	48.1	0.123	11.5	0.496	0.93	<0.001
NH_4_^+^	0.404	0.456	0.053	12.2	0.295	0.356	0.048	13.5	0.120	36.9	0.059	18.2	0.161	0.93	<0.001
H^+^	0.021	0.024	0.002	9.8	0.015	0.018	0.002	9.3	0.007	44.2	0.005	28.5	0.011	0.93	<0.001
Ca^2+^	0.187	0.198	0.011	5.9	0.107	0.114	0.008	7.0	0.133	120.3	0.033	29.5	0.085	0.75	<0.001
Cl^−^	0.111	0.109	−0.001	−1.0	0.052	0.060	0.003	5.0	0.075	133.8	0.015	26.5	0.036	0.83	<0.001
K^+^	0.019	0.024	0.004	20.3	0.013	0.017	0.003	17.7	0.018	118.2	0.007	49.4	0.019	0.57	<0.001
Mg^2+^	0.021	0.024	0.002	9.8	0.014	0.015	0.002	13.3	0.011	74.6	0.004	30.7	0.011	0.85	<0.001
Na^+^	0.059	0.057	−0.001	−2.0	0.020	0.024	0.001	4.2	0.052	234.8	0.006	27.0	0.021	0.81	<0.001

Depth	mm	mm	mm	%	mm	mm	mm	%	mm	%	mm	%	mm		
Ppt	24.6	27.0	2.4	9.2	19.6	22.0	1.7	7.7	2.1	9.9	1.7	8.0	4.7	0.97	<0.001

**Table 4:** Statistics for inter-comparisons of weekly ion concentrations and precipitation (Ppt) depths measured from co-located National Atmospheric Deposition Program (NADP) and Canadian Air and Precipitation Monitoring Network CAPMoN) sites at Frelighsburg, Quebec, Canada, during the period 2012–2019 [units are in milligrams per liter (mg L^−1^), percent (%), and millimeters (mm), or unitless (R, *p*-value), as indicated. *Diff.*, difference; *SD*, standard deviation from mean; *RSD*, percent standard deviation relative to mean of NADP and CAPMoN median values; *MMAD*, modified median absolute deviation between NADP and CAPMoN values; *CoV*, non-parametric coefficient of variation; *P90*, 90^th^ percentile value; Pearson’s *r*, coefficient for correlation of NADP and CAPMoN values; *p*-value, the probability of a null hypothesis is true. The null hypothesis here is that there is no difference between the means (or medians) of CAPMoN and NADP weekly concentrations. *p*-values shown are for Wilcoxon’s signed-rank test; *SO*_*4*_^*2−*^, sulfate; *NO*_*3*_^*−*^, nitrate; *NH*_*4*_^*+*^, ammonium; *H*^*+*^, hydrogen ion; *Ca*^*2+*^, calcium; *Cl*^*−*^, chloride; *K*^*+*^, potassium; *Mg*^*2+*^, magnesium; *Na*^*+*^, sodium. Data obtained from NADP, Wisconsin State Laboratory of Hygiene at https://nadp.slh.wisc.edu/networks/national-trends-network and Environment and Climate Change Canada at https://www.canada.ca/en/environment-climate-change/services/air-pollution/monitoring-networks-data/canadian-air-precipitation.html, last accessed August 2023]

	Mean of NADP	Mean of CAPMoN	Diff. of mean	Relative diff. of mean	Median of NADP	Median of CAPMoN	Diff. of median	Relative diff. of median	SD	RSD	MMAD	CoV	P90	Pearson’s *r*	*p*-value
Ion	mg L^−1^	mg L^−1^	mg L^−1^	%	mg L^−1^	mg L^−1^	mg L^−1^	%	mg L^−1^	%	mg L^−1^	%	mg L^−1^		
SO_4_^2−^	0.714	0.632	−0.082	−12.1	0.540	0.528	−0.017	−3.2	0.160	30.0	0.053	10.0	0.039	0.76	<0.001
NO_3_^−^	1.197	1.067	−0.131	−11.6	0.889	0.857	−0.028	−3.3	0.338	38.8	0.065	7.5	0.043	0.82	<0.001
NH_4_^+^	0.457	0.418	−0.039	−8.8	0.382	0.345	−0.011	−3.2	0.110	30.3	0.046	12.6	0.039	0.77	<0.001
H^+^	0.007	0.010	0.003	35.2	0.004	0.007	0.002	27.4	0.004	71.7	0.003	47.5	0.008	0.87	<0.001
Ca^2+^	0.201	0.151	−0.050	−28.1	0.128	0.113	−0.008	−6.6	0.123	101.9	0.021	17.2	0.024	0.53	<0.001
Cl^−^	0.139	0.091	−0.049	−42.3	0.057	0.053	−0.001	−1.9	0.149	270.7	0.010	18.9	0.012	0.54	<0.001
K^+^	0.026	0.028	0.002	7.0	0.016	0.016	0.002	12.9	0.025	159.2	0.004	28.2	0.008	0.12	<0.001
Mg^2+^	0.025	0.022	−0.003	−14.7	0.018	0.016	<0.001	0.0	0.011	62.4	0.004	26.6	0.005	0.57	<0.001
Na^+^	0.081	0.052	−0.029	−43.5	0.030	0.028	−0.001	−3.6	0.096	329.9	0.006	20.4	0.008	0.47	<0.001

Depth	mm	mm	mm	%	mm	mm	mm	%	mm	%	mm	%	mm		
Ppt	25.0	25.2	0.2	0.7	19.8	20.7	0.7	3.2	2.1	10.3	1.1	5.5	2.6	0.77	<0.001

#### Penn State site, PA15/PEN, 1986–2019

The statistics for the Penn State site during 1986–2019 are shown in Table 1 and Table S.2. Among SO_4_^2−^, NO_3_^−^, NH_4_^+^, and H^+^, the relative differences of the median ranged from 0.003 mg L^−1^ for H^+^ to 0.148 mg L^−1^ for NO_3_^−^. In units of microequivalents per liter (μeq L^−1^), it ranged from 1.37 μeq L^−1^ for SO_4_^2−^ to 3.02 μeq L^−1^ for H^+^. The relative differences were 3.7%, 8.8%, 16%, and 7.6% for SO_4_^2−^, NO_3_^−^, NH_4_^+^, and H^+^, respectively. During the warm (cold) season, the corresponding relative differences were 1.9% (6.6%), 5.4% (12.8%), 13.6% (18.5%), and 5.6% (9.4%). The relative differences were smaller during the warm season than the cold season, especially for SO_4_^2−^, NO_3_^−^, and H^+^. The relative standard deviations were between 24.0% for SO_4_^2−^, and 35.1% for NH_4_^+^. The spread of the one-to-one scatter plots (not shown) is quantitatively described by the metrics of CoV. The CoV for each ion was much smaller than the relative standard deviation, ranging from 9.5% for SO_4_^2−^ to 18.3% for NH_4_^+^. The correlation coefficient was between 0.83 for NH_4_^+^ and 0.95 for SO_4_^2−^. In general, there was a good correlation between CAPMoN and NADP for the weekly concentrations of SO_4_^2−^, NO_3_^−^, NH_4_^+^, and H^+^. The correlation coefficients for the warm and cold seasons were comparable for SO_4_^2−^, NO_3_^−^, and H^+^. However, the correlation coefficient for NH_4_^+^ was 0.92 for the cold season, higher than 0.76 for the warm season.

**Fig. 1:**
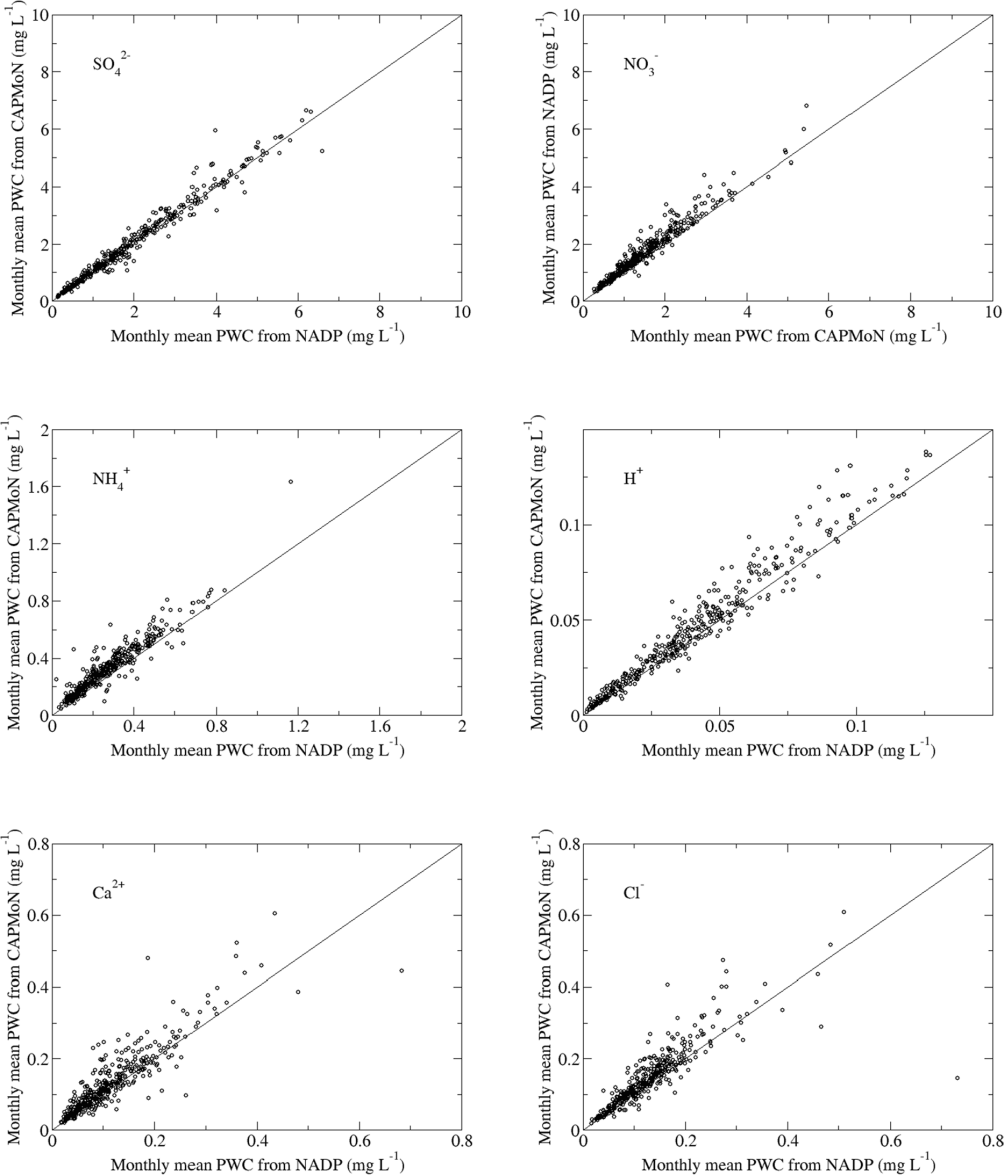

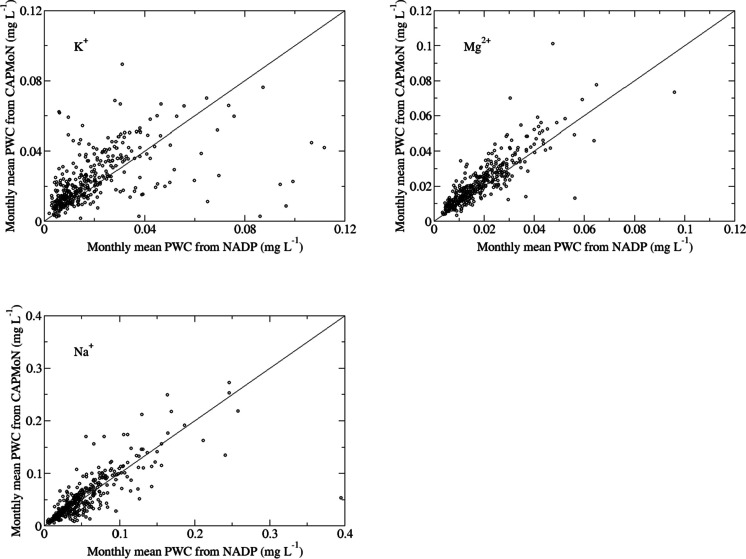
Monthly precipitation-weighted mean concentrations (PWC) in milligrams per liter (mg L^−1^) from National Atmospheric Deposition Program (NADP) and Canadian Air and Precipitation Monitoring Network (CAPMoN) for co-located measurements at Pennsylvania State University, PA, USA, from 1986 to 2019. One-to-one line is shown for evaluation of bias

For base cations and Cl^−^, the median differences ranged from 0.002 mg L^−1^ for Na^+^ to 0.013 mg L^−1^ for Cl^−^. In units of μeq L^−1^, it ranged from 0.09 μeq L^−1^ for Na^+^ to 0.60 μeq L^−1^ for Ca^2+^. The relative differences were between 5.6% for Na^+^ and 21.1% for K^+^. The CoV was between 19.7% for Cl^−^ and 44.9% for K^+^, indicating generally the one-to-one comparisons of weekly concentration were more scattered for base cations and Cl^−^ than for SO_4_^2−^, NO_3_^−^, NH_4_^+^ and H^+^. Except for K^+^, there was a strong correlation for the weekly concentration between CAPMoN and NADP for base cations and Cl^−^, with the correlation coefficient ranging from 0.84 to 0.91. For Ca^2+^, Cl^−^, and Na^+^, the correlation coefficients were higher during the warm season than the cold season. As shown in Table 1, *p*-values were less than 0.05 for all ions indicating that the weekly concentrations from CAPMoN were statistically higher than those from NADP. Scatter plots of precipitation-weighted monthly mean concentrations, instead of weekly concentrations for better visualization, for the Penn State site are shown in Fig. 1. Corresponding linear regression coefficients of precipitation-weighted monthly mean concentrations of CAPMoN-vs-NADP are provided in Table S.1 for the Penn State site, as well as the Frelighsburg site. The monthly mean values were calculated analogously to Eqs. 1 and 3, using all weekly samples with start dates within the calendar months. Among all ions, data for SO_4_^2−^ had the best agreement between the two networks. The spread of the one-to-one comparison for NO_3_^−^, NH_4_^+^, and H^+^ was small, but there was an obvious positive bias for each ion. The spread of the plot for K^+^ was largest among all ions, and the spreads for Ca^2+^, Mg^2+^, Cl^−^, and Na^+^ were comparable.

Overall, the relative differences of the weekly concentrations between CAPMoN and NADP were less than 5% for SO_4_^2−^; 5–10% for NO_3_^−^, H^+^, and Cl^−^; 10–16% for Ca^2+^, Mg^2+^, and NH_4_^+^; and 21% for K^+^. The correlation coefficients were greater than 0.9 for SO_4_^2−^, NO_3_^−^, H^+^, and Cl^−^; between 0.8 and 0.9 for NH_4_^+^, Ca^2+^, Mg^2+^, and Na^+^; and less than 0.5 for K^+^. The between-network biases were statistically significant (*p* < 0.05) for all ions.

#### Penn State site, PA15/PEN, 2005–2019

To compare with the analysis of pre-2005 data presented in Sirois et al. (2000) and Wetherbee et al. (2010), we also carried out the NADP-CAPMoN inter-comparison at the Penn State site for 2005–2019 only. As shown in Tables 2, the median and arithmetic mean weekly concentrations of SO_4_^2−^, NO_3_^−^, NH_4_^+^, and H^+^ were lower during 2005–2019 than 1986–2019, due to emissions reductions of SO_2_ and NO_x_ in the eastern US and Canada since 1990 (Feng et al., 2020). There were no significant differences in terms of relative bias, in comparing 2005–2019 to 1986–2019. For all seasons, the relative bias was lowest for SO_4_^2−^ (5.6%), close to 10% for NO_3_^−^ and H^+^, and between 10 and 17% for NH_4_^+^, Ca^2+^, Mg^2+^, K^+^, Na^+^, and Cl^−^. The correlation coefficients were generally lower during 2005–2019, and it was greater than 0.9 for H^+^ only. This could be due to the aging of the sample collectors, either CAPMoN or NADP collectors, or both. The between-network biases were statistically significant for all ions. The two networks agreed better during the warm season, with the average relative bias of 8.4% during the warm season (Table S.2c), compared to 15.9% during the cold season (Table S.2d).

#### Frelighsburg site, CAN5/FRE, 2002–2011

The NADP sample collector at Frelighsburg was changed from the ACM collector to the NCON collector in October 2011 (Wetherbee, 2017). Therefore, the statistical analysis for the collocated measurements at Frelighsburg was carried out for the two periods, 2002–2011 and 2012–2019, to account for the differences in collectors. The summaries of the statistical analysis are presented in Table 3 and Table 4 for 2002–2011 and 2012–2019, respectively.

During 2002–2011, for all seasons, the relative differences were 3.0%, 6.3%, 9.3%, and 13.5% for SO_4_^2−^, NO_3_^−^, H^+^, and NH_4_^+^, respectively. For base cations and Cl^−^, the relative differences varied from 4.2% for Na^+^ to 17.7% for K^+^. The spread of the one-to-one scatter plot (not shown) of weekly CAPMoN-vs-NADP concentrations was low for SO_4_^2−^, NO_3_^−^, and NH_4_^+^, with CoV ranging from 11.5 to 18.2%. The spread was relatively high for H^+^, Ca^2+^, Cl^−^, Mg^2+^, and Na^+^, with CoV ranging from 27.0% for Na^+^ to 30.7% for Mg^2+^, and K^+^ had the highest spread with CoV of 49.4%. The biases between CAPMoN and NADP were not statistically significant for SO_4_^2−^, Ca^2+^, Na^+^, and Cl^−^, but were statistically significant for other ions from the *t*-test (not shown). However, the biases were statistically significant from the Wilcoxon signed-rank test, as indicated by the *p*-values in Table 3. The correlations of the weekly concentrations were of similar strength for SO_4_^2−^, NO_3_^−^, NH_4_^+^, and H^+^, with *r* of 0.93–0.96. For base cations and Cl^−^, *r* ranged from 0.75 to 0.85, except for K^+^ with *r* of 0.57. The correlation coefficients for the warm season were higher than the cold season (Table S.2e and f). During the warm season, there were high correlations for all ions (*r* = 0.94–0.98) except for K^+^.

Overall, CAPMoN has higher weekly concentrations than NADP for 2002–2011 at Frelighsburg. The relative bias was small for SO_4_^2−^, NO_3_^−^, H^+^, Ca^2+^, Cl^−^, and Na^+^, ranging from 3.0 to 9.3%; moderate for NH_4_^+^, Mg^2+^, and K^+^, from 13.3% for Mg^2+^ to 17.7% for K^+^.

#### Frelighsburg, CAN5/FRE, 2012–2019

For 2012–2019, the NCON sampler was used as the NADP sample collector at Frelighsburg. The inter-comparisons of collocated measurements at 12 sites in the USA during 2009–2015 by Wetherbee (2017) showed that overall the weekly concentrations sampled with NCON collectors, compared to the ACM collectors, were 30% higher for K^+^, of no difference for H^+^, and 20% higher for other ions. For the current inter-comparison of CAPMoN-vs-NADP, we found that the relative biases changed from positive values during 2002–2011 to negative values during 2012–2019 for all season and cold season except for H^+^ and K^+^ (Table 4 and Table S.2g-h) Excluding H^+^ and K^+^, the relative biases during 2012–2019 varied from −6.6 to −1.9% for all season, and from −14.0 to − 5.0% for the cold season. During the warm season, only SO_4_^2−^, NO_3_^−^, and Ca^2+^ had negative biases, and the absolute values of the relative biases were small for all ions except for H^+^, ranging from <0.1% for Mg^2+^ and Na^+^ to 5.6% for K^+^. During the cold season, the negative biases for all ions were statistically significant. During the warm season, only negative bias for NO_3_^−^ (−1.5%) and positive bias for H^+^ (43.0%) were statistically significant. Comparing 2012–2019 to 2002–2011, the average correlation coefficient for all ions decreased from 0.91 to 0.75 during the warm season, and from 0.81 to 0.60 during the cold season.

### Time series and trends of relative bias

#### Penn State site, PA15/PEN

**Fig. 2:**
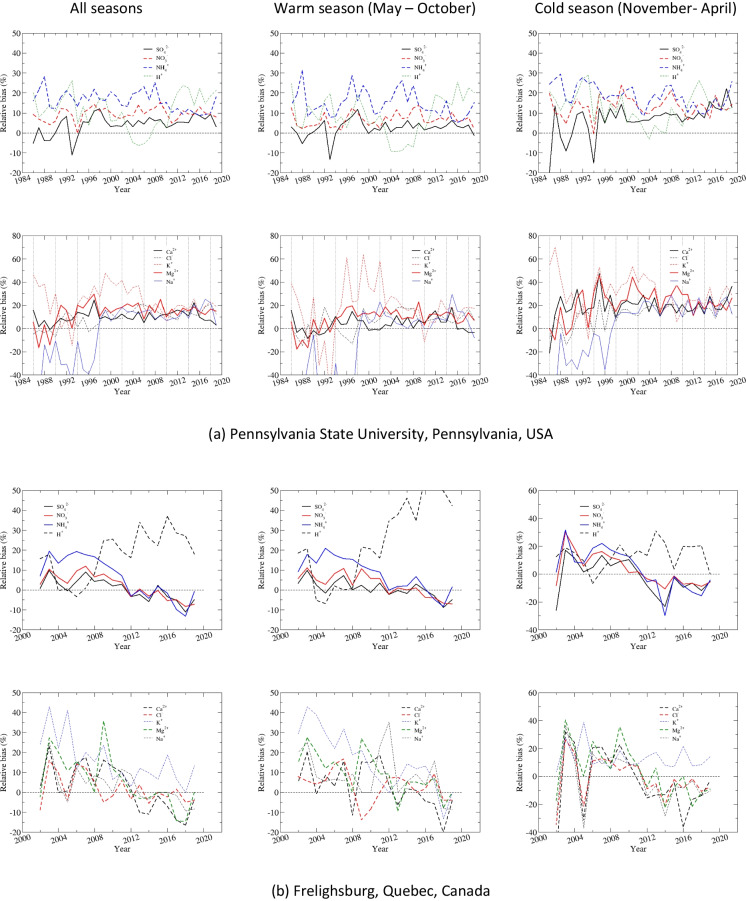
Time series of annual and seasonal relative bias (%) of National Atmospheric Deposition Program (NADP) measurements relative to Canadian Air and Precipitation Monitoring Network (CAPMoN) measurements of weekly concentrations for co-located sample collectors at **a** Pennsylvania State University, PA, USA, 1986–2019 and **b** Frelighsburg, Quebec, Canada, 2002–2019. The relative bias is derived with Eq. 5 for each year

Time series of relative bias for each year are shown in Fig. 2a. The Mann-Kendall (M-K) test (Feng et al., 2021; Mann, 1945) was applied to the annual relative biases to detect if an increasing/decreasing trend exists. Two metrics of the M-K test, *p*-value and *tau*, were used to identify if the trend was statistically significant and the strength of the trend. For SO_4_^2−^, the relative bias before 1998 varied much more than after 1998. During 1986–1998, the relative bias ranged from −11.2% in 1993 to 12.0% in 1998, with a mean value of 1.8% and standard deviation of 6.9%. During 1999–2019, the relative differences of SO_4_^2−^ varied less (3.1 to 9.9%), with a mean value of 5.5% and a standard deviation of 2.1%. There was a moderate increasing trend in the relative bias during 1999–2019, mainly due to cold season biases during 2010–2019. The relative biases during the warm season for 1999–2019 were small, with a mean value of 2.8% ± 1.9% versus 9.6% ± 4.1% during the cold season. The trend of the relative bias of NO_3_^−^ was not statistically significant with *p* = 0.99 for the cold season, *p* = 0.24 for the warm season, and *p* = 0.39 for all seasons. The mean and standard deviation annual relative biases for each season were 9.4% ± 3.2% (all), 3.4% ± 4.9% (warm), and 13.3% ± 4.9% (cold). The M-K test for the trend in the annual relative bias for NH_4_^+^ indicated no significant trends for 1986–2010 and 2011–2019. However, there was a clear decrease of the relative bias from 2008 to 2011. The mean relative bias values for 1986–2010 and 2011–2019 were 18.0% and 11.1%, respectively. Seasonally, there was a weak decreasing (*p* = 0.05) and a weak increasing trend (*p* = 0.09) of the relative bias for 1986–2010 and 2011–2019 during the cold seasons. The relative bias during the warm season (18.7%) was higher than the cold season (14.7%). The relative bias of H^+^ had a decreasing trend (*p*=0.02) for 1996–2005, an increasing trend (*p*=0.02) for 2005–2011, and no trend for 2011–2019 (*p*=0.9). The relative bias of H^+^ turned negative during 2004–2007 for all seasons, during 2004–2008 for the warm season, and was negative only in 2004 for the cold season. Overall, for the recent one to two decades, the measured weekly concentrations from CAPMoN were higher than NADP at Penn State for SO_4_^2−^ (5.5%) and NO_3_^−^ (9.4%) during 1999–2019, and NH_4_^+^ (11.1%) during 2011–2019.

For base cations and Cl^−^, the relative biases had an increasing trend for Ca^2+^ and Mg^2+^ (*p* = 0.06–0.09), and a weak increasing trend for Cl^−^ (*p* = 0.2) during 1986–1997. During 1998–2019, the trends were flat for Ca^2+^, Mg^2+^, and Cl^−^. There is a known bias in Na^+^ concentration data collected by NADP before 1998 due to sample contamination (Wetherbee et al., 2010) and the values of Na^+^ from NADP were significantly (α=0.05) lower than CAPMoN during 1986–1998. Relative biases were positive during all seasons for Ca^2+^, Mg^2+^, Cl^−^, and Na^+^ during 1998–2019, except for a few years for Ca^2+^ during the warm seasons, with the mean values for the relative bias varying from 10.5 to 16.0%, and the standard deviations from 4.2 to 5.5%. For 1998–2019, the mean relative bias during the warm season were 4.4–11.4% for Ca^2+^, Mg^2+^, Na+, and Cl^−^, much lower than the values of the cold season (16.3–25.0%). For K^+^, there was a decreasing trend in relative bias for 1986–1993, an increasing trend for 1993–1999, a decreasing trend for 1999–2005, and no trend for 2005–2019. The corresponding mean relative bias and standard deviation during 2005-2019 was 17.4% ± 5.1%.

Overall, since 1998 for Ca^2+^, Cl^−^, Mg^2+^, and Na^+^, and since 2005 for K^+^, the relative biases between CAPMoN and NADP were generally constant. The weekly concentrations measured by CAPMoN were 10.5 to 17.4% higher. The relative biases during the warm season were much lower than during the cold season.

#### Frelighsburg, CAN5/FRE

As shown in Fig. 2b, the relative biases for SO_4_^2−^, NO_3_^−^, and NH_4_^+^ were highly correlated to each other (*R* > 0.89) at the Frelighsburg site. The M-K test indicated no trends for SO_4_^2−^ and NO_3_^−^, and a weak decreasing trend for NH_4_^+^ during 2002–2011, which was prior to the precipitation sampler changed from the ACM collector to the NCON collector in October 2011. For 2012–2019, there was a decreasing trend in relative bias for NO_3_^−^, and there were no statistically significant trends for SO_4_^2−^ and NH_4_^+^. The relative biases have changed from generally positive to generally negative since the sampler was changed. During 2002–2011, the mean values of the relative biases were 4.0% (SO_4_^2−^), 6.7% (NO_3_^−^), and 14.2% (NH_4_^+^), compared to −4.3% (SO_4_^2−^), −4.0% (NO_3_^−^), and −4.0% (NH_4_^+^) during 2012–2019. For 2002–2019, the agreement between CAPMoN and NADP was very good during the warm season, with low relative biases of −2.3% (SO_4_^2−^), −2.7% (NO_3_^−^), and −0.1% (NH_4_^+^). For the cold season, the relative biases were relatively larger: −10.5% (SO_4_^2−^), −6.3% (NO_3_^−^), and −10.4% (NH_4_^+^). This indicates that when the new NCON collector was deployed at the Frelighsburg site, the previous positive biases changed to negative biases. The biases were relatively small for the warm season, but greater during the cold season. For H^+^, the trend of the relative bias was similar to that at Penn State: there was a decreasing trend for 2002–2005, and a significant (α=0.05) increasing trend for 2005–2019, especially during the warm season. However, during the cold season, there was no statistically significant (α=0.05) trend during 2008–2019.

For base cations and Cl^−^, there were no statistically significant trends for Ca^2+^, Mg^2+^, and Cl^−^ during 2002–2011 (*p* > 0.7) and for 2012–2019 (*p* > 0.3). For Na^+^, there was no trend in relative bias for 2002–2011, but there was a weak decreasing trend for 2012–2019. For K^+^, there was a decreasing trend in relative bias for 2002–2011, but then it was mostly constant for 2012–2019. Comparing the periods before and after the collector change , the mean values of the relative biases changed from positive (2002–2011) to negative (2012–2019) from 9.9 to −7.8% (Ca^2+^), 13.6 to −4.4% (Mg^2+^), 7.0 to −1.9% (Na^+^), and 4.4 to −1.9% (Cl^−^). For K^+^, the mean relative bias changed from 21.8 to 8.7%. However, during the warm season, only Ca^2+^ had a negative mean relative bias during 2012–2019 (−4.3%), and positive mean relative biases varied from 1.6 to 6.6% for Mg^2+^, K^+^, Na^+^, and Cl^−^. Overall, with the NCON collector, the agreement between CAPMoN and NADP for base cations and Cl^−^ was much better than the previous ACM sampler during the warm season, but for the cold season, except for K^+^, the relative biases changed from positive values to negative values and the absolute values remained relatively large.

### Precipitation-weighted annual and seasonal mean concentrations

#### Penn State site, PA15/PEN

**Fig. 3:**
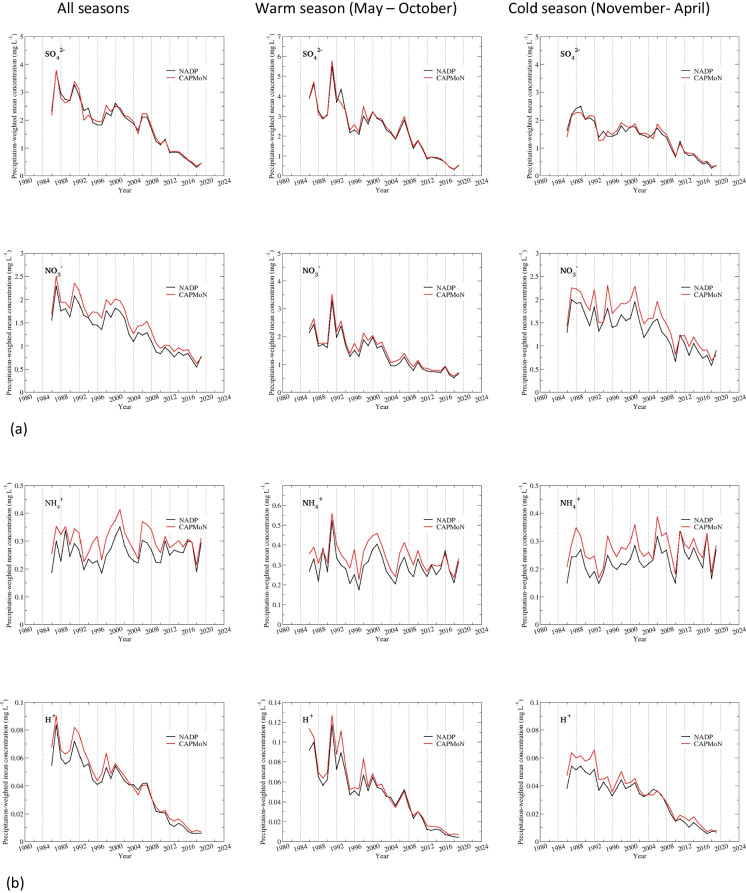

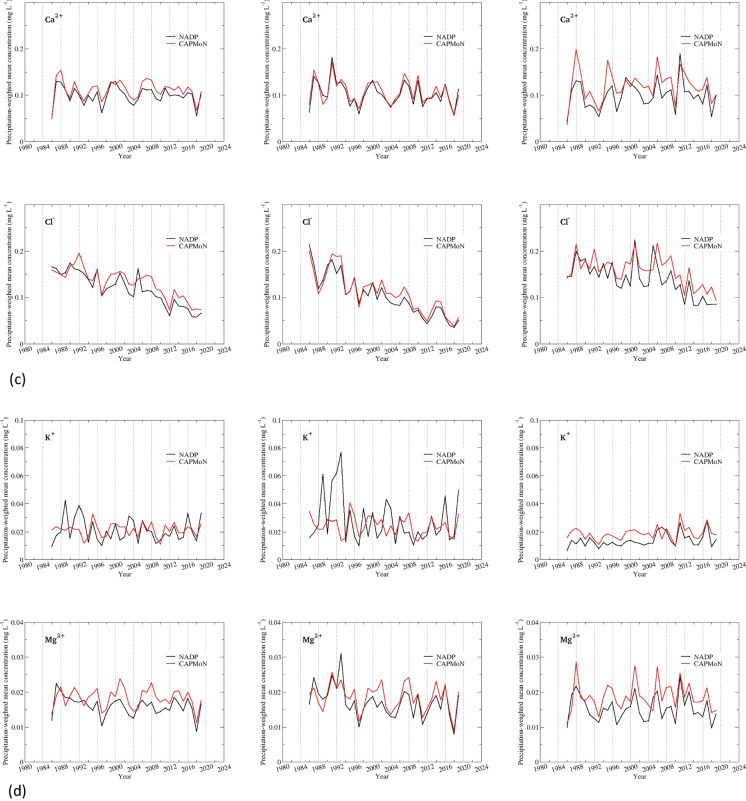

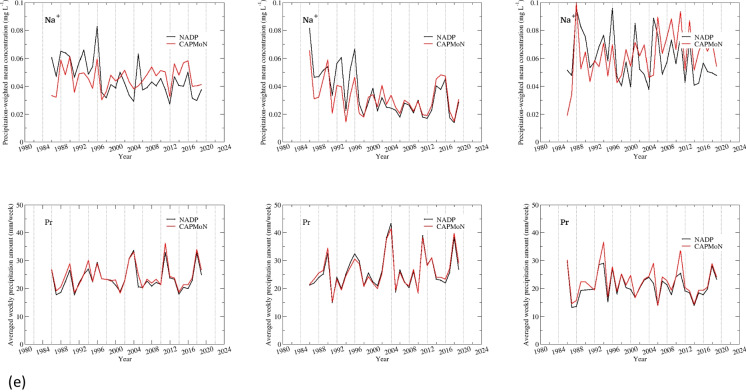
**a**–**e** Precipitation-weighted annual and seasonal mean concentrations (milligrams per liter, mg L^−1^) and annual mean weekly precipitation depths (millimeters per week, mm/week) for National Atmospheric Deposition Program (NADP) and Canadian Air and Precipitation Monitoring Network (CAPMoN) for co-located monitoring sites at Pennsylvania State University, PA, USA, 1986–2019

Time series of precipitation-weighted annual and seasonal mean concentrations are shown in Fig. 3 for Penn State, and the summary of statistics is presented in Tables 5 and 6 and Table S.3. SO_4_^2−^ had a good agreement between CAPMoN and NADP for both annual and seasonal precipitation-weighted mean concentrations, with relative biases ranging 2.1–3.8%, CoV ranging 3.5–8.3%, and *r* >= 0.98. The agreement was exceptionally good for the warm season after 2000. Data for NO_3_^−^ and NH_4_^+^ indicated consistent positive biases over the 1986–2019 period, 11.8% and 15.6%, respectively. The relative bias was comparable between the warm and cold season for NH_4_^+^, but was much smaller during the warm season than the cold season for NO_3_^−^. The correlation coefficients were greater than 0.97 for NO_3_^−^, and around 0.90 for NH_4_^+^, for both warm and cold seasons. The CoV for NO_3_^−^ was exceptionally small (5.3%) for the warm season, indicating the agreement between CAPMoN and NADP would be very good if the biases were eliminated. The statistical metrics in Tables 5 and 6 show that the agreement between CAPMoN and NADP for H^+^ was good with respect to relative bias (6.2%), CoV (8.7%), and correlation (*r*=0.99). However, after examining the time series of annual and seasonal precipitation-weighted mean concentrations for H^+^ (Fig. 3), we found that there were relatively high biases before 2000 and after 2011. The relative biases for 2000–2011 were small, especially during the warm season. The time series of relative differences between CAPMoN and NADP for H^+^ indicates that the relative difference might be linked to buffering of the acidity from organic acids and carbonates in precipitation samples. When daily samples with different acidity are mixed together to create a weekly composite, it might affect particle dissolution and disassociation of organic acids, and therefore affect the concentrations of base cations and H^+^. This buffering capacity might have changed over the study period when the overall acidity of precipitation samples decreased over the period.

**Table 5:** Statistics for inter-comparisons of precipitation-weighted annual mean concentrations and annual mean weekly precipitation depths from co-located National Atmospheric Deposition Program (NADP) and Canadian Air and Precipitation Monitoring Network CAPMoN) sites at Pennsylvania State University, PA, USA, during period 1986–2019. *p*-values shown are derived with *t*-test [units are in milligrams per liter (mg L^−1^), percent (%), and millimeters (mm), or unitless (Perason’s *r*, *p*-value), as indicated. *Diff.*, difference; *SD*, standard deviation from mean; *RSD*, percent standard deviation relative to mean of NADP and CAPMoN median values; *MMAD*, modified median absolute deviation between NADP and CAPMoN values; *CoV*, non-parametric coefficient of variation; *P90*, 90^th^ percentile value; Pearson’s *r*, coefficient for correlation of NADP and CAPMoN values; *p*-values are for *t*-test whereby the probability of a null hypothesis is true. The null hypothesis here is that there is no difference between the means (or medians) of CAPMoN and NADP weekly concentrations; *SO*_*4*_^*2−*^, sulfate; *NO*_*3*_^*−*^, nitrate; *NH*_*4*_^*+*^, ammonium; *H*^*+*^, hydrogen ion; *Ca*^*2+*^, calcium; *Cl*^*−*^, chloride; *K*^*+*^, potassium; *Mg*^*2+*^, magnesium; *Na*^*+*^, sodium. Data obtained from NADP, Wisconsin State Laboratory of Hygiene at https://nadp.slh.wisc.edu/networks/national-trends-network and Environment and Climate Change Canada at https://www.canada.ca/en/environment-climate-change/services/air-pollution/monitoring-networks-data/canadian-air-precipitation.html, last accessed August 2023]

	Mean of NADP	Mean of CAPMoN	Diff. of mean	Relative diff. of mean	Median of NADP	Median of CAPMoN	Diff. of median	Relative diff. of median	SD	RSD	MMAD	CoV	P90	Pearson’s *r*	*p*-value
Ion	mg L^−1^	mg L^−1^	mg L^−1^	%	mg L^−1^	mg L^−1^	mg L^−1^	%	mg L^−1^	%	mg L^−1^	%	mg L^−1^		
SO_4_^2−^	1.834	1.860	0.027	1.4	1.952	2.017	0.044	2.2	0.126	6.8	0.124	6.7	0.127	0.99	0.220
NO_3−_	1.326	1.494	0.168	11.9	1.321	1.565	0.184	11.8	0.079	5.6	0.075	5.4	0.270	0.99	<0.001
NH_4_^+^	0.259	0.304	0.045	16.1	0.259	0.303	0.048	15.9	0.025	8.8	0.031	11.0	0.075	0.85	<0.001
H^+^	0.038	0.042	0.004	9.6	0.042	0.043	0.003	6.2	0.004	10.7	0.004	8.7	0.010	0.99	<0.001
Ca^2+^	0.099	0.110	0.011	10.8	0.100	0.110	0.012	11.1	0.009	8.54	0.010	9.5	0.022	0.90	<0.001
Cl^−^	0.118	0.131	0.013	10.2	0.116	0.139	0.015	10.5	0.014	11.03	0.014	10.6	0.029	0.92	<0.001
K^+^	0.021	0.021	<0.001	1.7	0.019	0.022	0.003	14.9	0.009	40.32	0.008	39.9	0.009	0.27	0.804
Mg^2+^	0.016	0.018	0.002	13.3	0.016	0.019	0.002	13.3	0.002	12.5	0.002	10.2	0.005	0.70	<0.001
Na^+^	0.046	0.046	<0.001	−0.4	0.043	0.047	0.005	10.9	0.012	25.77	0.013	30.0	0.010	0.45	0.921

Depth	mm	mm	mm	%	mm	mm	mm	%	mm	%	mm	%	%		
Ppt	23.4	24.3	0.9	3.6	22.6	23.2	0.7	2.9	1.2	5.1	1.3	5.5	2.3	0.96	<0.001

**Table 6:** Statistics for inter-comparisons of precipitation-weighted annual mean concentrations and annual mean weekly precipitation depths from co-located National Atmospheric Deposition Program (NADP) and Canadian Air and Precipitation Monitoring Network CAPMoN) sites at Pennsylvania State University, PA, USA during period 2005–2019. *p*-values shown are derived with *t*-test [units are in milligrams per liter (mg L^−1^), percent (%), and millimeters (mm), or unitless (Pearson’s *r*, *p*-value), as indicated. *Diff.*, difference; *SD*, standard deviation from mean; *RSD*, percent standard deviation relative to mean of NADP and CAPMoN median values; *MMAD*, modified median absolute deviation between NADP and CAPMoN values; *CoV*, non-parametric coefficient of variation; *P90*, 90^th^ percentile value; Pearson’s *r*, coefficient for correlation of NADP and CAPMoN values; *p*-values are for *t*-test whereby the probability of a null hypothesis is true. The null hypothesis here is that there is no difference between the means (or medians) of CAPMoN and NADP weekly concentrations; *SO*_*4*_^*2−*^, sulfate; *NO*_*3*_^*−*^, nitrate; *NH*_*4*_^*+*^, ammonium; *H*^*+*^, hydrogen ion; *Ca*^*2+*^, calcium; *Cl*^*−*^, chloride; *K*^*+*^, potassium; *Mg*^*2+*^, magnesium; *Na*^*+*^, sodium. Data obtained from NADP, Wisconsin State Laboratory of Hygiene at https://nadp.slh.wisc.edu/networks/national-trends-network and Environment and Climate Change Canada at https://www.canada.ca/en/environment-climate-change/services/air-pollution/monitoring-networks-data/canadian-air-precipitation.html, last accessed August 2023]

	Mean of NADP	Mean of CAPMoN	Diff. of mean	Relative diff. of mean	Median of NADP	Median of CAPMoN	Diff. of median	Relative diff. of median	SD	RSD	MMAD	CoV	P90	Pearson’s *r*	*p*-value
Ion	mg L^−1^	mg L^−1^	mg L^−1^	%	mg L^−1^	mg L^−1^	mg L^−1^	%	mg L^−1^	%	mg L^−1^	%	mg L^−1^		
SO_4_^2−^	1.073	1.110	0.039	3.5	0.837	0.877	0.041	4.7	0.062	5.7	0.044	4.0	0.118	1.00	0.031
NO_3_^−^	0.913	1.030	0.121	12.4	0.864	0.960	0.119	12.4	0.071	7.2	0.064	6.6	0.214	0.98	<0.001
NH_4_^+^	0.263	0.300	0.032	11.6	0.267	0.295	0.024	8.3	0.024	8.5	0.025	8.8	0.067	0.83	<0.001
H^+^	0.019	0.020	0.001	5.9	0.013	0.016	0.002	9.2	0.002	10.7	0.002	11.5	0.004	0.99	0.050
Ca^2+^	0.099	0.111	0.012	11.3	0.100	0.111	0.013	11.6	0.008	7.7	0.010	9.0	0.020	0.88	<0.001
Cl^−^	0.090	0.107	0.017	16.9	0.081	0.103	0.018	17.2	0.013	13.3	0.007	7.4	0.031	0.89	<0.001
K^+^	0.020	0.021	0.001	6.4	0.019	0.020	0.003	15.1	0.005	23.4	0.005	23.3	0.006	0.74	0.310
Mg^2+^	0.016	0.018	0.003	15.2	0.016	0.018	0.003	13.9	0.002	9.5	0.001	7.1	0.004	0.81	<0.001
Na^+^	0.041	0.047	0.007	15.1	0.040	0.048	0.008	17.0	0.009	19.9	0.004	9.2	0.012	0.43	0.011

Depth	mm	mm	mm	%	mm	mm	mm	%	mm	%	mm	%	%		
Ppt	23.1	24.3	1.2	5.1	22.1	23.2	1.0	4.5	1.2	5.2	0.8	3.2	2.6	0.97	0.002

For base cations and Cl^−^, very good agreements were found during the warm season for Ca^2+^ and Cl^−^ during 1986–2019, and for Mg^2+^ and Na^+^ during 2005–2019, with relative biases between 5.3 and 8.0% and *r* > 0.93 (Fig. 3 and Table S.3). During the cold season, there were consistent positive biases for all base cations and Cl^−^, except for Na^+^. During 2005–2019, relative biases for base cations and Cl^−^ ranged 17.9–22.0% during the cold season and 6.2–11.5% during the warm season (Tables S.3c and d). Note that during 2005–2019, the agreement between CAPMoN and NADP for Na^+^ was relatively poor during the cold season, as indicated by the relative bias (22.0%) and the correlation coefficient (0.5).

#### Frelighsburg, CAN5/FRE

For Frelighsburg, separate statistical analyses were done for 2002–2011 and 2012–2019 due to the change of the NADP sample collector. The summary of statistical analysis is presented in Tables 7 and 8 and Table S.3. During 2002–2011, the annual precipitation-weighted mean concentrations from CAPMoN were higher than those from NADP for all ions. The relative biases were 4.1% for SO_4_^2−^ and 5.9% for H^+^, and ranged 13.5–19.0% for other ions (Table 7). The CoV was small for SO_4_^2−^, NO_3_^−^, and NH_4_^+^, ranging from 4.2 to 6.4%, and 12.6–26.2% for other ions. There were strong correlations between CAPMoN and NADP for SO_4_^2−^, NO_3_^−^, NH_4_^+^, and H^+^ (*r* = 0.88–0.99). For base cations and Cl^−^, *r* ranged from 0.61 to 0.82. The relative biases for the cold season were higher than the warm season except for NH_4_^+^ and Mg^2+^, and the averages of the relative biases were 10.4% and 15.1% for the warm and cold seasons, respectively. The relative biases changed from positive values during 2002–2011 to negative values during 2012–2019 except for H^+^. During 2012–2019, the relative biases ranged from −5.5 to −3.9% for SO_4_^2−^, NO_3_^−^, and NH_4_^+^ (Table 8). The relative biases ranged from −15.0 to −11.1% for Ca^2+^, K^+^, Na^+^, and Cl^−^, and −3.2% for Mg^2+^. The agreement of the seasonal precipitation-weighted mean concentrations was very good for the warm season, except for H^+^ and K^+^. The relative differences were −1.4 to 2.2% for SO_4_^2−^, NO_3_^−^, NH_4_^+^, Cl^−^, and Mg^2+^, and −4.8% for Ca^2+^ during the warm season (Table S.3g). The correlation coefficients were 0.90–1.0 for all ions except for K^+^ (*r* = 0.68). There was no statistically significant bias between CAPMoN and NADP except for H^+^ during the warm season of 2012–2019. During the cold season of 2012–2019, excluding H^+^ and K^+^, the relative biases were −19.2 to −7.4% with an average of −14.1% (Table S.3h). For H^+^ and K^+^, the relative biases were 18.8% and 8.7%, respectively. Excluding K^+^ (*r* = 0.70 and 0.47 for the warm and the cold seasons), the mean correlation coefficient reduced to 0.44 during the cold season, comparing to 0.95 during the warm season. These results indicate that, with the implementation of the NCON collectors by NADP, the agreement between CAPMoN and NADP improved significantly during the warm season, but during the cold season, relatively large biases existed.

**Table 7:** Statistics for inter-comparisons of precipitation-weighted annual mean concentrations and annual mean weekly precipitation depths from co-located National Atmospheric Deposition Program (NADP) and Canadian Air and Precipitation Monitoring Network CAPMoN) sites at Frelighsburg, Quebec, Canada, during the periods 2002–2011. *p*-values shown are derived with *t*-test [units are in milligrams per liter (mg L^−1^), percent (%), and millimeters (mm), or unitless (Pearson’s *r*, *p*-value), as indicated. *Diff.*, difference; *SD*, standard deviation from mean; *RSD*, percent standard deviation relative to mean of NADP and CAPMoN median values; *MMAD*, modified median absolute deviation between NADP and CAPMoN values; *CoV*, non-parametric coefficient of variation; *P90*, 90^th^ percentile value; Pearson’s *r*, coefficient for correlation of NADP and CAPMoN values; *p*-values are for *t*-test whereby the probability of a null hypothesis is true. The null hypothesis here is that there is no difference between the means (or medians) of CAPMoN and NADP weekly concentrations; *SO*_*4*_^*2−*^, sulfate; *NO*_*3*_^*−*^, nitrate; *NH*_*4*_^*+*^, ammonium; *H*^*+*^, hydrogen ion; *Ca*^*2+*^, calcium; *Cl*^*−*^, chloride; *K*^*+*^, potassium; *Mg*^*2+*^, magnesium; *Na*^*+*^, sodium. Data obtained from NADP, Wisconsin State Laboratory of Hygiene at https://nadp.slh.wisc.edu/networks/national-trends-network and Environment and Climate Change Canada at https://www.canada.ca/en/environment-climate-change/services/air-pollution/monitoring-networks-data/canadian-air-precipitation.html, last accessed August 2023]

	Mean of NADP	Mean of CAPMoN	Diff. of mean	Relative diff. of mean	Median of NADP	Median of CAPMoN	Diff. of median	Relative diff. of median	SD	RSD	MMAD	CoV	P90	Pearson’s *r*	*p*-value
Ion	mg L^−1^	mg L^−1^	mg L^−1^	%	mg L^−1^	mg L^−1^	mg L^−1^	%	mg L^−1^	%	mg L^−1^	%	mg L^−1^		
SO_4_^2−^	1.108	1.140	0.032	2.9	1.087	1.156	0.047	4.1	0.067	6.0	0.071	6.4	0.092	0.99	0.167
NO_3_^−^	0.994	1.123	0.129	12.2	0.906	1.067	0.144	13.5	0.060	5.7	0.045	4.2	0.172	0.98	<0.001
NH_4_^+^	0.295	0.346	0.051	15.9	0.290	0.345	0.057	16.5	0.019	5.9	0.018	5.7	0.073	0.88	<0.001
H^+^	0.018	0.020	0.002	8.1	0.018	0.019	0.001	5.9	0.002	11.2	0.003	13.4	0.004	0.97	0.049
Ca^2+^	0.105	0.120	0.015	13.2	0.102	0.115	0.019	16.9	0.021	19.0	0.028	26.2	0.035	0.61	0.055
Cl^−^	0.061	0.067	0.006	8.9	0.051	0.065	0.009	13.9	0.013	19.9	0.011	19.0	0.015	0.80	0.192
K^+^	0.016	0.019	0.002	13.4	0.015	0.019	0.003	16.4	0.003	18.0	0.004	21.4	0.005	0.71	0.044
Mg^2+^	0.013	0.016	0.003	17.6	0.013	0.015	0.003	16.4	0.002	12.0	0.002	12.6	0.004	0.79	0.001
Na^+^	0.029	0.032	0.003	8.2	0.025	0.031	0.006	19.0	0.008	24.9	0.008	27.4	0.008	0.82	0.323

Depth	mm	mm	mm	%	mm	mm	mm	%	mm	%	mm	%	%		
Ppt	24.8	27.1	2.4	9.1	24.0	26.7	2.3	8.7	1.0	3.7	0.7	2.6	2.9	0.98	<0.001

**Table 8:** Statistics for inter-comparisons of precipitation-weighted annual mean concentrations and annual mean weekly precipitation depths from co-located National Atmospheric Deposition Program (NADP) and Canadian Air and Precipitation Monitoring Network CAPMoN) sites at Frelighsburg, Quebec, Canada, during the period 2012–2019. *p*-values shown are derived with *t*-test [units are in milligrams per liter (mg L^−1^), percent (%), and millimeters (mm), or unitless (Pearson’s *r*, *p*-value), as indicated. *Diff.*, difference; *SD*, standard deviation from mean; *RSD*, percent standard deviation relative to mean of NADP and CAPMoN median values; *MMAD*, modified median absolute deviation between NADP and CAPMoN values; *CoV*, non-parametric coefficient of variation; *P90*, 90^th^ percentile value; Pearson’s *r*, coefficient for correlation of NADP and CAPMoN values; *p*-values are for *t*-test whereby the probability of a null hypothesis is true. The null hypothesis here is that there is no difference between the means (or medians) of CAPMoN and NADP weekly concentrations; *SO*_*4*_^*2−*^, sulfate; *NO*_*3*_^*−*^, nitrate; *NH*_*4*_^*+*^, ammonium; *H*^*+*^, hydrogen ion; *Ca*^*2+*^, calcium; *Cl*^*−*^, chloride; *K*^*+*^, potassium; *Mg*^*2+*^, magnesium; *Na*^*+*^, sodium. Data obtained from NADP, Wisconsin State Laboratory of Hygiene at https://nadp.slh.wisc.edu/networks/national-trends-network and Environment and Climate Change Canada at https://www.canada.ca/en/environment-climate-change/services/air-pollution/monitoring-networks-data/canadian-air-precipitation.html, last accessed August 2023]

	Mean of NADP	Mean of CAPMoN	Diff. of mean	Relative diff. of mean	Median of NADP	Median of CAPMoN	Diff. of median	Relative diff. of median	SD	RSD	MMAD	CoV	P90	Pearson’s *r*	*p*-value
Ion	mg L^−1^	mg L^−1^	mg L^−1^	%	mg L^−1^	mg L^−1^	mg L^−1^	%	mg L^−1^	%	mg L^−1^	%	mg L^−1^		
SO_4_^2−^	0.582	0.553	−0.028	−5.0	0.598	0.556	−0.022	−3.9	0.067	12.0	0.055	9.7	0.046	0.93	0.212
NO_3_^−^	0.862	0.808	−0.054	−6.5	0.830	0.823	−0.045	−5.5	0.141	17.0	0.086	10.3	0.116	0.53	0.254
NH_4_^+^	0.353	0.335	−0.018	−5.2	0.345	0.329	−0.016	−4.8	0.042	12.0	0.034	9.9	0.027	0.72	0.209
H^+^	0.007	0.009	0.002	23.3	0.007	0.009	0.002	23.3	0.002	24.0	0.002	22.0	0.003	0.67	0.013
Ca^2+^	0.128	0.116	−0.012	−9.7	0.123	0.111	−0.004	−3.8	0.035	28.9	0.033	28.5	0.021	0.32	0.318
Cl^−^	0.076	0.064	−0.011	−16.0	0.065	0.061	−0.006	−10.4	0.028	40.6	0.011	17.2	0.011	0.25	0.244
K^+^	0.024	0.020	−0.004	−18.0	0.021	0.017	−0.002	−14.2	0.008	35.9	0.004	19.5	0.002	0.77	0.148
Mg^2+^	0.017	0.016	<0.001	−3.0	0.015	0.015	<0.001	−1.3	0.003	20.6	0.003	19.7	0.002	0.42	0.654
Na^+^	0.042	0.035	−0.007	−18.6	0.036	0.034	−0.003	−9.1	0.018	46.6	0.008	23.0	0.007	0.26	0.239

Depth	mm	mm	mm	%	mm	mm	mm	%	mm	%	mm	%	%		
Ppt	25.5	26.4	0.9	3.6	24.2	24.6	0.9	3.6	2.6	10.0	1.2	4.8	2.9	0.75	0.286

### Annual and seasonal deposition from CAPMoN and NADP

#### Penn State site, PA15/PEN

Over the study period of 1986–2019, estimated annual deposition fluxes were higher for CAPMoN as compared to NADP for SO_4_^2−^ (5.1%), NO_3_^−^ (14.9%), NH_4_^+^ (17.2%), and H^+^ (8.8%) (Table S.4a). The correlations between CAPMoN and NADP annual deposition estimates were very high (*R* >= 0.95) for SO_4_^2−^, NO_3_^−^, NH_4_^+^, and H^+^. For base cations and Cl^−^, annual depositions estimated from CAPMoN data were 12.4–17.1% higher than from NADP data. The corresponding correlations were high for Ca^2+^ and Cl^−^ (*r* = 0.96), relatively high for Mg^2+^ (*r* = 0.91), and moderate for Na^+^ and K^+^ (*r* = 0.73 and 0.51, respectively). As expected, since the deposition depends on both the precipitation-weighted mean annual concentrations and the precipitation depth, both of which had positive median biases (Tables 5 and 6), the biases in deposition fluxes were also positive. During the warm season, the differences in seasonal deposition were small with relative biases less than 10% for all ions except for K^+^ (10.4%) and NH_4_^+^ (14.4%). During the cold season, the relative biases of the annual deposition were 8.0% for SO_4_^2−^, 14.0% for H^+^, and 16.7–30.8% for other ions. During 2005–2019, the relative biases were 8.4% for SO_4_^2−^, and 14.0–22.3% for other ions. The means of the relative biases of all ions during the warm season (10.2%) were smaller than during the cold season (21.0%) (Table S.3).

#### Frelighsburg, CAN5/FRE

The annual depositions estimated from CAPMoN data were higher than from NADP data during 2002–2011 for all ions with a range of 11.1 to 23.0% (Table S.4g). The warm and cold seasonal depositions estimated from CAPMoN data were also higher than from NADP data for all ions, with mean values of 14.1% (warm) and 24.7% (cold). The correlation coefficients ranged from *r*=0.87 to 0.99 during the warm season, and *r*=0.65 to 0.94 during the cold season. For 2012–2019, annual deposition estimates from CAPMoN were lower than from NADP for all ions except for H^+^, with relative biases ranging from −2.7% for NH_4_^+^ to −15.4% for Ca^2+^. Excluding H^+^ and K^+^, the seasonal depositions from CAPMoN were higher than NADP by approximately 2.9% on average for all ions during the warm season, and lower than NADP by 13.5% during the cold season. The estimated annual deposition of H^+^ from CAPMoN was 29.8% higher than from NADP, with positive bias indicated for both warm (46.8%) and cold (13.9%) seasons. Strong correlation of annual depositions were indicated for all ions during the warm season (*r*= 0.96–1.0), except for slightly weaker for K^+^ (*r*=0.84). Overall during 2012–2019, except for H^+^ and K^+^, the agreement of annual deposition estimates from CAPMoN and NADP were very good during the warm season, but there were negative biases during the cold season.

### CAPMoN daily and weekly measurements during 1999–2001 and 2016–2017

#### 1999–2001

From the spring of 1999 to the spring of 2001, a field study was carried out at Egbert, Ontario, Canada, by the CAPMoN network. Four identical CAPMoN precipitation collectors were deployed at the site to measure wet deposition of the common CAPMoN and NADP ions. Two samplers were operated to collect daily samples (coded as EG1 and EG2) and the other two collected weekly samples (coded as EW1 and EW2). The main objective of the study was to assess the comparability of the daily and weekly sampling. For this analysis, weekly precipitation-weighted mean concentrations were calculated from the daily sample data (hereinafter referred as “weekly composite,” coded as EG1 and EG2) for comparison to the corresponding weekly sample concentrations (coded as EW1 and EW2), analogous to the NADP comparison methodology. From the weekly concentrations, precipitation-weighted mean concentration was calculated for the entire study period 1999–2001, using the equation similar to Eq. 5. As an example, scatter plots of weekly concentrations from EG1 and EW1 are shown in Fig. S.1 and show that weekly concentrations from the weekly sampling and the daily sampling were generally comparable, especially for SO_4_^2−^, NO_3_^−^, and NH4^+^. Relatively larger differences occurred for higher weekly concentrations for all ions. These higher weekly concentrations were usually associated with relatively lower weekly precipitation amounts (e.g., on average, weekly precipitation amount of 3.8 mm/week vs 20.4 mm/week for weekly concentrations of SO_4_^2−^ greater vs less than 75 μeq L^−1^). Table 9 shows that, except for H^+^, the weekly concentrations from the weekly measurements were higher than those from the daily measurements. Mean daily-minus-weekly concentration differences between the two sampling protocols were approximately −0.2 μeq L^−1^ for Mg^2+^, K^+^, Na^+^, Cl^−^; −0.4 to −0.8 μeq L^−1^ for NO_3_^−^, Ca^2+^, and NH_4_^+^; and −1.0 μeq L^−1^ for SO_4_^2−^. The relative differences in precipitation-weighted concentration ranged from −1.2 to −3.1% for SO_4_^2−^, NO_3_^−^, and NH_4_^+^; −4.2 to −7.2% for Ca^2+^, Mg^2+^, Na^+^, and Cl^−^; and −24.4% for K^+^. For H^+^, the weekly measurements underestimated the precipitation-weighted mean concentration by 2.5 μeq L^−1^, or 8.2%. The measurements from EW1 had relatively large biases, compared to the measurements from EW2, EG1, and EG2. If we only look at the relative differences between EW2 and EG1 or EG2, the weekly measurements overestimate SO_4_^2−^, NO_3_^−^, and NH_4_^+^ by 1–2%; Ca^2+^, Mg^2+^, Na^+^, and Cl^−^ by 3–5%; and K^+^ by 14%.

**Table 9:** Precipitation-weighted mean concentrations and relative differences of daily-vs-daily, weekly-vs-weekly, and daily-vs-weekly measurements at Egbert, Ontario, Canada, for 1999–2001 [two daily samples (EG1 and EG2) and two weekly samples (EW1 and EW2) were deployed at the measurement site. EG1&2-minus-EW1 is calculated as the mean of EG1-minus-EW1 and EG2-minus-EW1, and it is similar for EG1&2-minus-EW2; daily-minus-weekly is calculated as the mean of EG1-minus-EW1, EG2-minus-EW1, EG1-minus-EW2, and EG2-minus-EW2. The calculation of the relative difference is based on Eq. 4 in the text. Data obtained from NADP, Wisconsin State Laboratory of Hygiene at https://nadp.slh.wisc.edu/networks/national-trends-network and Environment and Climate Change Canada at https://www.canada.ca/en/environment-climate-change/services/air-pollution/monitoring-networks-data/canadian-air-precipitation.html, last accessed August 2023]

	Precipitation-weighted mean concentration				Relative difference								
Ion	EG1	EG2	EW1	EW2	EG2 minus EG1	EW2 minus EW1	EG1 minus EW1	EG1 minus EW2	EG2 minus EW1	EG2 minus EW2	EG1&2 minus EW1	EG1&2 minus EW2	Daily minus Weekly
	μeq L^−1^	μeq L^−1^	μeq L^−1^	μeq L^−1^	%	%	%	%	%	%	%	%	%
SO_4_^2−^	35.7	35.5	36.7	36.4	−0.6	−0.9	−2.8	−1.9	−3.4	−2.5	−3.1	−2.2	−2.6
NO_3_^−^	30.1	30.0	30.4	30.4	−0.4	−0.1	−1.0	−1.0	−1.4	−1.3	−1.2	−1.1	−1.2
NH_4_^+^	23.5	23.6	24.8	23.8	0.4	−3.7	−5.1	−1.4	−4.7	−1.0	−4.9	−1.2	−3.1
H^+^	31.7	30.9	28.5	29.1	−2.6	2.1	10.5	8.5	8.0	5.9	9.2	7.2	8.2
Ca^2+^	10.6	10.6	11.3	11.0	−0.3	−2.9	−6.4	−3.5	−6.8	−3.9	−6.6	−3.7	−5.1
Mg^2+^	2.9	2.8	3.1	3.0	−2.0	−2.3	−6.7	−4.5	−8.7	−6.5	−7.7	−5.5	−6.6
K^+^	0.6	0.6	0.9	0.7	−4.5	−21.5	−32.9	−11.6	−37.2	−16.0	−35.1	−13.8	−24.4
Na^+^	2.2	2.1	2.4	2.2	−1.5	−8.3	−10.7	−2.3	−12.1	−3.8	−11.4	−3.1	−7.2
Cl^−^	3.2	3.2	3.4	3.4	−0.1	0.0	−4.1	−4.1	−4.2	−4.2	−4.2	−4.2	−4.2

Table S.5 shows the inter-comparisons of median values of the weekly concentrations, which eliminate the effects on the comparisons from extreme values. For EG1-vs-EG2 comparisons, only Ca^2+^ had a relative difference greater than 1%. For EW1-vs-EW2 comparisons, only K^+^ had a relative difference greater than 1%. Using median value as a metrics, the weekly measurements showed 1–3% higher SO_4_^2−^, NO_3_^−^, and NH_4_^+^ concentrations; Ca^2+^, Mg^2+^, Na^+^, and Cl^−^ by 1–5%; and K^+^ by 6% compared to weekly composite values derived with daily measurements. Only H^+^ was higher in the daily composites, by 5%. Daily-vs-daily and weekly-vs-weekly comparisons are usually mentioned as “within-method” comparison, while daily-vs-weekly comparisons are named as “between-method” comparison. The results in Table S.4 show that the biases of daily-vs-weekly comparisons are systematic and they are higher than the biases of the “within-method” comparisons.

#### 2016–2017

To aid the CAPMoN network in the transition of CAPMoN measurements of wet deposition at some sites from a daily basis to a weekly basis, parallel measurements with daily and weekly sampling frequencies were carried out by the CAPMoN network during 2016–2017 at Egbert and Algoma sites in Ontario, Canada, and at the Jackson site in Nova Scotia, Canada. Two types of sample collectors, C300 and D400, were deployed at Egbert. Only C300 samplers were deployed at Algoma and Jackson. As shown in the scatter plots (Fig. S.2), weekly concentrations from daily and weekly samples were generally comparable for this inter-comparison, except for H^+^. Very good agreements of weekly concentration were found for SO_4_^2−^, NO_3_^−^, and NH_4_^+^ between daily and weekly sampling. Tables 10 and 11 show that the impact on transitioning from daily to weekly measurements resulted in a decrease in the precipitation-weighted mean concentration for H^+^, and an increase in the precipitation-weighted mean concentrations for other ions. The effect on the precipitation-weighted mean concentration of H^+^ was −1.3 μeq L^−1^, or −30%, on average, while the effect on SO_4_^2−^, NO_3_^−^, and NH_4_^+^ was less than 0.3 μeq L^−1^, or 3%. The effect on Ca^2+^ and Mg^2+^ ranged 0.3–0.6 μeq L^−1^, or 5–10%; the impact on Na^+^ and Cl^−^ was approximately 0.2 μeq L^−1^, or 2%, and the impact on K^+^ was smallest in absolute value, being 0.1 μeq L^−1^, but 10.3% in relative difference as the mean concentration of K^+^ was the lowest among all ions.

**Table 10:** Weekly-minus-daily precipitation-weighted mean concentration differences for precipitation samples collected at Egbert, Ontario; Jackson, Nova Scotia; and Algoma, Ontario, Canada, during 2016–2017. Mean concentration differences are in unit of microequivalents per liter (μeq L^−1^). Percentages difference is defined as 100 × 2 × mean difference / (daily precipitation-weighted mean concentration + weekly precipitation-weighted mean concentration) [two types of precipitation collectors (C300 and D400) were deployed at Egbert. SO_4_^2−^, sulfate; NO_3_^−^, nitrate; NH_4_^+^, ammonium; H^+^, hydrogen ion; Ca^2+^, calcium; Cl^−^, chloride; K^+^, potassium; Mg^2+^, magnesium; Na^+^, sodium; Cations, sum of Ca^2+^ + Mg^2+^ + Na^+^ + K^+^ + NH_4_^+^ + H^+^; Anions, sum of SO_4_^2−^ + NO_3_^−^ + Cl^−^]

	Site location, collector type (units)				
Ion	Egbert, C300 (μeq L^−1^)	Egbert, D400 (μeq L^−1^)	Jackson, C300 (μeq L^−1^)	Algoma, C300 (μeq L^−1^)	Average (μeq L^−1^)
SO_4_^2−^	0.31	0.37	0.42	−0.14	0.24
NO_3_^−^	0.39	0.39	0.28	0.00	0.27
NH_4_^+^	−0.04	0.40	0.20	−0.02	0.14
H^+^	−1.3	−1.45	−1.03	−1.22	−1.25
Ca^2+^	0.77	1.14	0.08	0.30	0.57
Mg^2+^	0.39	0.54	0.12	0.23	0.32
K^+^	0.08	0.18	0.04	−0.01	0.07
Na^+^	0.11	0.09	0.55	−0.05	0.18
Cl^−^	0.12	0.08	0.73	−0.06	0.22
Cations	0.44	0.88	0.03	−0.76	0.15
Anions	0.81	0.84	1.44	−0.20	0.72

**Table 11:** Weekly-minus-daily precipitation-weighted percentage differences for precipitation samples collected at Egbert, Ontario; Jackson, Nova Scotia; and Algoma, Ontario, Canada, during 2016–2017. Mean concentration differences are in unit of microequivalents per liter (μeq L^−1^). Percentages difference is defined as 100 × 2 × mean difference / (daily precipitation-weighted mean concentration + weekly precipitation-weighted mean concentration) [two types of precipitation collectors (C300 and D400) were deployed at Egbert. *SO*_*4*_^*2−*^, sulfate; *NO*_*3*_^*−*^, nitrate; *NH*_*4*_^*+*^, ammonium; *H*^*+*^, hydrogen ion; *Ca*^*2+*^, calcium; *Cl*^*−*^, chloride; *K*^*+*^, potassium; *Mg*^*2+*^, magnesium; *Na*^*+*^, sodium; Cations, sum of Ca^2+^ + Mg^2+^ + Na^+^ + K^+^ + NH_4_^+^ + H^+^; Anions, sum of SO_4_^2−^ + NO_3_^−^ + Cl^−^]

	Site location, collector type				
Ion	Egbert, C300 %	Egbert, D400 %	Jackson, C300 %	Algoma, C300 %	Average %
SO_4_^2−^	3.1	3.5	5.3	−1.1	2.7
NO_3_^−^	2.8	2.7	5.0	< 0.1	2.6
NH_4_^+^	−0.2	1.6	3.1	−0.1	1.1
H^+^	−36.3	−45.3	−14.4	−22.9	−29.7
Ca^2+^	6.3	8.3	3.1	3.2	5.2
Mg^2+^	11.4	13.9	3.0	8.7	9.3
K^+^	13.2	23.8	6.2	−2.0	10.3
Na^+^	4.3	3.5	3.5	−2.7	2.2
Cl^−^	4.4	3.0	4.1	−3.3	2.1
Cation	1.2	2.3	0.1	−2.1	0.4
Anion	3.1	3.1	4.6	−0.7	2.5

Considering only the impacts of sample collection frequency, the inter-comparison results from 1999–2001 and 2016–2017 show that the precipitation-weighted mean concentrations from daily measurements tend to be lower than the weekly measurements by 1–3% for SO_4_^2−^, NO_3_^−^, and NH_4_^+^; 2–9% for Ca^2+^, Mg^2+^, Na^+^, and Cl^−^; 10–24% for K^+^; and 8–30% higher for H^+^. These results suggest that, apart from H^+^, the overall higher concentrations from CAPMoN at the Penn State and Frelighsburg (during 2002–2011) sites were not due to the difference in sampling frequency between the two networks, because CAPMoN weekly composite measurements from daily data were lower than the CAPMoN weekly measurements except for H^+^. Inter-comparisons of wet deposition measurements with different sampling periods, namely, weekly-vs-event, have been conducted in previous studies (e.g., De Pena et al., 1985; Sisterson et al., 1985). Sample collection for events started and ended with each precipitation event, a somewhat different protocol than regular daily collection. The CAPMON daily-vs-weekly inter-comparison results are not consistent with the previous study by De Pena et al. (1985), which found that the weekly samples yielded lower concentrations for all ions than event samples. The significant underestimation of NH_4_^+^ concentrations by weekly sampling reported by Sisterson et al. (1985) was not observed in this study.

## Discussion

### Causes of underestimation of wet concentration of H^+^ in daily-vs-weekly inter-comparison

**Fig. 4:**
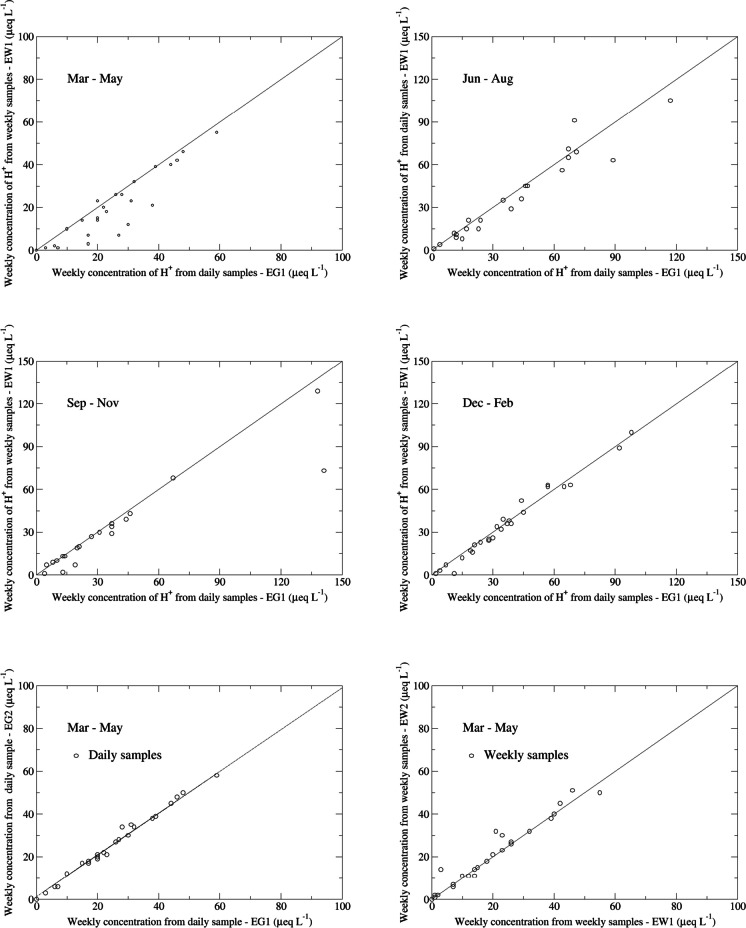
Weekly wet concentrations of H^+^ from daily and weekly samples collected during 1999–2001 at Egbert, Ontario, by the CAPMoN network for different seasons. EG1 is daily sampling and EW1 and EW2 are weekly sampling. Wet concentrations greater than 150 μeq L^−1^, which are less than 2.5% of the total weekly samples, are not shown in the figures

Significantly lower concentrations of H^+^ in weekly samples were shown in the daily-vs-weekly inter-comparisons of the CAPMoN measurements during 1999–2001 and 2016–2017, and were also reported by De Pena et al. (1985) and Sisterson et al. (1985). It has been suggested that biological conversion might have played a role in lowering the acidity in weekly samples (Sirois et al., 2000; Sisterson et al., 1985). To test the hypothesis that lower concentrations of H^+^ in weekly samples were caused by biological conversion, at least partially, we analyzed the daily-vs-weekly measurements for winter months (December–February) and summer months (June–August) of the 1999–2001 and 2016–2017 data. The relative biases, expressed as daily-minus-weekly, of the precipitation-weighted mean concentration of H^+^ were reduced from 8.2% for the summer season to 1.1% for the winter season for 1999–2001 data. For the 2016–2017 data, the relative biases were reduced from 61% (for C300 samplers) and 79% (for D400 samplers) for the summer season to 23% (C300) and 21% (D400) at the Egbert site. For the Jackson site, it was reduced from 30 to 7.9%. No seasonal analysis was performed for the Algoma site due to limited data. Examples of scatter plots of weekly concentrations of H^+^ from daily samples vs. weekly samples are shown in Fig. 4 for different seasons for the 1999–2001 data at Egbert. The largest difference between daily samples and weekly samples occurred in the spring season (March–May, 19.4%), and the difference during the winter season (December–February, 1.1%) was very small. The large relative bias for the spring season was not due to quality assurance (QA)/quality control (QC), as the agreement of daily-vs-daily measurements and weekly-vs-weekly measurements for the spring season (March–May) was good (Fig. 4), with the relative differences of 4.7% and 11.5% for daily-vs-daily and weekly-vs-weekly measurements, respectively. The small relative difference between weekly and daily samples during the winter season is assumed to be because (1) low ambient temperature inhibited biological activities in weekly samples and (2) organic acids emitted from trees and vegetation were significantly lower during the cold season (Feng et al., 2021; Song & Gao, 2009), causing biological conversion of organic acids to be less significant for December–February. Feng et al. (2021) showed that organic acids made more contribution to the free H^+^ in precipitation samples when the overall acidity of precipitation decreased during the past three decades in eastern North America, which could account for larger discrepancies in the 2016-17 studies than in 1999-2001.

### Causes of between-network biases

A number of potential causes for the observed between-network biases were proposed in Sirois et al. (2000): (1) CAPMoN sensors were more sensitive to the onset of precipitation and collected more high-concentration precipitation; (2) different sampling frequency, namely daily vs. weekly, might have different effects on evaporation, particle dissolution, and biological uptake/decomposition. The laboratory differences and background contamination were also examined by Sirois et al. (2000), but were excluded as the major contributors to the observed between-network biases. They suggested that biological uptake/conversion might be the most likely dominant causes for the between-network biases of NO_3_^−^, NH_4_^+^, and H^+^.

Wetherbee et al. (2010) examined contamination due to field exposure, sample handling, shipping, and analysis. They concluded that the contamination was higher in NADP samples than CAPMoN samples. Therefore, contamination could not account for the observed positive between-network biases when the ACM collectors were deployed at the Penn State and Frelighsburg sites. Wetherbee et al. (2010) also discounted the difference in analytical laboratories of the NADP and CAPMoN networks as a potential source for the observed between-network biases.

For the current inter-comparison study, we have extra measurements not available in the studies by Sirois et al. (2000) and Wetherbee et al. (2010), namely the daily-vs-weekly inter-comparisons of CAPMoN measurements and the CAPMoN-vs-NADP inter-comparison at Frelighsburg for 2012–2019 for which the NCON was deployed as a NADP sampler. The daily-vs-weekly comparisons of CAPMoN measurements show that weekly measurements had higher concentrations for all ions except for H^+^, possibly due to a small amount of evaporation over the sampling week. However, the impact was only 1–3% for SO_4_^2−^, NO_3_^−^, and NH_4_^+^, and it was in the opposite direction of the between-network biases. Additionally, Wetherbee and Rhodes (2013) observed minimal (median <5%) weekly sample evaporation of ACM samples dwelling for 1 week during summer in Colorado, USA. Therefore, we conclude that sampling frequency cannot account for the positive between-network biases when the ACM collectors were deployed at Penn State and Frelighsburg. When the NCON collector was deployed at Frelighsburg during 2012–2019, the between-network biases flipped from positive to negative for all ions except for H^+^, consistent with the pattern seen in the daily-vs-weekly CAPMoN studies. This result suggests that the type of collector is likely the main cause of the observed biases that have been documented for CAPMoN and NADP over the past 33 years.

### Impact of sample depth on between-network biases

**Fig. 5:**
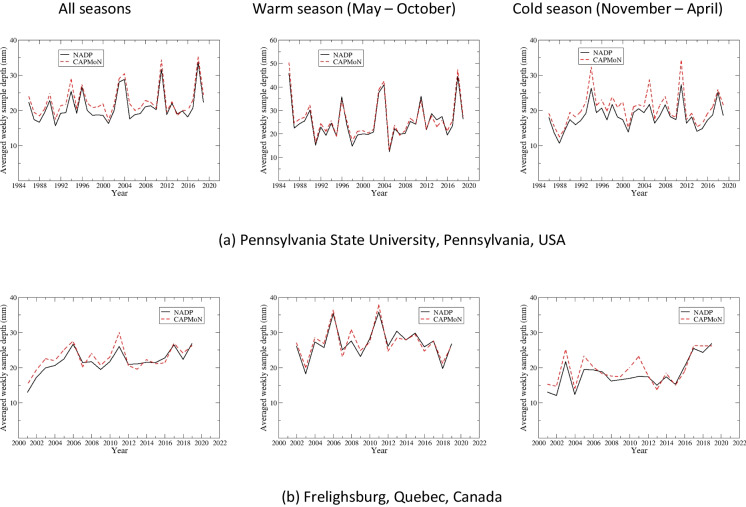
Annually averaged and seasonally averaged weekly precipitation-sample depths calculated from volumes of samples collected by the National Atmospheric Deposition Program (NADP) and Canadian Air and Precipitation Monitoring Network (CAPMoN) sites co-located at **a** Pennsylvania State University, PA, USA, and **b** Frelighsburg, Quebec, Canada, 1986–2019

Time series of the annual and the seasonal mean weekly sample depths at the Penn State and Frelighsburg sites are shown in Fig. 5. At Penn State, the weekly sample depths from CAPMoN were higher than those from NADP for both warm and cold seasons. Tables 12 and 13 show that for the whole study period of 1986–2019, the sample depths from CAPMoN were higher annually (9%) and for the warm (7%) and cold (10%) seasons. Similar to the Penn State site, the sample depths from CAPMoN were higher than NADP for 2002–2011 at Frelighsburg both annually (8%), and for the warm (6%) and cold (13%) seasons. However, when the sample collector at Frelighsburg changed from the ACM to the NCON, the sample depths from CAPMoN and NADP became comparable. The medians of the ratio of the weekly sample depth from CAPMoN to NADP were 1.0 during the warm and cold seasons. Figure 5 shows that the agreement of sample depths from CAPMoN and NADP at Frelighsburg improved during 2012–2019.

**Table 12:**
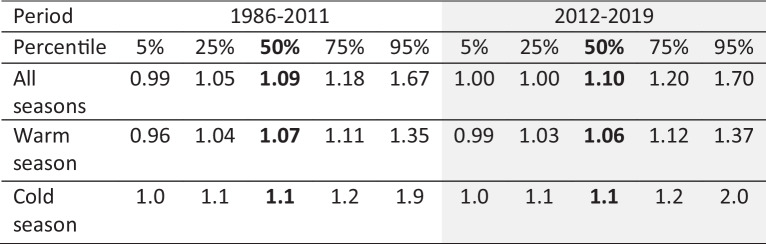
Percentiles of ratios of weekly sample depth analyzed seasonally for National Atmospheric Deposition Program (NADP) and Canadian Air and Precipitation Monitoring Network (CAPMoN) precipitation samples obtained from co-located collectors for selected periods at Pennsylvania State University, PA, USA. The ratio is defined as the sample depth of CAPMoN to NADP [median (50^th^ percentile) values are in bold. Warm season: May–October; cold season: November–April; shading denotes similar period 2012–2019 for both locations. Data obtained from NADP, Wisconsin State Laboratory of Hygiene at https://nadp.slh.wisc.edu/networks/national-trends-network and Environment and Climate Change Canada at https://www.canada.ca/en/environment-climate-change/services/air-pollution/monitoring-networks-data/canadian-air-precipitation.html, last accessed August 2023]

**Table 13:**
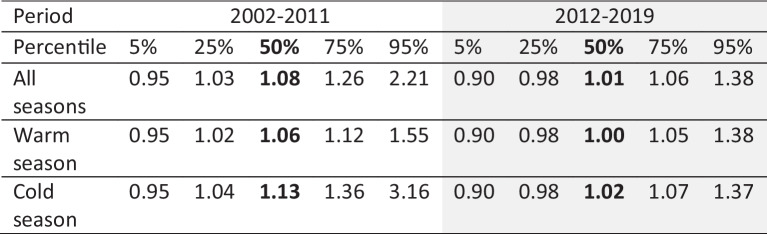
Percentiles of ratios of weekly sample depth analyzed seasonally for National Atmospheric Deposition Program (NADP) and Canadian Air and Precipitation Monitoring Network (CAPMoN) precipitation samples obtained from co-located collectors for selected periods at Frelighsburg, Quebec, Canada. The ratio is defined as the sample depth of CAPMoN to NADP [median (50^th^ percentile) values are in bold. Warm season: May–October; cold season: November–April; shading denotes similar period 2012–2019 for both locations. Data obtained from NADP, Wisconsin State Laboratory of Hygiene at https://nadp.slh.wisc.edu/networks/national-trends-network and Environment and Climate Change Canada at https://www.canada.ca/en/environment-climate-change/services/air-pollution/monitoring-networks-data/canadian-air-precipitation.html, last accessed August 2023]

The change of the comparability of sample depth between CAPMoN and NADP was consistent with the change of the between-network biases from positive to negative for most ions except for H^+^ and K^+^. These results suggest that the positive between-network biases at Penn State and Frelighsburg (2001–2011) could be mainly due to the differences in sample collector performance for the two networks.

Differences in sample depth between CAPMoN and NADP can be due to the sensitivity of the sensors, which open and close the lids of the sample collectors. The CAPMoN precipitation sensor is more sensitive than that of the ACM (Landis & Keeler, 1997; Sirois et al., 2000; Wetherbee et al., 2010), and opens earlier. As concentrations of ions are usually higher at the beginning of a precipitation event, it is expected that CAPMoN’s collectors collect more samples with higher concentration of ions than NADP’s ACM collector. This partially explains why the concentrations of all ions, except for H^+^, measured by CAPMoN were higher than those from NADP when the ACM was deployed at the Penn State and Frelighsburg sites. With the NCON being deployed at Frelighsburg during 2012–2019, the sample depths from NADP and CAPMoN became comparable, and the overall between-network biases turned negative (except for H^+^ and K^+^). The between-network bias for H^+^ continued to be positive at Frelighsburg during 2012–2019. This is consistent with the results of the CAPMoN daily-vs-weekly inter-comparisons, which showed that the concentrations of H^+^ in weekly samples were substantially lower than daily samples. Biological uptakes/conversion and particle dissolution might have reduced the concentrations of H^+^ in weekly samples (Sirois et al., 2000; Sisterson et al., 1985).

### Implications for data usage

Data quality objectives (DQOs) for inter-network biases are laid out in the World Meteorological Organization Global Atmosphere Watch Manual for the Precipitation Chemistry Programme (WMO, 2004). The MMAD values reported for all ions in the current analysis, for every site and time period examined, fall within these objectives. It should be noted that these objectives were based on the MMAD values from Sirois et al. (2000). Given the significant decreases in SO_4_^2−^ and NO_3_^−^ concentrations in North America since that time, it may be advisable to modify these DQOs, which are currently 0.42 and 0.36 mg L^−1^, respectively, to reflect the current levels of sulfate and nitrate deposition around the globe.

Wet deposition data from NADP and CAPMoN have been jointly used for evaluating global and regional models (Tan et al., 2018), usually with a focus on sulfur and nitrogen deposition. For these users, a historic systematic difference in annual deposition between the networks of approximately 5–10% for sulfate, 15% for nitrate, and 20% for ammonium should be noted, with the caveat that changes in the NADP sample collector and rain gauge since 2010 at certain NADP sites (Wetherbee, 2017) have reduced these differences to <5%. Seasonal differences can be larger and are included for applications of higher-frequency analyses. Recent and ongoing changes, such as the switch to bag sampling in NADP and the conversion of some CAPMoN sites to weekly sampling, will need to be addressed through continued inter-comparison measurements.

## Summary

In this study, we compared the weekly concentrations of nine major ions measured by NADP and CAPMoN at two sites located in the Penn State, USA, and Frelighsburg, Canada, for the periods of 1986–2019 and 2001–2019 respectively. The major findings from this inter-comparison study are summarized as follows:

1. At the Penn State site, for the whole study period 1986–2019, CAPMoN was higher than NADP for all ions, in terms of weekly concentration, precipitation-weighted annual mean concentration, and annual deposition. The precipitation-weighted annual mean concentrations were higher for SO_4_^2−^ (2%), NO_3_^−^ (12%), NH_4_^+^ (16%), H^+^ (6%), and base cations and Cl^−^ (11–15%). For annual deposition, CAPMoN was 5–17% higher for all ions, with the best agreement for SO_4_^2−^.
2. At Frelighsburg, NADP changed the sample collector from the ACM to the NCON in October 2011. For 2002–2011, the relative differences at Frelighsburg were similar to those at the Penn State site, with relative differences varying from 4 to 25% for all ions. In contrast, for 2012–2019, the precipitation-weighted annual mean concentrations were 1–14% lower than NADP, except for H^+^, which was 23% higher; the NADP-measured precipitation amount was 3.6% higher. Correspondingly, the annual deposition from CAPMoN was 3–15% lower than NADP, except that the H^+^ deposition was 30% higher. This study further confirms that the implementation of a new type of sample collector introduced systematic and notable changes in measured concentrations and deposition within the NADP network, as indicated in the previous study by Wetherbee (2017).
3. The inter-comparison results at Penn State and Frelighsburg (for 2002–2011) show that the deposition of nitrogen measured by CAPMoN was 15–18% (NO_3_^−^) and 14–23% (NH_4_^+^) higher than NADP when precipitation samples were collected by the ACM collector. However, when the NCON collector was deployed at the Frelighsburg site, the annual deposition of nitrogen by CAPMoN was 5% and 3% lower than NADP for NO_3_^−^ and NH_4_^+^, respectively. This study shows that between-network differences and within-network differences (e.g., the change of samplers) create biases in the data that need to be considered when assessing the impact on ecosystems from atmospheric deposition (e.g., from nitrogen deposition).
4. The comparisons of daily-vs-weekly measurements conducted by the CAPMoN network during 1999–2001 and 2016–2017 show that the precipitation-weighted mean concentrations by weekly measurements were higher those from daily measurements by 1–3% for SO_4_^2−^, NO_3_^−^, and NH_4_^+^; 3–9% for Ca^2+^, Mg^2+^, Na^+^, Cl^−^; 10–24% for K^+^; and lower for H^+^ by 8–30%. The direction of the biases was opposite to that of the inter-network biases seen at Penn State and Frelighsburg; thus, the inter-network biases were not due to sampling frequency.
5. This study highlights the importance of the performance of precipitation sample collectors, and attributes lower efficiency in capturing onset precipitation by the ACM collectors as one of the causes of the positive relative biases at Penn State and Frelighsburg (2002–2011).

### Supplementary information

ESM 1 (DOCX 312 kb)

## Data Availability

The CAPMoN and NADP data used in this study are available for download from https://www.canada.ca/en/environment-climate-change/services/air-pollution/monitoring-networks-data/canadian-air-precipitation.html and https://nadp.slh.wisc.edu/networks/national-trends-network, respectively. CAPMoN daily-vs-weekly measurement data are available by contacting ec.natchem.ec@canada.ca.
