# Supplementary material for: Inter-comparison of measurements of inorganic chemical components in precipitation from NADP and CAPMoN at collocated sites in the USA and Canada during 1986–2019
Source: Environ Monit Assess. 2023 Oct 18;195(11):1333. doi: 10.1007/s10661-023-11771-z (PMC10584717; doi:10.1007/s10661-023-11771-z)
Supplement: Supplementary file 1 — (DOCX 312 kb) [file 10661_2023_11771_MOESM1_ESM.docx]

Table S.1 Linear regression coefficients of precipitation-weighted monthly mean concentrations (mg L^-1^) of CAPMoN-*vs*-NADP for: (a) the Penn State University site, 1986-2019; (b) the Frelighsburg site, 2002-2011; (c) the Frelighsburg site, 2012-2019. a, b, and *r* are slope (unitless), intercept (mg L^-1^), and Pearson correlation coefficient (unitless) of the linear regressions, respectively. Scatter plots of CAPMoN-*vs*-NADP for all seasons are shown in Fig. 1 for the Penn State University site. Data obtained from NADP, Wisconsin State Laboratory of Hygiene at https://nadp.slh.wisc.edu/networks/national-trends-network and Environment and Climate Change Canada at https://www.canada.ca/en/environment-climate-change/services/air-pollution/monitoring-networks-data/canadian-air-precipitation.html, last accessed August 2023.

(a) Penn State University, 1986-2019

| Ion | All seasons | | | Warm season (May – October) | | | Cold season (November – April) | | |
| --- | --- | --- | --- | --- | --- | --- | --- | --- | --- |
|  | a | b  mg L^-1^ | Pearson  *r* | a | b  mg L^-1^ | Pearson  *r* | a | b  mg L^-1^ | Pearson  *r* |
| SO_4_^2-^ | 1.01 | 0.066 | 0.98 | 1.00 | 0.080 | 0.98 | 1.03 | 0.040 | 0.98 |
| NO_3_^-^ | 1.08 | 0.097 | 0.97 | 1.06 | 0.046 | 0.98 | 1.09 | 0.148 | 0.97 |
| NH_4_^+^ | 1.05 | 0.043 | 0.94 | 0.97 | 0.067 | 0.90 | 1.11 | 0.028 | 0.97 |
| H^+^ | 1.09 | 0.001 | 0.98 | 1.08 | 0.001 | 0.98 | 1.12 | 0.001 | 0.97 |
| Ca^2+^ | 1.03 | 0.014 | 0.88 | 0.88 | 0.019 | 0.92 | 1.2 | 0.009 | 0.89 |
| Cl^-^ | 0.94 | 0.026 | 0.86 | 1.03 | 0.006 | 0.95 | 0.86 | 0.048 | 0.81 |
| K^+^ | 0.19 | 0.019 | 0.37 | 0.12 | 0.023 | 0.28 | 0.76 | 0.009 | 0.72 |
| Mg^2+^ | 0.98 | 0.004 | 0.86 | 0.89 | 0.004 | 0.89 | 1.12 | 0.003 | 0.86 |
| Na^+^ | 0.79 | 0.012 | 0.80 | 0.92 | 0.001 | 0.87 | 0.68 | 0.026 | 0.74 |

(b) Frelighsburg, 2002-2011

| Ion | All seasons | | | Warm season (May – October) | | | Cold season (November – April) | | |
| --- | --- | --- | --- | --- | --- | --- | --- | --- | --- |
|  | a | b  mg L^-1^ | Pearson  *r* | a | b  mg L^-1^ | Pearson  *r* | a | b  mg L^-1^ | Pearson  *r* |
| SO_4_^2-^ | 0.90 | 0.146 | 0.99 | 0.9 | 0.144 | 0.99 | 0.85 | 0.188 | 0.93 |
| NO_3_^-^ | 0.84 | 0.312 | 0.93 | 0.71 | 0.311 | 0.98 | 0.85 | 0.437 | 0.90 |
| NH_4_^+^ | 1.13 | 0.016 | 0.98 | 1.17 | 0.002 | 1.00 | 0.90 | 0.081 | 0.88 |
| H^+^ | 0.85 | 0.005 | 0.94 | 0.83 | 0.004 | 0.96 | 0.86 | 0.005 | 0.92 |
| Ca^2+^ | 0.82 | 0.045 | 0.83 | 0.77 | 0.037 | 0.97 | 1.00 | 0.036 | 0.65 |
| Cl^-^ | 0.62 | 0.036 | 0.90 | 0.84 | 0.010 | 0.96 | 0.58 | 0.059 | 0.89 |
| K^+^ | 0.67 | 0.008 | 0.67 | 0.69 | 0.007 | 0.75 | 0.58 | 0.010 | 0.38 |
| Mg^2+^ | 0.79 | 0.006 | 0.88 | 0.88 | 0.004 | 0.97 | 0.64 | 0.008 | 0.71 |
| Na^+^ | 0.53 | 0.021 | 0.83 | 0.87 | 0.004 | 0.96 | 0.48 | 0.039 | 0.81 |

(c) Frelighsburg, 2012-2019

| Ions | All seasons | | | Warm season (May – October) | | | Cold season (November – April) | | |
| --- | --- | --- | --- | --- | --- | --- | --- | --- | --- |
|  | a | b  mg L^-1^ | Pearson  *r* | a | b  mg L^-1^ | Pearson  *r* | a | b  mg L^-1^ | Pearson  *r* |
| SO_4_^2-^ | 0.74 | 0.106 | 0.84 | 1.01 | -0.001 | 0.967 | 0.496 | 0.207 | 0.74 |
| NO_3_^-^ | 0.75 | 0.164 | 0.90 | 1.00 | -0.015 | 0.979 | 0.709 | 0.211 | 0.85 |
| NH_4_^+^ | 1.14 | -0.073 | 0.72 | 1.46 | -0.180 | 0.767 | 0.563 | 0.103 | 0.74 |
| H^+^ | 0.85 | 0.003 | 0.84 | 1.41 | 0.002 | 0.823 | 0.855 | 0.003 | 0.82 |
| Ca^2+^ | 0.54 | 0.043 | 0.80 | 0.96 | 0.004 | 0.854 | 0.505 | 0.039 | 0.82 |
| Cl^-^ | 0.36 | 0.042 | 0.70 | 0.75 | 0.016 | 0.523 | 0.313 | 0.054 | 0.69 |
| K^+^ | 0.62 | 0.017 | 0.13 | 0.56 | 0.032 | 0.095 | 0.062 | 0.013 | 0.21 |
| Mg^2+^ | 0.66 | 0.006 | 0.60 | 0.68 | 0.009 | 0.306 | 0.682 | 0.002 | 0.92 |
| Na^+^ | 0.38 | 0.020 | 0.73 | 0.79 | 0.007 | 0.668 | 0.332 | 0.030 | 0.67 |

Table S.2 Statistics for inter-comparisons of weekly ion concentrations and precipitation (Ppt) depths measured from co-located National Atmospheric Deposition Program (NADP) and Canadian Air and Precipitation Monitoring Network (CAPMoN) sites at Pennsylvania State University, Pennsylvania, USA and Frelighsburg, Quebec, Canada for both warm and cold seasons during selected periods: Penn State University, 1986-2019; Penn State University, 2005-2019; Frelighsburg, 2002-2011; Frelighsburg, 2012-2019. [Units are in milligrams per liter (mg L^-1^), percent (%), and millimeters (mm), or unitless (Pearson *r*, *p-value*), respectively. Diff., difference; ARB, absolute relative bias; SD, standard deviation from mean; RSD, percent standard deviation relative to mean of NADP and CAPMoN median values; MMAD, Modified median absolute deviation between NADP and CAPMoN values; CoV, non-parametric coefficient of variation; P90, 90^th^ percentile value; Pearson R, coefficient for correlation of NADP and CAPMoN values; *p*-value, the probability of a null hypothesis is true. The null hypothesis here is that there is no difference between the means (or medians) of CAPMoN and NADP weekly concentrations. *p*-values shown are derived with Wilcoxon signed-rank test ; SO_4_^2-^, sulfate; NO_3_^-^, nitrate; NH_4_^+^, ammonium; H^+^, hydrogen ion; Ca^2+^, calcium; Cl^-^, chloride; K^+^, potassium; Mg^2+^, magnesium; Na^+^, sodium. Data obtained from NADP, Wisconsin State Laboratory of Hygiene at https://nadp.slh.wisc.edu/networks/national-trends-network and Environment and Climate Change Canada at https://www.canada.ca/en/environment-climate-change/services/air-pollution/monitoring-networks-data/canadian-air-precipitation.html, last accessed August 2023.]

| (a) Penn State University, 1986-2019, warm season | | | | | | | | | | | | | | | | |
| --- | --- | --- | --- | --- | --- | --- | --- | --- | --- | --- | --- | --- | --- | --- | --- | --- |
|  | Mean of NADP | Mean  of CAPMoN | Diff. of mean | Relative diff. of mean | Median of NADP | Median of CAPMoN | Diff. of median | Relative diff. of median | ARB | SD | RSD | MMAD | CoV | P90 | Pearson *r* | *p*-value |
| Ion | mg L^-1^ | mg L^-1^ | mg L^-1^ | % | mg L^-1^ | mg L^-1^ | mg L^-1^ | % | % | mg L^-1^ | % | mg L^-1^ | % | mg L^-1^ |  |  |
| SO_4_^2-^ | 2.856 | 2.936 | 0.080 | 2.8 | 2.350 | 2.492 | 0.047 | 1.9 | 4.5 | 0.427 | 17.6 | 0.147 | 6.1 | 0.523 | 0.91 | <0.001 |
| NO_3_^-^ | 1.845 | 2.006 | 0.161 | 8.4 | 1.440 | 1.582 | 0.085 | 5.4 | 6.8 | 0.320 | 21.2 | 0.126 | 8.3 | 0.444 | 0.94 | <0.001 |
| NH_4_^+^ | 0.368 | 0.430 | 0.062 | 15.6 | 0.319 | 0.382 | 0.052 | 13.6 | 17.1 | 0.106 | 30.2 | 0.056 | 16.1 | 0.184 | 0.76 | <0.001 |
| H^+^ | 0.055 | 0.061 | 0.006 | 9.9 | 0.044 | 0.048 | 0.003 | 5.6 | 11.2 | 0.013 | 28.9 | 0.007 | 15.8 | 0.020 | 0.97 | <0.001 |
| Ca^2+^ | 0.172 | 0.178 | 0.006 | 3.4 | 0.110 | 0.118 | 0.004 | 3.4 | 13.2 | 0.060 | 52.3 | 0.022 | 19.5 | 0.047 | 0.90 | <0.001 |
| Cl^-^ | 0.139 | 0.148 | 0.009 | 6.3 | 0.100 | 0.110 | 0.006 | 5.5 | 13.3 | 0.035 | 33.4 | 0.018 | 16.9 | 0.045 | 0.94 | <0.001 |
| K^+^ | 0.032 | 0.033 | 0.001 | 2.1 | 0.017 | 0.021 | 0.004 | 19.1 | 36.8 | 0.038 | 201.2 | 0.010 | 54.6 | 0.027 | 0.37 | <0.001 |
| Mg^2+^ | 0.028 | 0.030 | 0.002 | 6.7 | 0.019 | 0.020 | 0.002 | 10.0 | 15.4 | 0.012 | 58.8 | 0.004 | 22.8 | 0.010 | 0.84 | <0.001 |
| Na^+^ | 0.045 | 0.042 | -0.003 | -7.0 | 0.026 | 0.022 | <0.001 | 0.0 | 20.8 | 0.024 | 100.5 | 0.007 | 30.9 | 0.012 | 0.91 | 0.004 |
| Depth | mm | mm | mm | % | mm | mm | mm | % | % | mm | % | mm | % | mm |  |  |
| Ppt | 26.544 | 26.623 | 0.079 | 0.3 | 20.066 | 20.700 | 0.292 | 1.4 | 3.8 | 2.112 | 10.4 | 1.035 | 5.1 | 1.890 | 1.00 | <0.001 |
|  |  |  |  |  |  |  |  |  |  |  |  |  |  |  |  |  |

| (b) Penn State University, 1986-2019, cold season | | | | | | | | | | | | | | | | |
| --- | --- | --- | --- | --- | --- | --- | --- | --- | --- | --- | --- | --- | --- | --- | --- | --- |
|  | Mean of NADP | Mean  of CAPMoN | Diff. of mean | Relative diff. of mean | Median of NADP | Median of CAPMoN | Diff. of median | Relative diff. of median | ARB | SD | RSD | MMAD | CoV | P90 | Pearson *r* | *p*-value |
| Ion | mg L^-1^ | mg L^-1^ | mg L^-1^ | % | mg L^-1^ | mg L^-1^ | mg L^-1^ | % | % | mg L^-1^ | % | mg L^-1^ | % | mg L^-1^ |  |  |
| SO_4_^2-^ | 1.597 | 1.775 | 0.178 | 10.6 | 1.360 | 1.370 | 0.090 | 6.6 | 10.6 | 0.411 | 30.1 | 0.182 | 13.4 | 0.600 | 0.94 | <0.001 |
| NO_3_^-^ | 1.849 | 2.241 | 0.392 | 19.2 | 1.467 | 1.767 | 0.226 | 12.8 | 15.3 | 0.512 | 31.6 | 0.249 | 15.4 | 0.930 | 0.92 | <0.001 |
| NH_4_^+^ | 0.300 | 0.375 | 0.075 | 22.2 | 0.204 | 0.271 | 0.050 | 18.5 | 22.3 | 0.098 | 41.3 | 0.050 | 21.2 | 0.167 | 0.92 | <0.001 |
| H^+^ | 0.037 | 0.044 | 0.008 | 19.1 | 0.032 | 0.036 | 0.003 | 9.4 | 13.1 | 0.013 | 37.5 | 0.006 | 18.7 | 0.022 | 0.92 | <0.001 |
| Ca^2+^ | 0.153 | 0.197 | 0.044 | 25.1 | 0.090 | 0.120 | 0.021 | 17.5 | 25.7 | 0.093 | 88.7 | 0.033 | 31.1 | 0.136 | 0.82 | <0.001 |
| Cl^-^ | 0.185 | 0.228 | 0.044 | 21.1 | 0.130 | 0.159 | 0.022 | 13.8 | 19.4 | 0.074 | 51.4 | 0.034 | 23.6 | 0.133 | 0.89 | <0.001 |
| K^+^ | 0.017 | 0.024 | 0.007 | 32.6 | 0.011 | 0.017 | 0.005 | 29.4 | 42.9 | 0.012 | 84.3 | 0.006 | 42.4 | 0.020 | 0.69 | <0.001 |
| Mg^2+^ | 0.022 | 0.030 | 0.008 | 29.8 | 0.013 | 0.019 | 0.004 | 21.1 | 31.2 | 0.014 | 88.7 | 0.006 | 37.1 | 0.023 | 0.87 | <0.001 |
| Na^+^ | 0.079 | 0.090 | 0.011 | 12.9 | 0.050 | 0.056 | 0.006 | 10.7 | 22.6 | 0.045 | 84.0 | 0.016 | 30.8 | 0.057 | 0.80 | <0.001 |
| Depth | mm | mm | mm | % | mm | mm | mm | % | % | mm | % | mm | % | mm |  |  |
| Ppt | 20.657 | 22.219 | 1.562 | 7.3 | 16.764 | 17.400 | 0.660 | 3.8 | 6.9 | 2.806 | 16.4 | 1.563 | 9.2 | 3.714 | 0.90 | <0.001 |

(c) Penn State University, 2005-2019, warm season

| Ion | Mean of NADP | Mean  of CAPMoN | Diff. of mean | Relative diff. of mean | Median of NADP | Median of CAPMoN | Diff. of median | Relative diff. of median | ARB | SD | RSD | MMAD | CoV | P90 | Pearson *r* | *p*-value |
| --- | --- | --- | --- | --- | --- | --- | --- | --- | --- | --- | --- | --- | --- | --- | --- | --- |
|  | mg L^-1^ | mg L^-1^ | mg L^-1^ | % | mg L^-1^ | mg L^-1^ | mg L^-1^ | % | % | mg L^-1^ | % | mg L^-1^ | % | mg L^-1^ |  |  |
| SO_4_^2-^ | 1.757 | 1.757 | <0.001 | 0.0 | 1.234 | 1.257 | 0.033 | 2.6 | 4.7 | 0.246 | 19.7 | 0.080 | 6.4 | 0.236 | 0.77 | <0.001 |
| NO_3_^-^ | 1.188 | 1.278 | 0.090 | 7.3 | 0.976 | 1.082 | 0.073 | 6.8 | 7.8 | 0.197 | 19.2 | 0.084 | 8.1 | 0.318 | 0.81 | <0.001 |
| NH_4_^+^ | 0.376 | 0.411 | 0.035 | 9.0 | 0.308 | 0.356 | 0.047 | 13.1 | 15.7 | 0.092 | 27.7 | 0.050 | 15.2 | 0.147 | 0.76 | <0.001 |
| H^+^ | 0.027 | 0.028 | 0.001 | 3.4 | 0.015 | 0.018 | 0.001 | 7.2 | 18.5 | 0.006 | 37.1 | 0.004 | 22.6 | 0.008 | 0.98 | <0.001 |
| Ca^2+^ | 0.169 | 0.170 | <0.001 | 0.1 | 0.109 | 0.114 | 0.006 | 5.3 | 12.6 | 0.069 | 61.6 | 0.019 | 17.3 | 0.043 | 0.87 | <0.001 |
| Cl^-^ | 0.091 | 0.105 | 0.013 | 13.5 | 0.066 | 0.071 | 0.007 | 9.9 | 14.7 | 0.024 | 35.2 | 0.012 | 17.4 | 0.037 | 0.96 | <0.001 |
| K^+^ | 0.026 | 0.030 | 0.004 | 12.9 | 0.017 | 0.020 | 0.003 | 15.0 | 27.0 | 0.023 | 124.6 | 0.007 | 40.1 | 0.022 | 0.43 | <0.001 |
| Mg^2+^ | 0.027 | 0.029 | 0.001 | 5.1 | 0.017 | 0.020 | 0.002 | 10.0 | 16.2 | 0.011 | 60.7 | 0.003 | 16.0 | 0.010 | 0.82 | <0.001 |
| Na^+^ | 0.033 | 0.038 | 0.005 | 13.4 | 0.018 | 0.018 | 0.001 | 5.6 | 16.7 | 0.014 | 78.0 | 0.004 | 24.7 | 0.012 | 0.98 | <0.001 |
|  | mm | mm | mm | % | mm | mm | mm | % | % | mm | % | mm | % | % |  |  |
| Ppt | 26.1 | 26.7 | 0.5 | 2.0 | 19.6 | 21.0 | 0.5 | 2.3 | 3.6 | 1.5 | 7.6 | 0.9 | 4.5 | 1.9 | 1.00 | <0.001 |

(d) Penn State University, 2005-2019, cold season

| Ion | Mean of NADP | Mean  of CAPMoN | Diff. of mean | Relative diff. of mean | Median of NADP | Median of CAPMoN | Diff. of median | Relative diff. of median | ARB | SD | RSD | MMAD | CoV | P90 | Pearson *r* | *p*-value |  |
| --- | --- | --- | --- | --- | --- | --- | --- | --- | --- | --- | --- | --- | --- | --- | --- | --- | --- |
|  | mg L^-1^ | mg L^-1^ | mg L^-1^ | % | mg L^-1^ | mg L^-1^ | mg L^-1^ | % | % | mg L^-1^ | % | mg L^-1^ | % | mg L^-1^ |  |  | |
| SO_4_^2-^ | 1.111 | 1.245 | 0.133 | 11.3 | 0.746 | 0.887 | 0.083 | 9.4 | 12.9 | 0.313 | 38.3 | 0.101 | 12.4 | 0.393 | 0.94 | <0.001 | |
| NO_3_^-^ | 1.453 | 1.731 | 0.278 | 17.4 | 1.129 | 1.362 | 0.180 | 13.2 | 15.3 | 0.415 | 33.3 | 0.182 | 14.6 | 0.692 | 0.92 | <0.001 | |
| NH_4_^+^ | 0.329 | 0.388 | 0.060 | 16.7 | 0.213 | 0.270 | 0.041 | 15.2 | 19.1 | 0.095 | 39.3 | 0.043 | 17.8 | 0.145 | 0.93 | <0.001 | |
| H^+^ | 0.021 | 0.024 | 0.003 | 13.1 | 0.016 | 0.019 | 0.002 | 10.9 | 18.2 | 0.006 | 36.8 | 0.004 | 20.5 | 0.009 | 0.93 | <0.001 | |
| Ca^2+^ | 0.171 | 0.211 | 0.040 | 20.7 | 0.097 | 0.123 | 0.021 | 17.1 | 26.4 | 0.100 | 91.1 | 0.031 | 28.3 | 0.147 | 0.83 | <0.001 | |
| Cl^-^ | 0.159 | 0.210 | 0.051 | 27.7 | 0.116 | 0.152 | 0.026 | 17.1 | 20.1 | 0.079 | 59.2 | 0.028 | 21.0 | 0.144 | 0.82 | <0.001 | |
| K^+^ | 0.019 | 0.024 | 0.005 | 23.1 | 0.011 | 0.017 | 0.004 | 23.5 | 35.7 | 0.013 | 91.0 | 0.006 | 42.4 | 0.019 | 0.74 | <0.001 | |
| Mg^2+^ | 0.024 | 0.032 | 0.008 | 27.5 | 0.013 | 0.019 | 0.004 | 21.1 | 31.3 | 0.015 | 94.3 | 0.006 | 37.1 | 0.024 | 0.89 | <0.001 | |
| Na^+^ | 0.076 | 0.102 | 0.026 | 29.0 | 0.050 | 0.072 | 0.011 | 15.3 | 21.3 | 0.039 | 63.8 | 0.015 | 24.3 | 0.075 | 0.83 | <0.001 | |
|  | mm | mm | mm | % | mm | mm | mm | % | % | mm | % | mm | % | % |  |  | |
| Ppt | 20.178 | 21.874 | 1.696 | 8.1 | 16.256 | 16.900 | 0.662 | 3.9 | 5.5 | 1.869 | 11.3 | 1.207 | 7.3 | 3.309 | 0.87 | <0.001 | |

(e) Frelingsburg, 2002-2011, warm season

| Ion | Mean of NADP | Mean  of CAPMoN | Diff. of mean | Relative diff. of mean | Median of NADP | Median of CAPMoN | Diff. of median | Relative diff. of median | ARB | SD | RSD | MMAD | CoV | P90 | Pearson *r* | *p*-value |
| --- | --- | --- | --- | --- | --- | --- | --- | --- | --- | --- | --- | --- | --- | --- | --- | --- |
|  | mg L^-1^ | mg L^-1^ | mg L^-1^ | % | mg L^-1^ | mg L^-1^ | mg L^-1^ | % | % | mg L^-1^ | % | mg L^-1^ | % | mg L^-1^ |  |  |
| SO_4_^2-^ | 1.447 | 1.466 | 0.019 | 1.3 | 1.030 | 1.078 | 0.030 | 2.8 | 6.7 | 0.202 | 19.2 | 0.089 | 8.4 | 0.174 | 0.98 | <0.001 |
| NO_3_^-^ | 1.058 | 1.124 | 0.066 | 6.1 | 0.846 | 0.975 | 0.058 | 6.0 | 7.9 | 0.144 | 15.8 | 0.073 | 8.0 | 0.212 | 0.95 | <0.001 |
| NH_4_^+^ | 0.395 | 0.460 | 0.065 | 15.2 | 0.320 | 0.377 | 0.052 | 13.8 | 15.8 | 0.080 | 23.1 | 0.046 | 13.2 | 0.145 | 0.96 | <0.001 |
| H^+^ | 0.020 | 0.021 | 0.002 | 8.2 | 0.013 | 0.016 | 0.002 | 9.7 | 19.9 | 0.006 | 41.4 | 0.004 | 28.4 | 0.008 | 0.95 | <0.001 |
| Ca^2+^ | 0.148 | 0.158 | 0.010 | 6.9 | 0.093 | 0.104 | 0.008 | 7.7 | 16.2 | 0.043 | 43.9 | 0.021 | 21.1 | 0.058 | 0.95 | <0.001 |
| Cl^-^ | 0.058 | 0.062 | 0.004 | 6.6 | 0.043 | 0.045 | 0.002 | 4.4 | 15.9 | 0.017 | 38.2 | 0.010 | 23.6 | 0.017 | 0.96 | <0.001 |
| K^+^ | 0.022 | 0.026 | 0.004 | 15.8 | 0.015 | 0.018 | 0.004 | 22.2 | 36.4 | 0.018 | 110.8 | 0.007 | 44.9 | 0.021 | 0.55 | <0.001 |
| Mg^2+^ | 0.019 | 0.022 | 0.003 | 13.0 | 0.013 | 0.015 | 0.002 | 13.3 | 21.4 | 0.006 | 45.5 | 0.004 | 31.8 | 0.010 | 0.94 | <0.001 |
| Na^+^ | 0.023 | 0.025 | 0.002 | 9.5 | 0.012 | 0.014 | 0.001 | 7.1 | 15.4 | 0.009 | 70.2 | 0.004 | 34.2 | 0.011 | 0.96 | <0.001 |
|  | mm | mm | mm | % | mm | mm | mm | % | % | mm | % | mm | % | % |  |  |
| Ppt | 29.7 | 31.4 | 1.7 | 5.5 | 24.4 | 25.5 | 1.6 | 6.1 | 6.4 | 1.8 | 7.1 | 1.5 | 6.0 | 3.8 | 1.00 | <0.001 |

(f) Frelighsburg, 2002-2011, cold season

| Ion | Mean of NADP | Mean  of CAPMoN | Diff. of mean | Relative diff. of mean | Median of NADP | Median of CAPMoN | Diff. of median | Relative diff. of median | ARB | SD | RSD | MMAD | CoV | P90 | Pearson *r* | *p*-value |
| --- | --- | --- | --- | --- | --- | --- | --- | --- | --- | --- | --- | --- | --- | --- | --- | --- |
|  | mg L^-1^ | mg L^-1^ | mg L^-1^ | % | mg L^-1^ | mg L^-1^ | mg L^-1^ | % | % | mg L^-1^ | % | mg L^-1^ | % | mg L^-1^ |  |  |
| SO_4_^2-^ | 1.253 | 1.234 | -0.019 | -1.5 | 0.879 | 0.968 | 0.030 | 3.1 | 14.9 | 0.432 | 46.7 | 0.199 | 21.6 | 0.378 | 0.91 | 0.195 |
| NO_3_^-^ | 2.031 | 2.158 | 0.126 | 6.0 | 1.387 | 1.546 | 0.118 | 7.6 | 14.6 | 0.739 | 50.4 | 0.289 | 19.7 | 0.853 | 0.91 | <0.001 |
| NH_4_^+^ | 0.414 | 0.451 | 0.038 | 8.7 | 0.238 | 0.305 | 0.040 | 13.1 | 23.6 | 0.160 | 58.9 | 0.081 | 29.7 | 0.187 | 0.92 | <0.001 |
| H^+^ | 0.024 | 0.027 | 0.003 | 11.3 | 0.018 | 0.021 | 0.002 | 9.7 | 21.2 | 0.009 | 44.5 | 0.006 | 30.6 | 0.016 | 0.90 | <0.001 |
| Ca^2+^ | 0.234 | 0.246 | 0.012 | 5.2 | 0.131 | 0.134 | 0.008 | 6.0 | 34.5 | 0.227 | 172.0 | 0.064 | 48.3 | 0.128 | 0.67 | 0.217 |
| Cl^-^ | 0.174 | 0.167 | -0.007 | -4.2 | 0.094 | 0.108 | 0.004 | 3.7 | 20.8 | 0.126 | 125.0 | 0.030 | 29.4 | 0.081 | 0.80 | 0.180 |
| K^+^ | 0.017 | 0.022 | 0.005 | 27.1 | 0.012 | 0.015 | 0.002 | 13.3 | 29.6 | 0.017 | 126.0 | 0.006 | 43.9 | 0.017 | 0.60 | <0.001 |
| Mg^2+^ | 0.024 | 0.025 | 0.002 | 6.6 | 0.015 | 0.016 | 0.001 | 6.5 | 32.8 | 0.016 | 102.0 | 0.006 | 38.9 | 0.015 | 0.80 | 0.054 |
| Na^+^ | 0.102 | 0.097 | -0.005 | -5.3 | 0.046 | 0.057 | 0.003 | 5.3 | 23.4 | 0.084 | 164.0 | 0.018 | 34.7 | 0.049 | 0.77 | 0.076 |
|  | mm | mm | mm | % | mm | mm | mm | % | % | mm | % | mm | % | % |  |  |
| Ppt | 18.4 | 21.6 | 3.2 | 16.0 | 15.0 | 17.8 | 2.0 | 11.5 | 12.4 | 2.9 | 17.9 | 1.9 | 11.3 | 5.6 | 0.88 | <0.001 |

(g) Frelighsburg, 2012-2019, warm season

| Ion | Mean of NADP | Mean  of CAPMoN | Diff. of mean | Relative diff. of mean | Median of NADP | Median of CAPMoN | Diff. of median | Relative diff. of median | ARB | SD | RSD | MMAD | CoV | P90 | Pearson *r* | *p*-value |
| --- | --- | --- | --- | --- | --- | --- | --- | --- | --- | --- | --- | --- | --- | --- | --- | --- |
|  | mg L^-1^ | mg L^-1^ | mg L^-1^ | % | mg L^-1^ | mg L^-1^ | mg L^-1^ | % | % | mg L^-1^ | % | mg L^-1^ | % | mg L^-1^ |  |  |
| SO_4_^2-^ | 0.639 | 0.628 | -0.011 | -1.8 | 0.553 | 0.548 | -0.007 | -1.3 | 4.4 | 0.078 | 14.3 | 0.036 | 6.5 | 0.033 | 0.96 | 0.001 |
| NO_3_^-^ | 0.813 | 0.786 | -0.028 | -3.5 | 0.762 | 0.732 | -0.011 | -1.5 | 3.5 | 0.078 | 10.5 | 0.039 | 5.2 | 0.029 | 0.97 | <0.001 |
| NH_4_^+^ | 0.458 | 0.463 | 0.005 | 1.2 | 0.404 | 0.397 | 0.003 | 0.8 | 6.7 | 0.095 | 23.7 | 0.037 | 9.3 | 0.058 | 0.85 | 0.940 |
| H^+^ | 0.004 | 0.007 | 0.003 | 64.0 | 0.003 | 0.005 | 0.002 | 43.0 | 58.1 | 0.003 | 86.2 | 0.002 | 62.7 | 0.008 | 0.80 | <0.001 |
| Ca^2+^ | 0.137 | 0.132 | -0.004 | -3.2 | 0.110 | 0.101 | -0.003 | -3.0 | 10.4 | 0.041 | 38.7 | 0.013 | 12.6 | 0.028 | 0.90 | 0.067 |
| Cl^-^ | 0.052 | 0.056 | 0.003 | 6.0 | 0.039 | 0.040 | 0.001 | 2.5 | 10.1 | 0.020 | 51.0 | 0.006 | 15.0 | 0.012 | 0.85 | 0.432 |
| K^+^ | 0.032 | 0.040 | 0.008 | 22.6 | 0.019 | 0.018 | 0.001 | 5.6 | 27.0 | 0.066 | 357.1 | 0.007 | 40.1 | 0.011 | 0.15 | 0.764 |
| Mg^2+^ | 0.021 | 0.022 | 0.001 | 4.7 | 0.018 | 0.017 | 0.000 | 0.0 | 14.3 | 0.006 | 35.3 | 0.004 | 21.2 | 0.006 | 0.68 | 0.440 |
| Na^+^ | 0.024 | 0.030 | 0.006 | 20.9 | 0.013 | 0.014 | 0.000 | 0.0 | 15.1 | 0.013 | 94.3 | 0.003 | 22.4 | 0.008 | 0.59 | 0.208 |
|  | mm | mm | mm | % | mm | mm | mm | % | % | mm | % | mm | % | % |  |  |
| Ppt | 28.1 | 28.9 | 0.8 | 2.9 | 22.1 | 23.0 | 0.8 | 3.3 | 3.8 | 1.1 | 5.0 | 0.8 | 3.5 | 2.2 | 1.00 | <0.001 |

(h) Frelighsburg, 2012-2019, cold season

| Ion | Mean of NADP | Mean  of CAPMoN | Diff. of mean | Relative diff. of mean | Median of NADP | Median of CAPMoN | Diff. of median | Relative diff. of median | ARB | SD | RSD | MMAD | CoV | P90 | Pearson *r* | *p*-value |
| --- | --- | --- | --- | --- | --- | --- | --- | --- | --- | --- | --- | --- | --- | --- | --- | --- |
|  | mg L^-1^ | mg L^-1^ | mg L^-1^ | % | mg L^-1^ | mg L^-1^ | mg L^-1^ | % | % | mg L^-1^ | % | mg L^-1^ | % | mg L^-1^ |  |  |
| SO_4_^2-^ | 0.778 | 0.636 | -0.142 | -20.0 | 0.539 | 0.503 | -0.034 | -6.7 | 12.4 | 0.279 | 53.5 | 0.077 | 14.8 | 0.043 | 0.71 | <0.001 |
| NO_3_^-^ | 1.524 | 1.306 | -0.219 | -15.5 | 1.176 | 1.027 | -0.052 | -5.0 | 7.1 | 0.482 | 43.7 | 0.105 | 9.5 | 0.051 | 0.77 | <0.001 |
| NH_4_^+^ | 0.456 | 0.380 | -0.076 | -18.2 | 0.344 | 0.288 | -0.022 | -7.5 | 10.6 | 0.139 | 44.0 | 0.044 | 13.9 | 0.018 | 0.76 | <0.001 |
| H^+^ | 0.010 | 0.012 | 0.002 | 22.9 | 0.006 | 0.008 | 0.002 | 19.6 | 29.4 | 0.005 | 63.6 | 0.003 | 39.6 | 0.008 | 0.88 | <0.001 |
| Ca^2+^ | 0.256 | 0.168 | -0.088 | -41.6 | 0.144 | 0.122 | -0.017 | -14.0 | 16.6 | 0.282 | 212.6 | 0.036 | 27.4 | 0.021 | 0.48 | <0.001 |
| Cl^-^ | 0.213 | 0.120 | -0.093 | -55.6 | 0.083 | 0.081 | -0.006 | -6.8 | 14.1 | 0.350 | 427.7 | 0.018 | 21.8 | 0.011 | 0.51 | <0.001 |
| K^+^ | 0.021 | 0.017 | -0.003 | -18.2 | 0.014 | 0.014 | 0.002 | 14.3 | 21.4 | 0.012 | 87.7 | 0.003 | 21.2 | 0.006 | 0.22 | <0.001 |
| Mg^2+^ | 0.028 | 0.021 | -0.007 | -29.0 | 0.017 | 0.014 | -0.001 | -7.4 | 19.7 | 0.025 | 160.6 | 0.004 | 29.2 | 0.004 | 0.59 | <0.001 |
| Na^+^ | 0.130 | 0.071 | -0.059 | -58.2 | 0.052 | 0.043 | -0.004 | -9.3 | 16.8 | 0.214 | 449.4 | 0.012 | 25.0 | 0.008 | 0.47 | <0.001 |
|  | mm | mm | mm | % | mm | mm | mm | % | % | mm | % | mm | % | % |  |  |
| Ppt | 22.4 | 22.0 | -0.4 | -1.7 | 18.9 | 19.2 | 0.6 | 3.1 | 5.1 | 2.7 | 14.0 | 1.4 | 7.3 | 3.0 | 0.54 | <0.001 |

Table S.3 Statistics for inter-comparisons of precipitation-weighted seasonal mean concentrations and seasonal mean weekly precipitation depths, which are derived with measurements from co-located National Atmospheric Deposition Program (NADP) and Canadian Air and Precipitation Monitoring Network (CAPMoN) sites at Pennsylvania State University, Pennsylvania, USA and Frelighsburg, Quebec, Canada for both warm and cold seasons during selected periods: Penn State, 1986-2019; Penn State, 2005-2019; Frelighsburg, 2002-2011; Frelighsburg, 2012-2019. [Units are in milligrams per liter (mg L^-1^), percent (%), and millimeters (mm), or unitless (R, *p-value*), respectively. Diff., difference; SD, standard deviation from mean; RSD, percent standard deviation relative to mean of NADP and CAPMoN median values; MMAD, Modified median absolute deviation between NADP and CAPMoN values; CoV, non-parametric coefficient of variation; P90, 90^th^ percentile value; Pearson R, coefficient for correlation of NADP and CAPMoN values; *p*-value, the probability of a null hypothesis is true. The null hypothesis here is that there is no difference between the means (or medians) of CAPMoN and NADP weekly concentrations. *p*-values shown are derived with t-test; SO_4_^2-^, sulfate; NO_3_^-^, nitrate; NH_4_^+^, ammonium; H^+^, hydrogen ion; Ca^2+^, calcium; Cl^-^, chloride; K^+^, potassium; Mg^2+^, magnesium; Na^+^, sodium. Data obtained from NADP, Wisconsin State Laboratory of Hygiene at https://nadp.slh.wisc.edu/networks/national-trends-network and Environment and Climate Change Canada at https://www.canada.ca/en/environment-climate-change/services/air-pollution/monitoring-networks-data/canadian-air-precipitation.html, last accessed August 2023.]

(a) Penn State University, 1986-2019, warm season

| Ion | Mean of NADP | Mean  of CAPMoN | Diff. of mean | Relative diff. of mean | Median of NADP | Median of CAPMoN | Diff. of median | Relative diff. of median | ARB | SD | RSD | MMAD | CoV | P90 | Pearson *r* | *p*-value |
| --- | --- | --- | --- | --- | --- | --- | --- | --- | --- | --- | --- | --- | --- | --- | --- | --- |
|  | mg L-1 | mg L^-1^ | mg L^-1^ | % | mg L^-1^ | mg L^-1^ | mg L^-1^ | % | % | mg L^-1^ | % | mg L^-1^ | % | mg L^-1^ |  |  |
| SO_4_^2-^ | 2.315 | 2.375 | 0.060 | 2.6 | 2.301 | 2.428 | 0.052 | 2.1 | 2.6 | 0.177 | 7.6 | 0.082 | 3.5 | 0.237 | 0.99 | 0.057 |
| NO_3_^-^ | 1.368 | 1.489 | 0.120 | 8.4 | 1.285 | 1.413 | 0.120 | 8.5 | 8.9 | 0.062 | 4.4 | 0.076 | 5.3 | 0.205 | 1 | <0.001 |
| NH_4_^+^ | 0.293 | 0.343 | 0.050 | 15.6 | 0.280 | 0.342 | 0.053 | 15.6 | 17.1 | 0.031 | 9.9 | 0.031 | 9.7 | 0.086 | 0.9 | <0.001 |
| H^+^ | 0.046 | 0.051 | 0.005 | 9.8 | 0.047 | 0.051 | 0.003 | 6.6 | 6.9 | 0.006 | 12.5 | 0.004 | 7.2 | 0.013 | 0.99 | <0.001 |
| Ca^2+^ | 0.103 | 0.107 | 0.004 | 3.8 | 0.100 | 0.100 | 0.005 | 5.3 | 8.59 | 0.009 | 8.51 | 0.009 | 8.8 | 0.014 | 0.941 | 0.015 |
| Cl^-^ | 0.103 | 0.110 | 0.007 | 6.9 | 0.097 | 0.107 | 0.007 | 6.7 | 10.66 | 0.012 | 11.11 | 0.010 | 9.8 | 0.021 | 0.964 | 0.001 |
| K^+^ | 0.028 | 0.024 | -0.004 | -14.3 | 0.020 | 0.025 | 0.002 | 9.7 | 32.83 | 0.018 | 67.82 | 0.015 | 67.2 | 0.013 | 0.107 | 0.228 |
| Mg^2+^ | 0.017 | 0.019 | 0.001 | 7.4 | 0.017 | 0.019 | 0.001 | 6.3 | 12.25 | 0.003 | 15.09 | 0.002 | 12.3 | 0.004 | 0.802 | 0.007 |
| Na^+^ | 0.035 | 0.032 | -0.003 | -7.8 | 0.028 | 0.030 | 0.002 | 5.2 | 15.21 | 0.009 | 27.23 | 0.008 | 29.1 | 0.005 | 0.84 | 0.104 |
|  | mm | mm | mm | % | mm | mm | mm | % | % | mm | % | mm | % | % |  |  |
| Ppt | 26.0 | 26.1 | 0.1 | 0.3 | 25.1 | 24.9 | -0.1 | -0.4 | 3.1 | 1.1 | 4.3 | 1.2 | 4.8 | 1.5 | 0.99 | 0.650 |

(b) Penn State University, 1986-2019, cold season

| Ion | Mean of NADP | Mean  of CAPMoN | Diff. of mean | Relative diff. of mean | Median of NADP | Median of CAPMoN | Diff. of median | Relative diff. of median | ARB | SD | RSD | MMAD | CoV | P90 | Pearson *r* | *p*-value |
| --- | --- | --- | --- | --- | --- | --- | --- | --- | --- | --- | --- | --- | --- | --- | --- | --- |
|  | mg L-1 | mg L^-1^ | mg L^-1^ | % | mg L^-1^ | mg L^-1^ | mg L^-1^ | % | % | mg L^-1^ | % | mg L^-1^ | % | mg L^-1^ |  |  |
| SO_4_^2-^ | 1.370 | 1.396 | 0.027 | 1.9 | 1.465 | 1.479 | 0.056 | 3.8 | 6.2 | 0.126 | 9.1 | 0.114 | 8.3 | 0.137 | 0.98 | 0.230 |
| NO_3_^-^ | 1.341 | 1.568 | 0.227 | 15.6 | 1.367 | 1.590 | 0.249 | 15.6 | 16.8 | 0.129 | 8.9 | 0.131 | 9.0 | 0.369 | 0.97 | <0.001 |
| NH_4_^+^ | 0.229 | 0.273 | 0.044 | 17.4 | 0.229 | 0.271 | 0.044 | 16.1 | 17.4 | 0.024 | 9.7 | 0.027 | 10.6 | 0.069 | 0.89 | <0.001 |
| H^+^ | 0.031 | 0.035 | 0.004 | 11.8 | 0.035 | 0.036 | 0.003 | 7.9 | 8.4 | 0.004 | 11.8 | 0.004 | 11.5 | 0.009 | 0.98 | <0.001 |
| Ca^2+^ | 0.099 | 0.120 | 0.021 | 18.9 | 0.101 | 0.118 | 0.021 | 17.6 | 19.2 | 0.019 | 17.4 | 0.017 | 15.3 | 0.039 | 0.823 | <0.001 |
| Cl^-^ | 0.139 | 0.157 | 0.018 | 12.5 | 0.138 | 0.160 | 0.023 | 14.6 | 15.9 | 0.021 | 14.1 | 0.020 | 13.6 | 0.040 | 0.83 | <0.001 |
| K^+^ | 0.014 | 0.019 | 0.005 | 29.7 | 0.012 | 0.019 | 0.005 | 25.8 | 31.9 | 0.004 | 21.9 | 0.003 | 18.2 | 0.009 | 0.733 | <0.001 |
| Mg^2+^ | 0.015 | 0.019 | 0.003 | 20.0 | 0.015 | 0.018 | 0.004 | 21.3 | 24.8 | 0.003 | 16.5 | 0.003 | 17.3 | 0.006 | 0.744 | <0.001 |
| Na^+^ | 0.060 | 0.062 | 0.002 | 3.3 | 0.056 | 0.064 | 0.009 | 13.5 | 22.7 | 0.017 | 27.6 | 0.019 | 31.4 | 0.018 | 0.514 | 0.492 |
|  | mm | mm | mm | % | mm | mm | mm | % | % | mm | % | mm | % | % |  |  |
| Ppt | 20.7 | 22.4 | 1.7 | 7.7 | 19.8 | 21.7 | 0.9 | 4.4 | 4.6 | 2.2 | 10.2 | 1.7 | 7.9 | 4.4 | 0.92 | <0.001 |

(c) Penn State University, 2005-2019, warm season

| Ion | Mean of NADP | Mean  of CAPMoN | Diff. of mean | Relative diff. of mean | Median of NADP | Median of CAPMoN | Diff. of median | Relative diff. of median | ARB | SD | RSD | MMAD | CoV | P90 | Pearson *r* | *p*-value |
| --- | --- | --- | --- | --- | --- | --- | --- | --- | --- | --- | --- | --- | --- | --- | --- | --- |
|  | mg L-1 | mg L^-1^ | mg L^-1^ | % | mg L^-1^ | mg L^-1^ | mg L^-1^ | % | % | mg L^-1^ | % | mg L^-1^ | % | mg L^-1^ |  |  |
| SO_4_^2-^ | 1.259 | 1.304 | 0.045 | 3.5 | 0.942 | 0.942 | 0.035 | 3.7 | 3.7 | 0.053 | 4.1 | 0.042 | 3.3 | 0.117 | 1.00 | 0.005 |
| NO_3_^-^ | 0.837 | 0.919 | 0.081 | 9.3 | 0.783 | 0.874 | 0.064 | 7.4 | 7.8 | 0.044 | 5.0 | 0.059 | 6.7 | 0.133 | 0.99 | <0.001 |
| NH_4_^+^ | 0.279 | 0.313 | 0.034 | 11.4 | 0.276 | 0.302 | 0.027 | 9.1 | 9.5 | 0.029 | 9.6 | 0.026 | 8.9 | 0.071 | 0.83 | <0.001 |
| H^+^ | 0.021 | 0.022 | 0.001 | 5.1 | 0.013 | 0.016 | 0.002 | 14.5 | 17.0 | 0.002 | 10.3 | 0.002 | 10.2 | 0.003 | 1.00 | 0.078 |
| Ca^2+^ | 0.099 | 0.104 | 0.005 | 4.5 | 0.093 | 0.096 | 0.006 | 6.2 | 8.6 | 0.007 | 7.4 | 0.008 | 8.6 | 0.013 | 0.95 | 0.034 |
| Cl^-^ | 0.066 | 0.076 | 0.010 | 14.7 | 0.068 | 0.074 | 0.008 | 11.5 | 11.9 | 0.007 | 9.2 | 0.007 | 10.1 | 0.019 | 0.99 | <0.001 |
| K^+^ | 0.023 | 0.023 | <0.001 | 0.4 | 0.019 | 0.022 | 0.002 | 10.8 | 22.3 | 0.009 | 38.4 | 0.007 | 32.5 | 0.007 | 0.68 | 0.971 |
| Mg^2+^ | 0.016 | 0.018 | 0.002 | 11.6 | 0.016 | 0.018 | 0.001 | 8.0 | 8.3 | 0.002 | 9.2 | 0.002 | 9.1 | 0.004 | 0.93 | <0.001 |
| Na^+^ | 0.026 | 0.028 | 0.003 | 10.1 | 0.023 | 0.026 | 0.002 | 7.8 | 8.4 | 0.003 | 9.5 | 0.001 | 5.8 | 0.005 | 0.97 | 0.001 |
|  | mm | mm | mm | % | mm | mm | mm | % | % | mm | % | mm | % | % |  |  |
| Ppt | 25.9 | 26.5 | 0.6 | 2.1 | 25.7 | 25.8 | 0.6 | 2.3 | 2.8 | 0.9 | 3.3 | 1.1 | 4.1 | 1.5 | 0.99 | 0.028 |

(d) Penn State University, 2005-2019, cold season

| Ion | Mean of NADP | Mean  of CAPMoN | Diff. of mean | Relative diff. of mean | Median of NADP | Median of CAPMoN | Diff. of median | Relative diff. of median | ARB | SD | RSD | MMAD | CoV | P90 | Pearson *r* | *p*-value |
| --- | --- | --- | --- | --- | --- | --- | --- | --- | --- | --- | --- | --- | --- | --- | --- | --- |
|  | mg L-1 | mg L^-1^ | mg L^-1^ | % | mg L^-1^ | mg L^-1^ | mg L^-1^ | % | % | mg L^-1^ | % | mg L^-1^ | % | mg L^-1^ |  |  |
| SO_4_^2-^ | 0.889 | 0.935 | 0.046 | 5.0 | 0.746 | 0.809 | 0.059 | 7.2 | 9.2 | 0.082 | 8.9 | 0.057 | 6.3 | 0.122 | 0.99 | 0.046 |
| NO_3_^-^ | 1.016 | 1.176 | 0.159 | 14.5 | 1.011 | 1.190 | 0.150 | 12.6 | 13.6 | 0.123 | 11.2 | 0.083 | 7.6 | 0.311 | 0.95 | <0.001 |
| NH_4_^+^ | 0.249 | 0.281 | 0.032 | 12.1 | 0.256 | 0.282 | 0.032 | 11.5 | 12.0 | 0.025 | 9.6 | 0.027 | 10.1 | 0.065 | 0.90 | <0.001 |
| H^+^ | 0.017 | 0.019 | 0.001 | 7.8 | 0.014 | 0.017 | 0.002 | 8.6 | 13.6 | 0.002 | 12.6 | 0.002 | 10.9 | 0.004 | 0.98 | 0.030 |
| Ca^2+^ | 0.104 | 0.124 | 0.020 | 17.4 | 0.101 | 0.127 | 0.025 | 19.5 | 21.7 | 0.017 | 15.0 | 0.019 | 16.7 | 0.037 | 0.85 | 0.001 |
| Cl^-^ | 0.119 | 0.143 | 0.024 | 18.1 | 0.117 | 0.140 | 0.025 | 17.9 | 21.8 | 0.023 | 17.8 | 0.013 | 9.9 | 0.042 | 0.81 | 0.001 |
| K^+^ | 0.017 | 0.020 | 0.003 | 17.4 | 0.015 | 0.019 | 0.004 | 18.9 | 22.4 | 0.004 | 22.8 | 0.003 | 17.8 | 0.007 | 0.77 | 0.010 |
| Mg^2+^ | 0.016 | 0.019 | 0.003 | 19.2 | 0.015 | 0.018 | 0.004 | 20.7 | 23.4 | 0.003 | 15.2 | 0.002 | 11.1 | 0.006 | 0.78 | <0.001 |
| Na^+^ | 0.059 | 0.069 | 0.011 | 17.0 | 0.056 | 0.066 | 0.015 | 22.0 | 24.0 | 0.015 | 23.4 | 0.006 | 10.2 | 0.021 | 0.50 | 0.014 |
|  | mm | mm | mm | % | mm | mm | mm | % | % | mm | % | mm | % | % |  |  |
| Ppt | 20.3 | 22.1 | 1.8 | 8.6 | 19.8 | 20.3 | 1.0 | 5.1 | 5.2 | 2.5 | 11.8 | 1.5 | 6.9 | 5.0 | 0.90 | 0.013 |

(e) Frelighsburg, 2002-2011, warm season

| Ion | Mean of NADP | Mean  of CAPMoN | Diff. of mean | Relative diff. of mean | Median of NADP | Median of CAPMoN | Diff. of median | Relative diff. of median | ARB | SD | RSD | MMAD | CoV | P90 | Corr. | *p*-value |
| --- | --- | --- | --- | --- | --- | --- | --- | --- | --- | --- | --- | --- | --- | --- | --- | --- |
|  | mg L^-1^ | mg L^-1^ | mg L^-1^ | % | mg L^-1^ | mg L^-1^ | mg L^-1^ | % | % | mg L^-1^ | % | mg L^-1^ | % | mg L^-1^ |  |  |
| SO_4_^2-^ | 1.137 | 1.171 | 0.034 | 2.9 | 1.145 | 1.120 | 0.036 | 3.2 | 4.7 | 0.062 | 5.4 | 0.047 | 4.1 | 0.120 | 0.99 | 0.118 |
| NO_3_^-^ | 0.793 | 0.867 | 0.074 | 9.0 | 0.737 | 0.804 | 0.070 | 8.7 | 9.1 | 0.043 | 5.2 | 0.038 | 4.6 | 0.128 | 0.99 | 0.000 |
| NH_4_^+^ | 0.302 | 0.354 | 0.052 | 15.8 | 0.309 | 0.368 | 0.055 | 14.9 | 16.2 | 0.017 | 5.1 | 0.014 | 4.4 | 0.067 | 0.93 | 0.000 |
| H^+^ | 0.017 | 0.018 | 0.001 | 6.7 | 0.016 | 0.016 | 0.001 | 8.8 | 13.5 | 0.003 | 16.3 | 0.002 | 14.6 | 0.004 | 0.93 | 0.225 |
| Ca^2+^ | 0.093 | 0.105 | 0.012 | 12.5 | 0.088 | 0.094 | 0.015 | 15.9 | 16.5 | 0.010 | 10.2 | 0.010 | 11.4 | 0.021 | 0.94 | 0.004 |
| Cl^-^ | 0.042 | 0.045 | 0.003 | 7.5 | 0.041 | 0.046 | 0.004 | 8.9 | 9.5 | 0.004 | 9.1 | 0.004 | 9.3 | 0.007 | 0.88 | 0.028 |
| K^+^ | 0.019 | 0.020 | 0.001 | 7.5 | 0.018 | 0.019 | 0.001 | 5.5 | 18.8 | 0.004 | 21.8 | 0.005 | 24.1 | 0.007 | 0.62 | 0.307 |
| Mg^2+^ | 0.013 | 0.015 | 0.003 | 18.3 | 0.012 | 0.015 | 0.003 | 18.6 | 20.7 | 0.001 | 7.6 | 0.001 | 7.3 | 0.004 | 0.93 | 0.000 |
| Na^+^ | 0.016 | 0.018 | 0.002 | 10.1 | 0.015 | 0.018 | 0.002 | 8.9 | 9.7 | 0.002 | 9.7 | 0.002 | 12.0 | 0.004 | 0.93 | 0.010 |
|  | mm | mm | mm | % | mm | mm | mm | % | % | mm | % | mm | % | % |  |  |
| Ppt | 29.8 | 31.5 | 1.7 | 5.6 | 28.3 | 30.2 | 1.7 | 5.6 | 5.8 | 0.6 | 1.9 | 0.8 | 2.5 | 2.3 | 1.00 | 0.000 |

(f) Frelighsburg, 2002-2011, cold season

| Ion | Mean of NADP | Mean  of CAPMoN | Diff. of mean | Relative diff. of mean | Median of NADP | Median of CAPMoN | Diff. of median | Relative diff. of median | ARB | SD | RSD | MMAD | CoV | P90 | Pearson *r* | *p*-value |
| --- | --- | --- | --- | --- | --- | --- | --- | --- | --- | --- | --- | --- | --- | --- | --- | --- |
|  | mg L-1 | mg L^-1^ | mg L^-1^ | % | mg L^-1^ | mg L^-1^ | mg L^-1^ | % | % | mg L^-1^ | % | mg L^-1^ | % | mg L^-1^ |  |  |
| SO_4_^2-^ | 1.026 | 1.067 | 0.041 | 3.9 | 0.957 | 1.102 | 0.057 | 5.2 | 7.1 | 0.147 | 14.1 | 0.072 | 6.9 | 0.172 | 0.92 | 0.401 |
| NO_3_^-^ | 1.465 | 1.697 | 0.232 | 14.7 | 1.255 | 1.528 | 0.222 | 14.5 | 18.2 | 0.219 | 13.8 | 0.097 | 6.1 | 0.471 | 0.95 | 0.008 |
| NH_4_^+^ | 0.283 | 0.334 | 0.051 | 16.6 | 0.263 | 0.332 | 0.049 | 14.7 | 16.8 | 0.048 | 15.6 | 0.028 | 9.2 | 0.106 | 0.73 | 0.008 |
| H^+^ | 0.022 | 0.024 | 0.002 | 10.6 | 0.021 | 0.023 | 0.002 | 10.3 | 10.8 | 0.002 | 9.4 | 0.002 | 10.3 | 0.005 | 0.98 | 0.006 |
| Ca^2+^ | 0.130 | 0.152 | 0.022 | 15.6 | 0.114 | 0.148 | 0.025 | 17.2 | 36.4 | 0.062 | 43.9 | 0.046 | 35.6 | 0.087 | -0.07 | 0.291 |
| Cl^-^ | 0.103 | 0.113 | 0.010 | 9.0 | 0.086 | 0.120 | 0.017 | 14.3 | 17.1 | 0.037 | 34.3 | 0.015 | 14.5 | 0.038 | 0.61 | 0.430 |
| K^+^ | 0.011 | 0.015 | 0.004 | 31.2 | 0.011 | 0.014 | 0.004 | 29.3 | 33.8 | 0.003 | 21.3 | 0.003 | 26.2 | 0.007 | 0.43 | 0.001 |
| Mg^2+^ | 0.014 | 0.016 | 0.002 | 15.0 | 0.013 | 0.016 | 0.003 | 16.0 | 30.8 | 0.005 | 30.1 | 0.004 | 30.4 | 0.007 | 0.41 | 0.149 |
| Na^+^ | 0.058 | 0.061 | 0.003 | 4.7 | 0.047 | 0.062 | 0.009 | 14.1 | 25.4 | 0.022 | 37.5 | 0.016 | 30.1 | 0.024 | 0.54 | 0.702 |
|  | mm | mm | mm | % | mm | mm | mm | % | % | mm | % | mm | % | % |  |  |
| Ppt | 18.5 | 21.8 | 3.3 | 16.2 | 18.4 | 21.2 | 2.8 | 13.1 | 14.0 | 1.6 | 8.2 | 1.0 | 4.8 | 4.3 | 0.85 | 0.000 |

(g) Frelighsburg, 2012-2019, warm season

| Ion | Mean of NADP | Mean  of CAPMoN | Diff. of mean | Relative diff. of mean | Median of NADP | Median of CAPMoN | Diff. of median | Relative diff. of median | ARB | SD | RSD | MMAD | CoV | P90 | Pearson *r* | *p*-value |
| --- | --- | --- | --- | --- | --- | --- | --- | --- | --- | --- | --- | --- | --- | --- | --- | --- |
|  | mg L-1 | mg L^-1^ | mg L^-1^ | % | mg L^-1^ | mg L^-1^ | mg L^-1^ | % | % | mg L^-1^ | % | mg L^-1^ | % | mg L^-1^ |  |  |
| SO_4_^2-^ | 0.558 | 0.561 | 0.003 | 0.5 | 0.545 | 0.549 | -0.008 | -1.4 | 1.9 | 0.022 | 4.0 | 0.020 | 3.5 | 0.033 | 1.00 | 0.700 |
| NO_3_^-^ | 0.641 | 0.644 | 0.003 | 0.4 | 0.632 | 0.615 | 0.007 | 1.2 | 4.3 | 0.034 | 5.3 | 0.040 | 6.3 | 0.045 | 0.98 | 0.800 |
| NH_4_^+^ | 0.364 | 0.363 | -0.001 | -0.2 | 0.356 | 0.348 | <0.001 | 0.1 | 5.1 | 0.021 | 5.7 | 0.027 | 7.5 | 0.020 | 0.97 | 0.920 |
| H^+^ | 0.005 | 0.008 | 0.003 | 47.9 | 0.004 | 0.007 | 0.003 | 43.8 | 58.6 | 0.001 | 14.3 | 0.001 | 19.0 | 0.004 | 0.94 | <0.001 |
| Ca^2+^ | 0.100 | 0.103 | 0.003 | 3.2 | 0.090 | 0.087 | <0.001 | -0.5 | 11.0 | 0.016 | 16.1 | 0.019 | 21.7 | 0.025 | 0.93 | 0.549 |
| Cl^-^ | 0.041 | 0.043 | 0.002 | 3.8 | 0.043 | 0.043 | 0.001 | 1.9 | 3.8 | 0.003 | 6.6 | 0.003 | 6.8 | 0.005 | 0.90 | 0.099 |
| K^+^ | 0.032 | 0.026 | -0.007 | -22.7 | 0.029 | 0.020 | -0.002 | -12.5 | 16.6 | 0.013 | 44.0 | 0.008 | 32.9 | 0.002 | 0.70 | 0.137 |
| Mg^2+^ | 0.015 | 0.016 | 0.001 | 5.3 | 0.014 | 0.015 | 0.001 | 3.6 | 12.3 | 0.002 | 14.2 | 0.002 | 14.9 | 0.004 | 0.88 | 0.269 |
| Na^+^ | 0.018 | 0.020 | 0.001 | 6.6 | 0.019 | 0.020 | 0.001 | 3.2 | 3.6 | 0.002 | 12.1 | 0.002 | 11.2 | 0.005 | 0.89 | 0.120 |
|  | mm | mm | mm | % | mm | mm | mm | % | % | mm | % | mm | % | % |  |  |
| Ppt | 28.7 | 29.7 | 1.0 | 3.5 | 27.0 | 28.2 | 0.9 | 3.2 | 3.3 | 0.6 | 2.0 | 0.7 | 2.4 | 1.7 | 1.00 | <0.001 |

(h) Frelighsburg, 2012-2019, cold season

| Ion | Mean of NADP | Mean  of CAPMoN | Diff. of mean | Relative diff. of mean | Median of NADP | Median of CAPMoN | Diff. of median | Relative diff. of median | ARB | SD | RSD | MMAD | CoV | P90 | Pearson *r* | *p*-value |
| --- | --- | --- | --- | --- | --- | --- | --- | --- | --- | --- | --- | --- | --- | --- | --- | --- |
|  | mg L-1 | mg L^-1^ | mg L^-1^ | % | mg L^-1^ | mg L^-1^ | mg L^-1^ | % | % | mg L^-1^ | % | mg L^-1^ | % | mg L^-1^ |  |  |
| SO_4_^2-^ | 0.596 | 0.540 | -0.056 | -9.8 | 0.592 | 0.558 | -0.041 | -7.4 | 9.1 | 0.122 | 21.0 | 0.090 | 15.9 | 0.054 | 0.71 | 0.180 |
| NO_3_^-^ | 1.108 | 1.010 | -0.099 | -9.3 | 1.099 | 1.003 | -0.089 | -8.9 | 16.4 | 0.233 | 22.0 | 0.142 | 13.4 | 0.194 | 0.49 | 0.210 |
| NH_4_^+^ | 0.335 | 0.300 | -0.032 | -10.0 | 0.339 | 0.320 | -0.033 | -10.3 | 13.0 | 0.068 | 21.0 | 0.041 | 12.8 | 0.036 | 0.12 | 0.170 |
| H^+^ | 0.009 | 0.010 | 0.001 | 10.1 | 0.010 | 0.010 | 0.002 | 18.8 | 21.8 | 0.003 | 31.0 | 0.002 | 19.6 | 0.003 | 0.62 | 0.330 |
| Ca^2+^ | 0.157 | 0.129 | -0.028 | -19.9 | 0.140 | 0.125 | -0.012 | -9.5 | 15.8 | 0.060 | 41.7 | 0.041 | 30.7 | 0.013 | 0.19 | 0.166 |
| Cl^-^ | 0.114 | 0.090 | -0.024 | -23.3 | 0.102 | 0.085 | -0.016 | -19.2 | 19.3 | 0.052 | 50.8 | 0.026 | 28.3 | 0.018 | 0.15 | 0.182 |
| K^+^ | 0.014 | 0.014 | 0.001 | 4.6 | 0.013 | 0.014 | 0.001 | 8.7 | 13.7 | 0.003 | 19.3 | 0.002 | 15.6 | 0.003 | 0.53 | 0.469 |
| Mg^2+^ | 0.018 | 0.016 | -0.002 | -11.9 | 0.016 | 0.016 | -0.001 | -8.4 | 14.3 | 0.006 | 33.0 | 0.002 | 15.5 | 0.003 | -0.03 | 0.285 |
| Na^+^ | 0.068 | 0.053 | -0.015 | -25.5 | 0.058 | 0.048 | -0.010 | -19.9 | 19.4 | 0.033 | 54.2 | 0.015 | 28.7 | 0.009 | 0.12 | 0.172 |
|  | mm | mm | mm | % | mm | mm | mm | % | % | mm | % | mm | % | % |  |  |
| Ppt | 22.2 | 23.2 | 0.9 | 4.1 | 20.9 | 23.6 | 0.9 | 3.8 | 5.7 | 4.5 | 20.0 | 1.4 | 6.2 | 4.3 | 0.57 | 0.530 |

Table S.4 Statistics for inter-comparisons of annual and seasonal deposition, which are derived with measurements from co-located National Atmospheric Deposition Program (NADP) and Canadian Air and Precipitation Monitoring Network (CAPMoN) sites at Pennsylvania State University, Pennsylvania, USA and Frelighsburg, Quebec, Canada for different seasons during selected periods: Penn State, 1986-2019; Penn State, 2005-2019; Frelighsburg, 2002-2011; Frelighsburg, 2012-2019. [Warm season, May-October; Cold season, November-April. Units are in milligrams per liter (mg L^-1^), percent (%), and millimeters (mm), or unitless (R, *p-value*), respectively. Diff., difference; SD, standard deviation from mean; RSD, percent standard deviation relative to mean of NADP and CAPMoN median values; MMAD, Modified median absolute deviation between NADP and CAPMoN values; CoV, non-parametric coefficient of variation; P90, 90^th^ percentile value; Pearson R, coefficient for correlation of NADP and CAPMoN values; *p*-value, the probability of a null hypothesis is true. The null hypothesis here is that there is no difference between the means (or medians) of CAPMoN and NADP weekly concentrations. *p*-values shown are for t-test; SO_4_^2-^, sulfate; NO_3_^-^, nitrate; NH_4_^+^, ammonium; H^+^, hydrogen ion; Ca^2+^, calcium; Cl^-^, chloride; K^+^, potassium; Mg^2+^, magnesium; Na^+^, sodium. Data obtained from NADP, Wisconsin State Laboratory of Hygiene at https://nadp.slh.wisc.edu/networks/national-trends-network and Environment and Climate Change Canada at https://www.canada.ca/en/environment-climate-change/services/air-pollution/monitoring-networks-data/canadian-air-precipitation.html, last accessed August 2023.]

(a) Penn State University, 1986-2019, annual deposition

| Ion | Mean of NADP | Mean  of CAPMoN | Diff. of mean | Relative diff. of mean | Median of NADP | Median of CAPMoN | Diff. of median | Relative diff. of median | ARB | SD | RSD | MMAD | CoV | P90 | Pearson *r* | *p*-value |
| --- | --- | --- | --- | --- | --- | --- | --- | --- | --- | --- | --- | --- | --- | --- | --- | --- |
|  | kg ha^-1^ | kg H^+^a^-1^ | kg ha^-1^ | % | kg ha^-1^ | kg ha^-1^ | kg ha^-1^ | % | % | kg ha^-1^ | % | kg ha^-1^ | % | kg ha^-1^ |  |  |
| SO_4_^2-^ | 16.300 | 17.180 | 0.881 | 5.3 | 18.220 | 18.260 | 0.930 | 5.1 | 5.4 | 1.032 | 6.2 | 0.750 | 4.5 | 1.809 | 0.99 | <0.001 |
| NO_3_^-^ | 11.800 | 13.800 | 2.000 | 15.6 | 12.400 | 14.390 | 2.145 | 14.9 | 16.0 | 0.856 | 6.7 | 1.024 | 8.0 | 2.994 | 0.99 | <0.001 |
| NH_4_^+^ | 2.360 | 2.860 | 0.507 | 19.4 | 2.400 | 2.780 | 0.477 | 17.2 | 18.4 | 0.228 | 8.7 | 0.245 | 9.4 | 0.768 | 0.95 | <0.001 |
| H^+^ | 0.340 | 0.380 | 0.044 | 12.3 | 0.370 | 0.390 | 0.035 | 8.8 | 9.2 | 0.042 | 11.6 | 0.041 | 11.5 | 0.099 | 0.98 | <0.001 |
| Ca^2+^ | 0.900 | 1.042 | 0.142 | 14.7 | 0.892 | 1.039 | 0.144 | 13.9 | 15.0 | 0.079 | 8.1 | 0.072 | 7.4 | 0.248 | 0.96 | <0.001 |
| Cl^-^ | 1.053 | 1.219 | 0.166 | 14.6 | 1.064 | 1.161 | 0.158 | 13.6 | 14.2 | 0.113 | 9.9 | 0.107 | 9.7 | 0.327 | 0.96 | <0.001 |
| K^+^ | 0.193 | 0.201 | 0.009 | 4.5 | 0.188 | 0.194 | 0.033 | 17.1 | 27.8 | 0.080 | 40.7 | 0.073 | 38.0 | 0.088 | 0.51 | 0.528 |
| Mg^2+^ | 0.144 | 0.172 | 0.028 | 17.5 | 0.140 | 0.169 | 0.027 | 16.2 | 17.7 | 0.018 | 11.4 | 0.014 | 9.3 | 0.050 | 0.91 | <0.001 |
| Na^+^ | 0.412 | 0.438 | 0.026 | 6.1 | 0.373 | 0.414 | 0.052 | 12.4 | 21.0 | 0.108 | 25.5 | 0.117 | 29.8 | 0.132 | 0.73 | 0.174 |

(b) Penn State University, 1986-2019, warm-season deposition

| Ion | Mean of NADP | Mean  of CAPMoN | Diff. of mean | Relative diff. of mean | Median of NADP | Median of CAPMoN | Diff. of median | Relative diff. of median | ARB | SD | RSD | MMAD | CoV | P90 | Corr. | *p*-value |
| --- | --- | --- | --- | --- | --- | --- | --- | --- | --- | --- | --- | --- | --- | --- | --- | --- |
|  | kg ha^-1^ | kg ha^-1^ | kg ha^-1^ | % | kg ha^-1^ | kg ha^-1^ | kg ha^-1^ | % | % | kg ha^-1^ | % | kg ha^-1^ | % | kg ha^-1^ |  |  |
| SO_4_^2-^ | 10.760 | 11.070 | 0.308 | 2.8 | 11.460 | 11.420 | 0.236 | 2.1 | 2.8 | 0.746 | 6.8 | 0.415 | 3.8 | 0.928 | 0.99 | 0.022 |
| NO_3_^-^ | 6.360 | 6.940 | 0.578 | 8.7 | 6.760 | 7.010 | 0.491 | 7.0 | 7.1 | 0.397 | 6.0 | 0.309 | 4.7 | 0.988 | 0.99 | <0.001 |
| NH_4_^+^ | 1.420 | 1.660 | 0.246 | 16.0 | 1.450 | 1.650 | 0.239 | 14.4 | 15.4 | 0.150 | 9.7 | 0.180 | 11.7 | 0.446 | 0.96 | <0.001 |
| H^+^ | 0.210 | 0.230 | 0.018 | 8.3 | 0.240 | 0.240 | 0.013 | 5.4 | 6.8 | 0.025 | 11.4 | 0.016 | 7.3 | 0.055 | 0.98 | <0.001 |
| Ca^2+^ | 0.495 | 0.514 | 0.019 | 3.8 | 0.478 | 0.502 | 0.021 | 4.2 | 6.2 | 0.039 | 7.7 | 0.035 | 7.1 | 0.059 | 0.97 | 0.008 |
| Cl^-^ | 0.483 | 0.519 | 0.037 | 7.3 | 0.453 | 0.490 | 0.041 | 8.3 | 10.0 | 0.050 | 10.0 | 0.054 | 11.4 | 0.091 | 0.98 | <0.001 |
| K^+^ | 0.135 | 0.119 | -0.017 | -13.0 | 0.105 | 0.117 | 0.012 | 10.4 | 30.6 | 0.078 | 61.3 | 0.068 | 61.3 | 0.054 | 0.47 | 0.225 |
| Mg^2+^ | 0.082 | 0.089 | 0.007 | 8.3 | 0.083 | 0.091 | 0.009 | 9.7 | 11.9 | 0.010 | 12.2 | 0.009 | 9.9 | 0.019 | 0.92 | <0.001 |

(c) Penn State University, 1986-2019, cold-season deposition

| Ion | Mean of NADP | Mean  of CAPMoN | Diff. of mean | Relative diff. of mean | Median of NADP | Median of CAPMoN | Diff. of median | Relative diff. of median | ARB | SD | RSD | MMAD | CoV | P90 | Pearson *r* | *p*-value |
| --- | --- | --- | --- | --- | --- | --- | --- | --- | --- | --- | --- | --- | --- | --- | --- | --- |
|  | kg ha^-1^ | kg ha^-1^ | kg ha^-1^ | % | kg ha^-1^ | kg ha^-1^ | kg ha^-1^ | % | % | kg ha^-1^ | % | kg ha^-1^ | % | kg ha^-1^ |  |  |
| SO_4_^2-^ | 5.540 | 6.110 | 0.573 | 9.8 | 6.120 | 6.870 | 0.549 | 8.0 | 8.8 | 0.470 | 8.1 | 0.461 | 7.9 | 1.158 | 0.99 | <0.001 |
| NO_3_^-^ | 5.440 | 6.860 | 1.423 | 23.1 | 5.560 | 7.220 | 1.399 | 19.0 | 21.9 | 0.690 | 11.2 | 0.762 | 12.4 | 2.217 | 0.97 | <0.001 |
| NH_4_^+^ | 0.940 | 1.200 | 0.261 | 24.4 | 0.910 | 1.100 | 0.230 | 21.0 | 22.9 | 0.110 | 10.7 | 0.109 | 10.2 | 0.432 | 0.94 | <0.001 |
| H^+^ | 0.130 | 0.150 | 0.026 | 18.7 | 0.140 | 0.160 | 0.022 | 14.0 | 14.9 | 0.020 | 14.4 | 0.024 | 16.9 | 0.052 | 0.98 | <0.001 |
| Ca^2+^ | 0.405 | 0.528 | 0.123 | 26.4 | 0.374 | 0.527 | 0.128 | 24.3 | 28.4 | 0.068 | 14.6 | 0.073 | 16.1 | 0.203 | 0.92 | <0.001 |
| Cl^-^ | 0.571 | 0.700 | 0.130 | 20.4 | 0.554 | 0.696 | 0.124 | 17.8 | 19.8 | 0.087 | 13.7 | 0.058 | 9.3 | 0.272 | 0.92 | <0.001 |
| K^+^ | 0.057 | 0.083 | 0.025 | 36.2 | 0.050 | 0.077 | 0.024 | 30.8 | 38.1 | 0.019 | 27.8 | 0.013 | 20.3 | 0.043 | 0.76 | <0.001 |
| Mg^2+^ | 0.062 | 0.083 | 0.021 | 28.3 | 0.059 | 0.080 | 0.021 | 26.2 | 30.2 | 0.011 | 15.4 | 0.012 | 17.1 | 0.034 | 0.91 | <0.001 |
| Na^+^ | 0.251 | 0.283 | 0.033 | 12.2 | 0.235 | 0.269 | 0.045 | 16.7 | 23.7 | 0.078 | 29.3 | 0.071 | 28.1 | 0.112 | 0.75 | 0.021 |
|  |  |  |  |  |  |  |  |  |  |  |  |  |  |  |  |  |

(d) Penn State University, 2005-2019, annual deposition

| Ion | Mean of NADP | Mean  of CAPMoN | Diff. of mean | Relative diff. of mean | Median of NADP | Median of CAPMoN | Diff. of median | Relative diff. of median | ARB | SD | RSD | MMAD | CoV | P90 | Pearson *r* | *p*-value |
| --- | --- | --- | --- | --- | --- | --- | --- | --- | --- | --- | --- | --- | --- | --- | --- | --- |
|  | kg ha^-1^ | kg ha^-1^ | kg ha^-1^ | % | kg ha^-1^ | kg ha^-1^ | kg ha^-1^ | % | % | kg ha^-1^ | % | kg ha^-1^ | % | kg ha^-1^ |  |  |
| SO_4_^2-^ | 9.769 | 10.634 | 0.865 | 8.5 | 7.961 | 8.530 | 0.717 | 8.4 | 8.7 | 0.538 | 5.3 | 0.642 | 6.3 | 1.588 | 1.00 | <0.001 |
| NO_3_^-^ | 8.317 | 9.904 | 1.587 | 17.4 | 7.471 | 8.755 | 1.322 | 15.1 | 16.3 | 0.780 | 8.6 | 0.795 | 8.7 | 2.744 | 0.98 | <0.001 |
| NH_4_^+^ | 2.426 | 2.855 | 0.429 | 16.3 | 2.503 | 2.796 | 0.390 | 14.0 | 14.7 | 0.216 | 8.2 | 0.250 | 9.5 | 0.721 | 0.95 | <0.001 |
| H^+^ | 0.173 | 0.194 | 0.021 | 11.3 | 0.108 | 0.144 | 0.026 | 17.7 | 20.2 | 0.018 | 9.6 | 0.020 | 10.9 | 0.039 | 0.99 | <0.001 |
| Ca^2+^ | 0.908 | 1.067 | 0.159 | 16.1 | 0.856 | 1.042 | 0.176 | 16.9 | 18.6 | 0.071 | 7.2 | 0.068 | 7.2 | 0.238 | 0.95 | <0.001 |
| Cl^-^ | 0.814 | 1.025 | 0.211 | 22.9 | 0.826 | 1.121 | 0.209 | 18.6 | 21.4 | 0.098 | 10.7 | 0.124 | 12.7 | 0.356 | 0.96 | <0.001 |
| K^+^ | 0.182 | 0.205 | 0.023 | 12.1 | 0.193 | 0.220 | 0.033 | 14.9 | 19.9 | 0.047 | 24.1 | 0.043 | 20.9 | 0.068 | 0.75 | 0.073 |
| Mg^2+^ | 0.142 | 0.174 | 0.032 | 20.5 | 0.133 | 0.177 | 0.028 | 15.7 | 17.9 | 0.015 | 9.5 | 0.018 | 11.5 | 0.053 | 0.91 | <0.001 |
| Na^+^ | 0.368 | 0.457 | 0.090 | 21.7 | 0.369 | 0.447 | 0.100 | 22.3 | 24.4 | 0.071 | 17.2 | 0.056 | 13.7 | 0.163 | 0.78 | <0.001 |
|  |  |  |  |  |  |  |  |  |  |  |  |  |  |  |  |  |

(e) Penn State University, 2005-2019, warm-season deposition

| Ion | Mean of NADP | Mean  of CAPMoN | Diff. of mean | Relative diff. of mean | Median of NADP | Median of CAPMoN | Diff. of median | Relative diff. of median | ARB | SD | RSD | MMAD | CoV | P90 | Pearson *r* | *p*-value |
| --- | --- | --- | --- | --- | --- | --- | --- | --- | --- | --- | --- | --- | --- | --- | --- | --- |
|  | kg ha^-1^ | kg ha^-1^ | kg ha^-1^ | % | kg ha^-1^ | kg ha^-1^ | kg ha^-1^ | % | % | kg ha^-1^ | % | kg ha^-1^ | % | kg ha^-1^ |  |  |
| SO_4_^2-^ | 6.076 | 6.358 | 0.282 | 4.5 | 4.639 | 4.668 | 0.245 | 5.2 | 5.3 | 0.237 | 3.8 | 0.115 | 1.9 | 0.556 | 1.00 | <0.001 |
| NO_3_^-^ | 4.126 | 4.589 | 0.463 | 10.6 | 3.876 | 4.303 | 0.427 | 9.9 | 10.4 | 0.185 | 4.2 | 0.136 | 3.1 | 0.667 | 0.99 | <0.001 |
| NH_4_^+^ | 1.398 | 1.594 | 0.196 | 13.1 | 1.379 | 1.544 | 0.178 | 11.5 | 12.2 | 0.123 | 8.3 | 0.127 | 8.5 | 0.361 | 0.96 | <0.001 |
| H^+^ | 0.101 | 0.108 | 0.007 | 7.0 | 0.061 | 0.080 | 0.011 | 13.5 | 18.2 | 0.012 | 11.8 | 0.015 | 14.1 | 0.019 | 0.99 | 0.039 |
| Ca^2+^ | 0.481 | 0.512 | 0.031 | 6.2 | 0.472 | 0.504 | 0.028 | 5.5 | 5.7 | 0.030 | 6.0 | 0.036 | 7.3 | 0.067 | 0.96 | 0.001 |
| Cl^-^ | 0.317 | 0.373 | 0.056 | 16.2 | 0.283 | 0.344 | 0.051 | 14.7 | 16.1 | 0.027 | 7.8 | 0.025 | 8.1 | 0.091 | 0.98 | <0.001 |
| K^+^ | 0.112 | 0.115 | 0.003 | 2.4 | 0.084 | 0.118 | 0.013 | 10.7 | 21.4 | 0.038 | 33.7 | 0.031 | 30.3 | 0.034 | 0.77 | 0.790 |
| Mg^2+^ | 0.077 | 0.088 | 0.011 | 13.7 | 0.082 | 0.094 | 0.011 | 11.4 | 12.1 | 0.008 | 9.2 | 0.007 | 8.1 | 0.022 | 0.92 | <0.001 |
| Na^+^ | 0.122 | 0.139 | 0.017 | 12.7 | 0.112 | 0.130 | 0.012 | 9.0 | 9.7 | 0.012 | 9.4 | 0.012 | 9.5 | 0.031 | 0.97 | <0.001 |

(f) Penn State University, 2005-2019, cold-season deposition

| Ion | Mean of NADP | Mean  of CAPMoN | Diff. of mean | Relative diff. of mean | Median of NADP | Median of CAPMoN | Diff. of median | Relative diff. of median | ARB | SD | RSD | MMAD | CoV | P90 | Pearson *r* | *p*-value |
| --- | --- | --- | --- | --- | --- | --- | --- | --- | --- | --- | --- | --- | --- | --- | --- | --- |
|  | kg ha^-1^ | kg ha^-1^ | kg ha^-1^ | % | kg ha^-1^ | kg ha^-1^ | kg ha^-1^ | % | % | kg ha^-1^ | % | kg ha^-1^ | % | kg ha^-1^ |  |  |
| SO_4_^2-^ | 3.692 | 4.276 | 0.584 | 14.7 | 3.189 | 3.358 | 0.444 | 13.2 | 13.5 | 0.417 | 10.5 | 0.484 | 12.2 | 1.152 | 1.00 | <0.001 |
| NO_3_^-^ | 4.192 | 5.316 | 1.124 | 23.6 | 3.595 | 4.501 | 0.927 | 20.6 | 22.9 | 0.714 | 15.0 | 0.611 | 12.9 | 2.149 | 0.98 | <0.001 |
| NH_4_^+^ | 1.028 | 1.262 | 0.233 | 20.4 | 0.961 | 1.179 | 0.212 | 18.0 | 19.8 | 0.127 | 11.1 | 0.123 | 10.8 | 0.432 | 0.94 | <0.001 |
| H^+^ | 0.072 | 0.086 | 0.014 | 17.1 | 0.047 | 0.070 | 0.012 | 17.4 | 20.8 | 0.011 | 13.8 | 0.008 | 9.5 | 0.026 | 0.98 | <0.001 |
| Ca^2+^ | 0.426 | 0.555 | 0.129 | 26.2 | 0.384 | 0.542 | 0.133 | 24.4 | 28.6 | 0.058 | 11.8 | 0.068 | 14.6 | 0.201 | 0.93 | <0.001 |
| Cl^-^ | 0.497 | 0.653 | 0.155 | 27.0 | 0.529 | 0.674 | 0.142 | 21.0 | 23.5 | 0.089 | 15.4 | 0.091 | 15.1 | 0.275 | 0.92 | <0.001 |
| K^+^ | 0.069 | 0.090 | 0.021 | 26.0 | 0.059 | 0.077 | 0.017 | 21.8 | 28.3 | 0.027 | 33.6 | 0.013 | 19.2 | 0.054 | 0.70 | 0.010 |
| Mg^2+^ | 0.065 | 0.086 | 0.021 | 28.0 | 0.062 | 0.080 | 0.021 | 26.4 | 30.0 | 0.011 | 14.3 | 0.011 | 15.2 | 0.036 | 0.91 | <0.001 |
| Na^+^ | 0.245 | 0.318 | 0.073 | 25.9 | 0.246 | 0.293 | 0.077 | 26.1 | 30.5 | 0.069 | 24.4 | 0.045 | 16.8 | 0.144 | 0.75 | 0.001 |

(g) Frelighsburg, 2002-2011, annual deposition

| Ion | Mean of NADP | Mean  of CAPMoN | Diff. of mean | Relative diff. of mean | Median of NADP | Median of CAPMoN | Diff. of median | Relative diff. of median | ARB | SD | RSD | MMAD | CoV | P90 | Pearson *r* | *p*-value |
| --- | --- | --- | --- | --- | --- | --- | --- | --- | --- | --- | --- | --- | --- | --- | --- | --- |
|  | kg ha^-1^ | kg ha^-1^ | kg ha^-1^ | % | kg ha^-1^ | kg ha^-1^ | kg ha^-1^ | % | % | kg ha^-1^ | % | kg ha^-1^ | % | kg ha^-1^ |  |  |
| SO_4_^2-^ | 9.072 | 10.205 | 1.133 | 11.8 | 8.505 | 10.001 | 1.115 | 11.1 | 12.1 | 0.611 | 6.3 | 0.724 | 7.5 | 1.861 | 0.97 | 0.000 |
| NO_3_^-^ | 8.270 | 10.246 | 1.975 | 21.3 | 7.871 | 9.935 | 1.809 | 18.2 | 20.3 | 0.757 | 8.2 | 0.733 | 7.9 | 3.032 | 0.96 | 0.000 |
| NH_4_^+^ | 2.496 | 3.212 | 0.716 | 25.1 | 2.592 | 3.373 | 0.777 | 23.0 | 26.0 | 0.254 | 8.9 | 0.298 | 10.4 | 0.991 | 0.95 | 0.000 |
| H^+^ | 0.147 | 0.176 | 0.028 | 17.6 | 0.143 | 0.169 | 0.024 | 14.2 | 15.4 | 0.020 | 12.1 | 0.014 | 8.7 | 0.061 | 0.93 | 0.001 |
| Ca^2+^ | 0.898 | 1.137 | 0.239 | 23.5 | 0.888 | 1.122 | 0.236 | 21.0 | 23.5 | 0.224 | 22.1 | 0.272 | 27.0 | 0.524 | 0.78 | 0.008 |
| Cl^-^ | 0.516 | 0.627 | 0.111 | 19.4 | 0.483 | 0.587 | 0.106 | 18.1 | 27.5 | 0.134 | 23.5 | 0.086 | 16.1 | 0.265 | 0.71 | 0.028 |
| K^+^ | 0.144 | 0.175 | 0.031 | 19.7 | 0.131 | 0.169 | 0.028 | 16.5 | 18.6 | 0.022 | 13.9 | 0.030 | 19.7 | 0.053 | 0.95 | 0.002 |
| Mg^2+^ | 0.113 | 0.148 | 0.035 | 26.9 | 0.113 | 0.143 | 0.028 | 19.4 | 21.6 | 0.020 | 15.5 | 0.019 | 14.8 | 0.060 | 0.86 | 0.000 |
| Na^+^ | 0.249 | 0.303 | 0.054 | 19.6 | 0.234 | 0.301 | 0.059 | 19.6 | 31.3 | 0.077 | 28.0 | 0.067 | 24.8 | 0.133 | 0.73 | 0.054 |

(h) Frelighsburg, 2002-2011, warm-season deposition

| Ion | Mean of NADP | Mean  of CAPMoN | Diff. of mean | Relative diff. of mean | Median of NADP | Median of CAPMoN | Diff. of median | Relative diff. of median | ARB | SD | RSD | MMAD | CoV | P90 | Pearson *r* | *p*-value |
| --- | --- | --- | --- | --- | --- | --- | --- | --- | --- | --- | --- | --- | --- | --- | --- | --- |
|  | kg ha^-1^ | kg ha^-1^ | kg ha^-1^ | % | kg ha^-1^ | kg ha^-1^ | kg ha^-1^ | % | % | kg ha^-1^ | % | kg ha^-1^ | % | kg ha^-1^ |  |  |
| SO_4_^2-^ | 6.374 | 6.938 | 0.564 | 8.5 | 5.499 | 6.440 | 0.484 | 7.5 | 8.1 | 0.325 | 4.9 | 0.381 | 5.7 | 1.032 | 0.99 | 0.000 |
| NO_3_^-^ | 4.439 | 5.127 | 0.688 | 14.4 | 4.190 | 4.840 | 0.622 | 12.9 | 13.8 | 0.236 | 4.9 | 0.231 | 4.8 | 0.993 | 0.99 | 0.000 |
| NH_4_^+^ | 1.714 | 2.126 | 0.412 | 21.5 | 1.857 | 2.216 | 0.372 | 16.8 | 18.3 | 0.161 | 8.4 | 0.114 | 5.9 | 0.573 | 0.95 | 0.000 |
| H^+^ | 0.092 | 0.104 | 0.012 | 12.4 | 0.074 | 0.089 | 0.013 | 14.3 | 15.7 | 0.014 | 14.4 | 0.010 | 9.8 | 0.022 | 0.95 | 0.023 |
| Ca^2+^ | 0.524 | 0.629 | 0.106 | 18.3 | 0.540 | 0.677 | 0.121 | 17.8 | 19.8 | 0.065 | 11.2 | 0.071 | 11.6 | 0.171 | 0.95 | 0.001 |
| Cl^-^ | 0.235 | 0.270 | 0.035 | 13.9 | 0.224 | 0.257 | 0.032 | 12.3 | 13.1 | 0.027 | 10.7 | 0.028 | 11.5 | 0.067 | 0.94 | 0.003 |
| K^+^ | 0.112 | 0.125 | 0.013 | 10.6 | 0.095 | 0.115 | 0.011 | 9.3 | 19.4 | 0.028 | 23.4 | 0.024 | 22.9 | 0.049 | 0.87 | 0.185 |
| Mg^2+^ | 0.073 | 0.092 | 0.019 | 23.7 | 0.079 | 0.096 | 0.020 | 20.4 | 22.5 | 0.006 | 7.5 | 0.007 | 8.5 | 0.025 | 0.97 | 0.000 |
| Na^+^ | 0.089 | 0.105 | 0.016 | 16.0 | 0.095 | 0.101 | 0.016 | 15.8 | 16.3 | 0.010 | 10.2 | 0.013 | 13.6 | 0.026 | 0.92 | 0.001 |

(i) Frelighsburg, 2002-2011, cold-season deposition

| Ion | Mean of NADP | Mean  of CAPMoN | Diff. of mean | Relative diff. of mean | Median of NADP | Median of CAPMoN | Diff. of median | Relative diff. of median | ARB | SD | RSD | MMAD | CoV | P90 | Pearson *r* | *p*-value |
| --- | --- | --- | --- | --- | --- | --- | --- | --- | --- | --- | --- | --- | --- | --- | --- | --- |
|  | kg ha^-1^ | kg ha^-1^ | kg ha^-1^ | % | kg ha^-1^ | kg ha^-1^ | kg ha^-1^ | % | % | kg ha^-1^ | % | kg ha^-1^ | % | kg ha^-1^ |  |  |
| SO_4_^2-^ | 2.698 | 3.267 | 0.568 | 19.1 | 2.918 | 3.552 | 0.573 | 16.1 | 19.4 | 0.523 | 17.5 | 0.420 | 14.1 | 1.157 | 0.90 | 0.007 |
| NO_3_^-^ | 3.832 | 5.119 | 1.287 | 28.8 | 3.751 | 5.372 | 1.257 | 23.4 | 27.6 | 0.789 | 17.6 | 0.676 | 15.1 | 2.035 | 0.92 | 0.001 |
| NH_4_^+^ | 0.782 | 1.086 | 0.304 | 32.6 | 0.865 | 1.246 | 0.319 | 25.6 | 30.3 | 0.210 | 22.5 | 0.215 | 23.1 | 0.554 | 0.89 | 0.001 |
| H^+^ | 0.055 | 0.071 | 0.016 | 25.6 | 0.059 | 0.080 | 0.013 | 16.4 | 18.9 | 0.010 | 16.2 | 0.006 | 9.7 | 0.022 | 0.94 | 0.001 |
| Ca^2+^ | 0.374 | 0.508 | 0.134 | 30.3 | 0.370 | 0.538 | 0.161 | 30.0 | 51.2 | 0.194 | 44.0 | 0.173 | 38.1 | 0.338 | 0.64 | 0.058 |
| Cl^-^ | 0.281 | 0.356 | 0.076 | 23.7 | 0.280 | 0.373 | 0.095 | 25.5 | 31.1 | 0.124 | 38.9 | 0.070 | 21.3 | 0.192 | 0.68 | 0.086 |
| K^+^ | 0.032 | 0.050 | 0.019 | 45.9 | 0.033 | 0.049 | 0.018 | 37.6 | 45.1 | 0.012 | 28.2 | 0.015 | 37.8 | 0.032 | 0.91 | 0.001 |
| Mg^2+^ | 0.040 | 0.056 | 0.016 | 32.5 | 0.039 | 0.059 | 0.015 | 25.8 | 38.8 | 0.018 | 37.0 | 0.018 | 35.8 | 0.035 | 0.74 | 0.021 |
| Na^+^ | 0.160 | 0.198 | 0.039 | 21.6 | 0.155 | 0.222 | 0.048 | 21.6 | 37.7 | 0.076 | 42.2 | 0.060 | 31.7 | 0.118 | 0.65 | 0.140 |
|  |  |  |  |  |  |  |  |  |  |  |  |  |  |  |  |  |

(j) Frelighsburg, 2012-2019, annual deposition

| Ion | Mean of NADP | Mean  of CAPMoN | Diff. of mean | Relative diff. of mean | Median of NADP | Median of CAPMoN | Diff. of median | Relative diff. of median | ARB | SD | RSD | MMAD | CoV | P90 | Pearson *r* | *p*-value |
| --- | --- | --- | --- | --- | --- | --- | --- | --- | --- | --- | --- | --- | --- | --- | --- | --- |
|  | kg ha^-1^ | kg ha^-1^ | kg ha^-1^ | % | kg ha^-1^ | kg ha^-1^ | kg ha^-1^ | % | % | kg ha^-1^ | % | kg ha^-1^ | % | kg ha^-1^ |  |  |
| SO_4_^2-^ | 5.477 | 5.045 | -0.432 | -8.2 | 6.143 | 4.935 | -0.149 | -3.0 | 6.0 | 0.879 | 16.7 | 0.923 | 17.5 | 0.313 | 0.91 | 0.207 |
| NO_3_^-^ | 8.864 | 7.890 | -0.974 | -11.6 | 9.399 | 7.891 | -0.354 | -4.5 | 5.1 | 1.888 | 22.5 | 1.389 | 16.6 | 0.248 | 0.65 | 0.188 |
| NH_4_^+^ | 3.629 | 3.328 | -0.301 | -8.7 | 3.651 | 3.507 | -0.094 | -2.7 | 6.1 | 0.557 | 16.0 | 0.515 | 14.8 | 0.097 | 0.82 | 0.170 |
| H^+^ | 0.063 | 0.079 | 0.016 | 22.4 | 0.066 | 0.081 | 0.024 | 29.8 | 44.3 | 0.027 | 38.8 | 0.025 | 34.7 | 0.035 | 0.38 | 0.146 |
| Ca^2+^ | 1.271 | 1.091 | -0.180 | -15.3 | 1.153 | 1.022 | -0.157 | -15.4 | 19.3 | 0.289 | 24.4 | 0.217 | 20.0 | 0.062 | 0.57 | 0.121 |
| Cl^-^ | 0.774 | 0.623 | -0.151 | -21.6 | 0.726 | 0.643 | -0.062 | -9.6 | 9.0 | 0.256 | 36.6 | 0.163 | 23.7 | -0.021 | 0.33 | 0.140 |
| K^+^ | 0.244 | 0.198 | -0.046 | -20.9 | 0.219 | 0.179 | -0.025 | -13.9 | 16.1 | 0.089 | 40.0 | 0.055 | 27.6 | 0.017 | 0.77 | 0.184 |
| Mg^2+^ | 0.168 | 0.156 | -0.012 | -7.2 | 0.165 | 0.149 | -0.008 | -5.3 | 13.1 | 0.026 | 16.3 | 0.027 | 17.4 | 0.011 | 0.75 | 0.251 |
| Na^+^ | 0.435 | 0.339 | -0.095 | -24.7 | 0.399 | 0.338 | -0.037 | -11.0 | 10.1 | 0.164 | 42.4 | 0.110 | 29.7 | -0.003 | 0.27 | 0.144 |

(k) Frelighsburg, 2012-2019, warm-season deposition

| Ion | Mean of NADP | Mean  of CAPMoN | Diff. of mean | Relative diff. of mean | Median of NADP | Median of CAPMoN | Diff. of median | Relative diff. of median | ARB | SD | RSD | MMAD | CoV | P90 | Pearson *r* | *p*-value |
| --- | --- | --- | --- | --- | --- | --- | --- | --- | --- | --- | --- | --- | --- | --- | --- | --- |
|  | kg ha^-1^ | kg ha^-1^ | kg ha^-1^ | % | kg ha^-1^ | kg ha^-1^ | kg ha^-1^ | % | % | kg ha^-1^ | % | kg ha^-1^ | % | kg ha^-1^ |  |  |
| SO_4_^2-^ | 2.698 | 2.783 | 0.084 | 3.1 | 2.289 | 2.337 | 0.045 | 1.9 | 2.4 | 0.153 | 5.6 | 0.130 | 4.7 | 0.283 | 1.00 | 0.164 |
| NO_3_^-^ | 3.263 | 3.330 | 0.067 | 2.0 | 3.194 | 3.127 | 0.092 | 2.9 | 5.0 | 0.158 | 4.8 | 0.190 | 5.8 | 0.235 | 1.00 | 0.270 |
| NH_4_^+^ | 1.934 | 1.952 | 0.018 | 0.9 | 1.874 | 1.909 | 0.040 | 2.1 | 4.5 | 0.102 | 5.2 | 0.107 | 5.5 | 0.115 | 0.99 | 0.630 |
| H^+^ | 0.017 | 0.034 | 0.017 | 65.4 | 0.020 | 0.037 | 0.017 | 46.8 | 60.8 | 0.006 | 24.4 | 0.006 | 23.3 | 0.023 | 0.97 | 0.000 |
| Ca^2+^ | 0.520 | 0.535 | 0.015 | 2.9 | 0.486 | 0.467 | 0.000 | 0.0 | 6.3 | 0.082 | 15.5 | 0.058 | 12.3 | 0.079 | 0.97 | 0.617 |
| Cl^-^ | 0.213 | 0.224 | 0.011 | 5.2 | 0.226 | 0.240 | 0.010 | 4.3 | 4.4 | 0.011 | 4.9 | 0.015 | 6.3 | 0.026 | 0.99 | 0.021 |
| K^+^ | 0.177 | 0.134 | -0.042 | -27.3 | 0.139 | 0.114 | -0.010 | -8.9 | 18.3 | 0.089 | 57.2 | 0.053 | 41.6 | 0.011 | 0.84 | 0.219 |
| Mg^2+^ | 0.082 | 0.086 | 0.004 | 4.7 | 0.077 | 0.079 | 0.004 | 5.3 | 8.0 | 0.010 | 12.2 | 0.008 | 10.7 | 0.013 | 0.97 | 0.317 |
| Na^+^ | 0.099 | 0.106 | 0.007 | 7.0 | 0.089 | 0.110 | 0.004 | 3.6 | 7.7 | 0.012 | 12.2 | 0.009 | 8.9 | 0.023 | 0.96 | 0.149 |

(l) Frelighsburg, 2012-2019, cold-season deposition

| Ion | Mean of NADP | Mean  of CAPMoN | Diff. of mean | Relative diff. of mean | Median of NADP | Median of CAPMoN | Diff. of median | Relative diff. of median | ARB | SD | RSD | MMAD | CoV | P90 | Pearson *r* | *p*-value |
| --- | --- | --- | --- | --- | --- | --- | --- | --- | --- | --- | --- | --- | --- | --- | --- | --- |
|  | kg ha^-1^ | kg ha^-1^ | kg ha^-1^ | % | kg ha^-1^ | kg ha^-1^ | kg ha^-1^ | % | % | kg ha^-1^ | % | kg ha^-1^ | % | kg ha^-1^ |  |  |
| SO_4_^2-^ | 2.779 | 2.262 | -0.517 | -20.5 | 2.505 | 2.265 | -0.146 | -6.4 | 6.1 | 0.782 | 31.0 | 0.796 | 31.6 | 0.040 | 0.75 | 0.104 |
| NO_3_^-^ | 5.601 | 4.560 | -1.040 | -20.5 | 4.868 | 4.457 | -0.391 | -8.8 | 8.4 | 1.787 | 35.2 | 1.428 | 28.1 | 0.040 | 0.44 | 0.144 |
| NH_4_^+^ | 1.695 | 1.376 | -0.319 | -20.8 | 1.447 | 1.423 | -0.120 | -8.4 | 8.4 | 0.493 | 32.1 | 0.424 | 27.6 | -0.007 | 0.74 | 0.110 |
| H^+^ | 0.045 | 0.044 | -0.001 | -2.4 | 0.043 | 0.044 | 0.006 | 13.9 | 24.1 | 0.025 | 55.6 | 0.017 | 38.0 | 0.014 | 0.11 | 0.907 |
| Ca^2+^ | 0.751 | 0.556 | -0.195 | -29.9 | 0.715 | 0.508 | -0.125 | -24.5 | 20.4 | 0.260 | 39.8 | 0.222 | 36.3 | -0.018 | 0.70 | 0.071 |
| Cl^-^ | 0.561 | 0.399 | -0.162 | -33.7 | 0.503 | 0.397 | -0.070 | -17.5 | 15.5 | 0.253 | 52.7 | 0.157 | 34.8 | -0.031 | 0.30 | 0.113 |
| K^+^ | 0.068 | 0.064 | -0.004 | -5.6 | 0.058 | 0.064 | 0.003 | 4.6 | 16.5 | 0.022 | 33.1 | 0.017 | 27.2 | 0.011 | 0.84 | 0.649 |
| Mg^2+^ | 0.086 | 0.070 | -0.016 | -20.0 | 0.080 | 0.065 | -0.007 | -11.4 | 10.8 | 0.022 | 28.5 | 0.020 | 27.3 | 0.000 | 0.71 | 0.088 |
| Na^+^ | 0.336 | 0.234 | -0.103 | -36.0 | 0.287 | 0.228 | -0.040 | -17.7 | 15.6 | 0.162 | 56.8 | 0.103 | 39.9 | -0.024 | 0.28 | 0.116 |

Table S.5 Median weekly concentrations and relative differences of daily-vs-daily, weekly-vs-weekly, and daily-vs-weekly measurements at Egbert, Ontario, Canada for 1999-2001. Two daily samples (EG1 and EG2) and two weekly samples (EW1 and EW2) were deployed at the measurement site. EG1&2-minus-EW1 is calculated as the mean of EG1-minus-EW1 and EG2-minus-EW1, and it is similar for EG1&2-minus-EW2; Daily-minus-Weekly is calculated as the mean of EG1-minus-EW1, EG2-minus-EW1, EG1-minus-EW2, and EG2-minus-EW2. The calculation of the relative difference is based Eq. 5 in the text. Data obtained from NADP, Wisconsin State Laboratory of Hygiene at https://nadp.slh.wisc.edu/networks/national-trends-network and Environment and Climate Change Canada at https://www.canada.ca/en/environment-climate-change/services/air-pollution/monitoring-networks-data/canadian-air-precipitation.html, last accessed August 2023.

|  | Median concentration and median weekly precipitation depth | | | | Relative difference | | | | | | | | |
| --- | --- | --- | --- | --- | --- | --- | --- | --- | --- | --- | --- | --- | --- |
| Ion | EG1 | EG2 | EW1 | EW2 | EG2 minus  EG1 | EW2 minus EW1 | EG1 minus  EW1 | EG1  minus  EW2 | EG2  minus  EW1 | EG2  minus  EW2 | EG1&2  minus  EW1 | EG1&2  minus  EW2 | Daily  minus  Weekly |
|  | μeq L^-1^ | μeq L^-1^ | μeq L^-1^ | μeq L^-1^ | % | % | % | % | % | % | % | % | % |
| SO_4_^2-^ | 36.7 | 35.3 | 38.3 | 37.9 | -0.5 | -0.5 | -3.4 | -2.7 | -3.4 | -3.2 | -3.4 | -2.9 | -3.2 |
| NO_3_^-^ | 35.2 | 35.3 | 36.0 | 35.9 | -0.3 | -0.2 | -1.4 | -1.2 | -1.5 | -1.2 | -1.5 | -1.2 | -1.3 |
| NH_4_^+^ | 22.9 | 23.4 | 23.6 | 24.2 | -1.0 | -0.5 | -2.9 | -0.9 | -2.6 | -2.1 | -2.7 | -1.5 | -2.1 |
| H^+^ | 29.0 | 29.0 | 25.5 | 27.0 | 0.0 | 0.0 | 7.3 | 3.6 | 7.3 | 3.6 | 7.3 | 3.6 | 5.5 |
| Ca^2+^ | 11.7 | 11.3 | 11.4 | 11.0 | -1.3 | -0.9 | -1.7 | -0.9 | -2.6 | -2.2 | -2.2 | -1.6 | -1.9 |
| Mg^2+^ | 3.0 | 2.7 | 2.8 | 2.9 | 0.0 | 0.0 | -5.6 | -2.8 | -6.0 | -5.9 | -5.8 | -4.3 | -5.1 |
| K^+^ | 0.7 | 0.7 | 0.7 | 0.7 | 0.0 | -3.5 | -7.1 | -3.6 | -7.1 | -7.1 | -7.1 | -5.4 | -6.3 |
| Na^+^ | 2.3 | 2.2 | 2.1 | 2.0 | 0.0 | 0.0 | -2.0 | -0.0 | -2.1 | -2.1 | -2.0 | -1.0 | -1.5 |
| Cl^-^ | 4.0 | 4.0 | 4.0 | 4.0 | -0.7 | 0.0 | -1.1 | -0.7 | -1.4 | -1.8 | -1.2 | -1.2 | -1.2 |


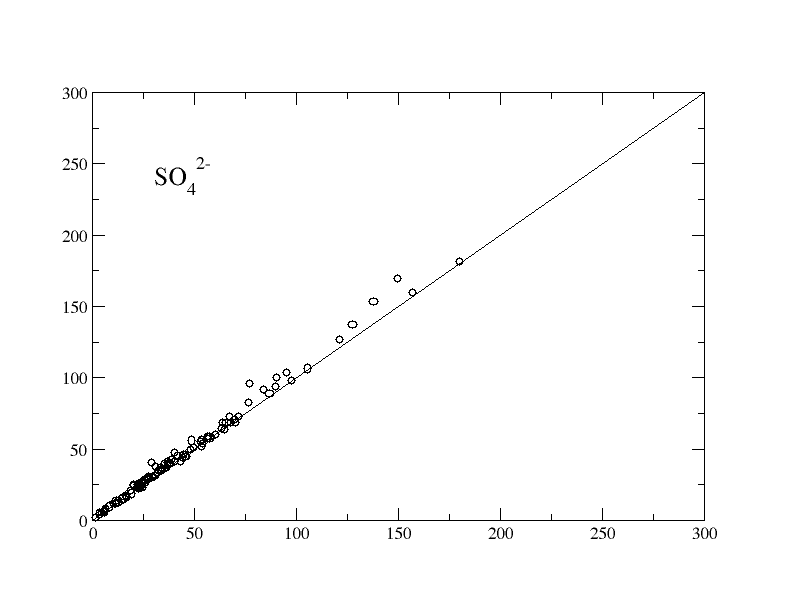

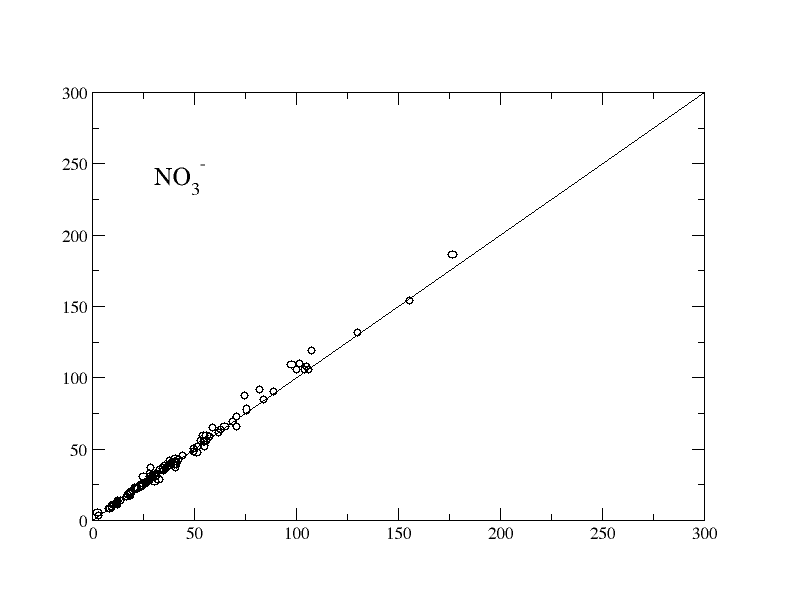

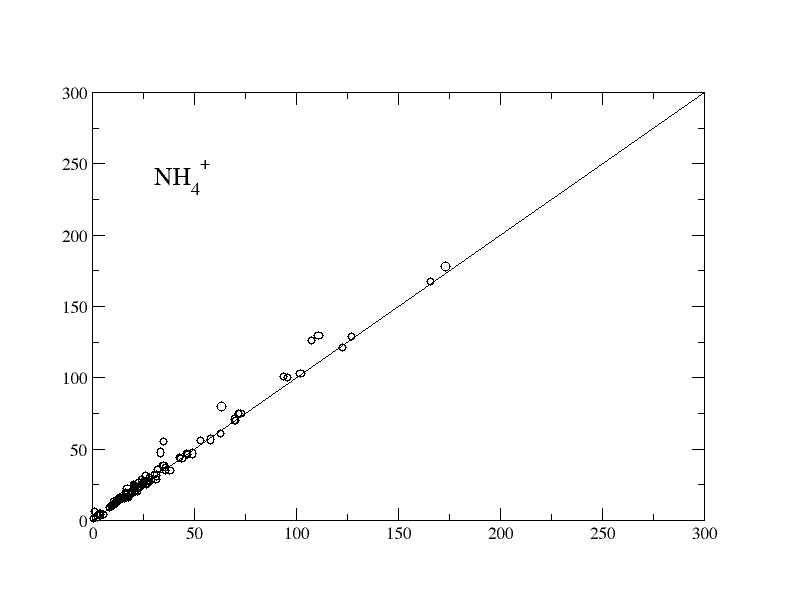

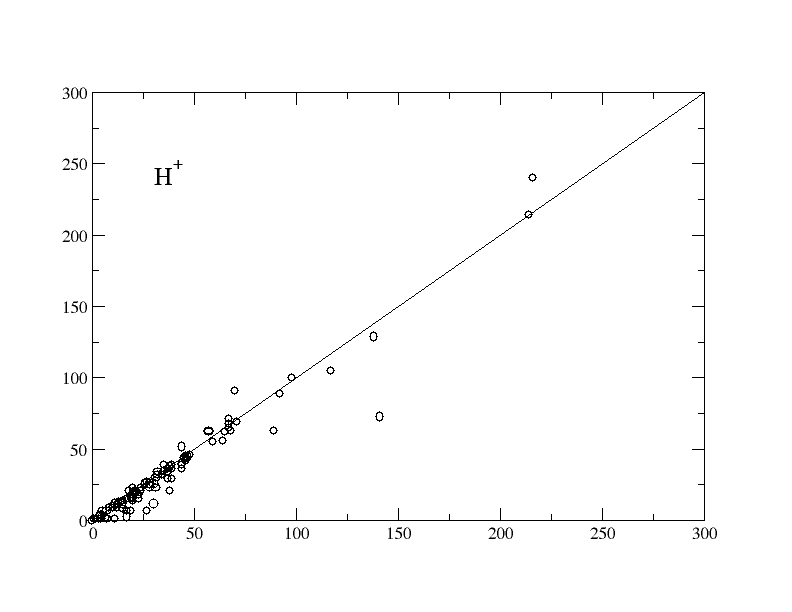

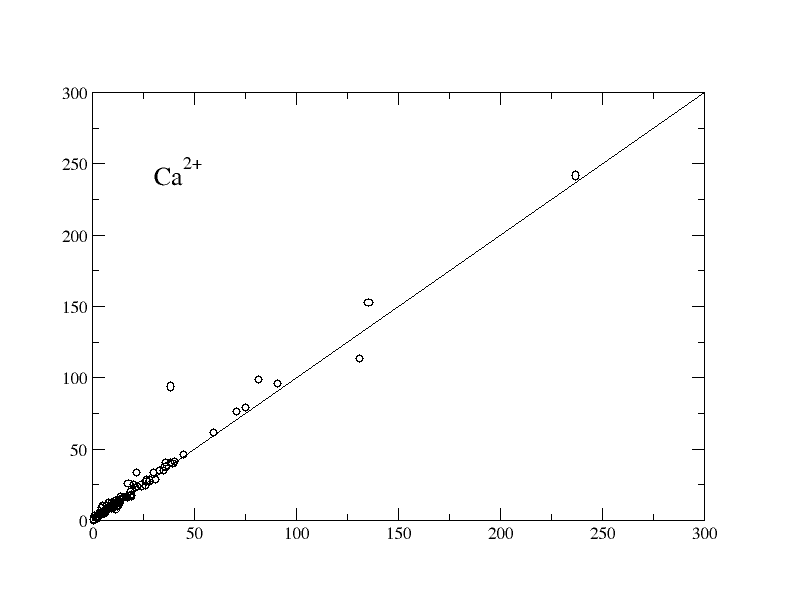

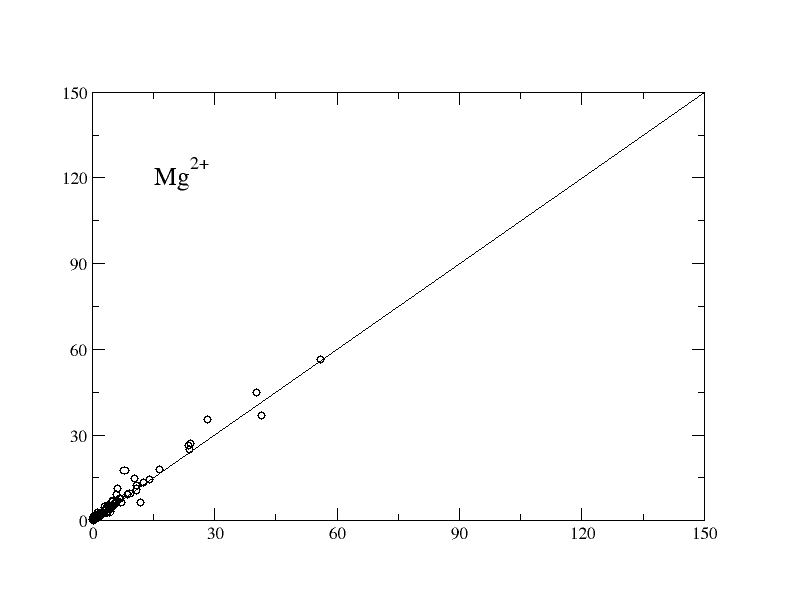

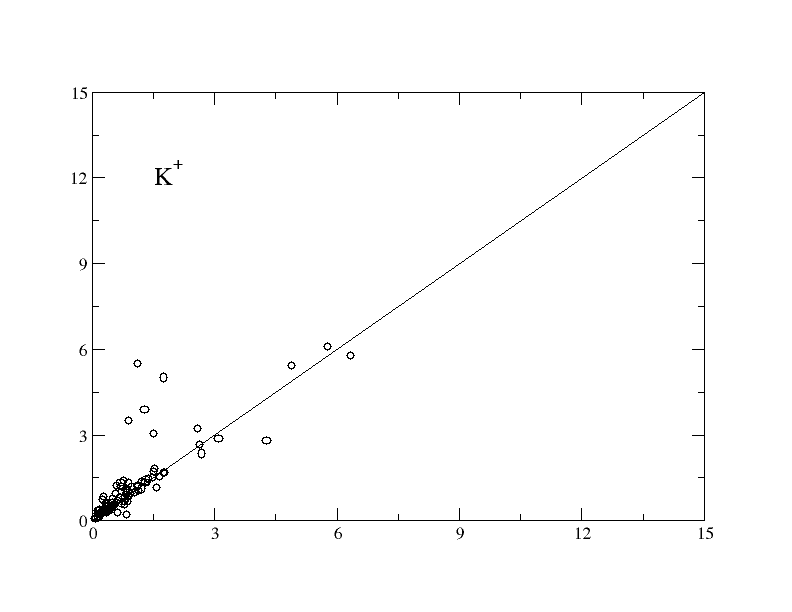

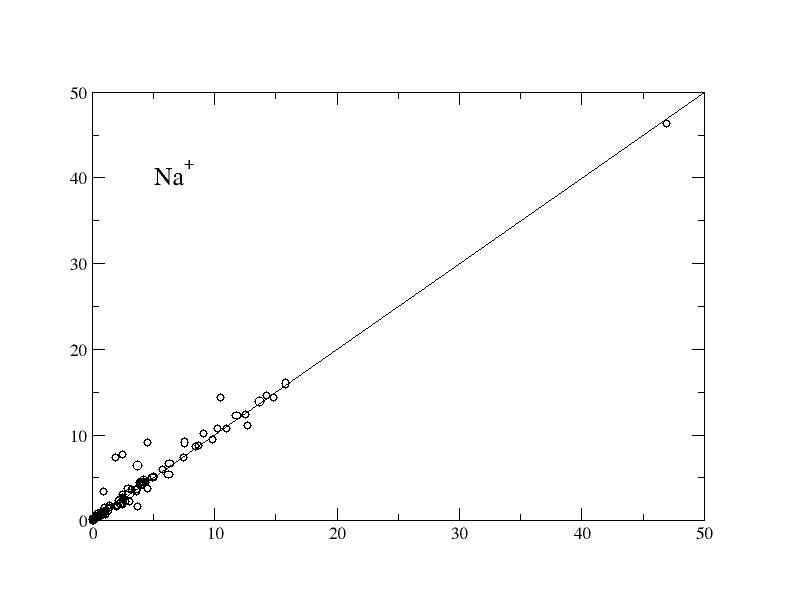

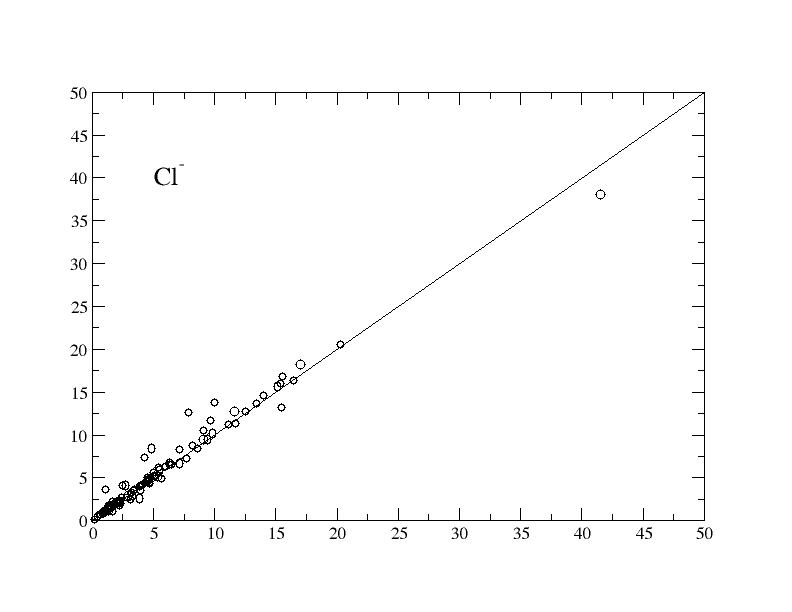


Weekly concentration from weekly samples – EW1 (μeq L^-1^)

Weekly concentration from daily samples – EG1 (μeq L^-1^)

Fig. S.1 Weekly concentrations (μeq L^-1^) from daily and weekly samples at Egbert, Canada during 1999-2001. EG1 and EW1 are daily and weekly samples, respectively.


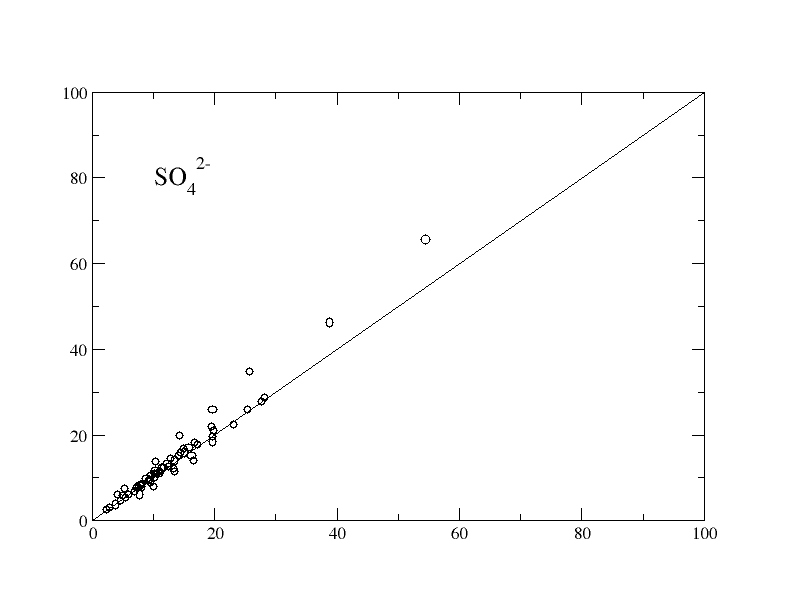

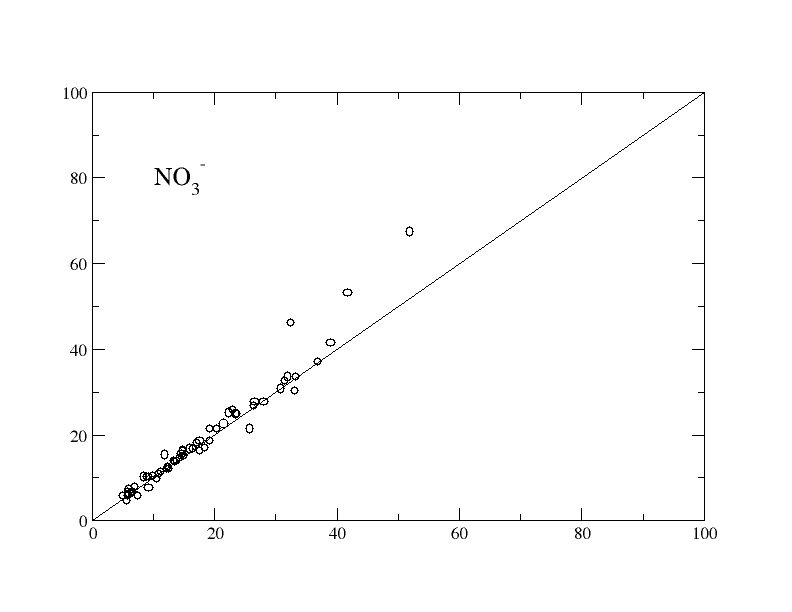

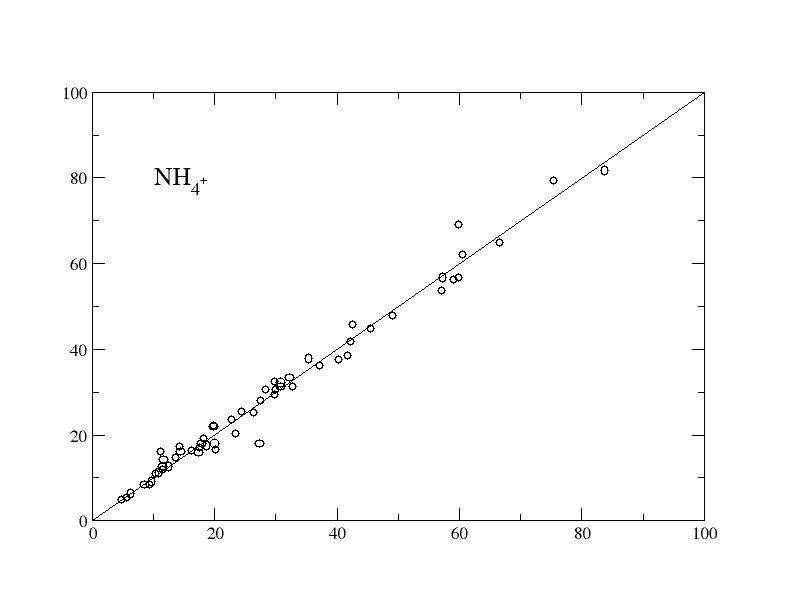

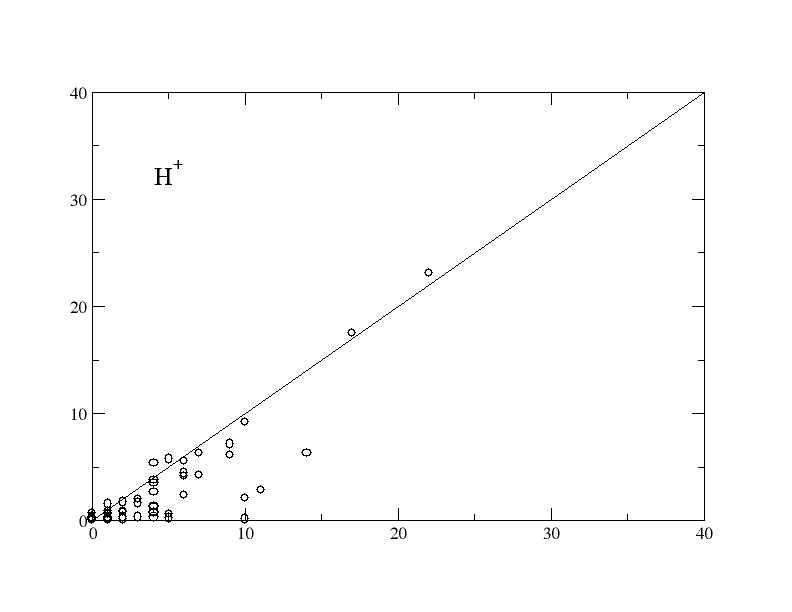

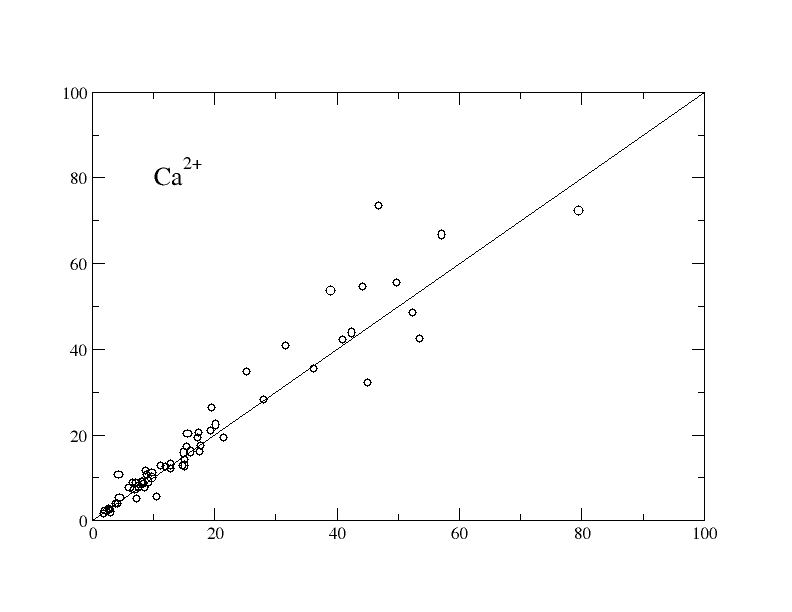

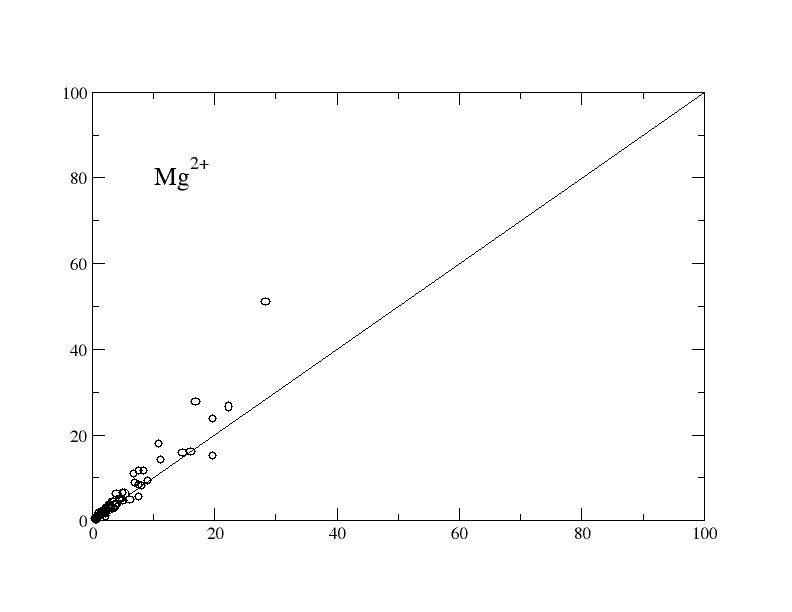

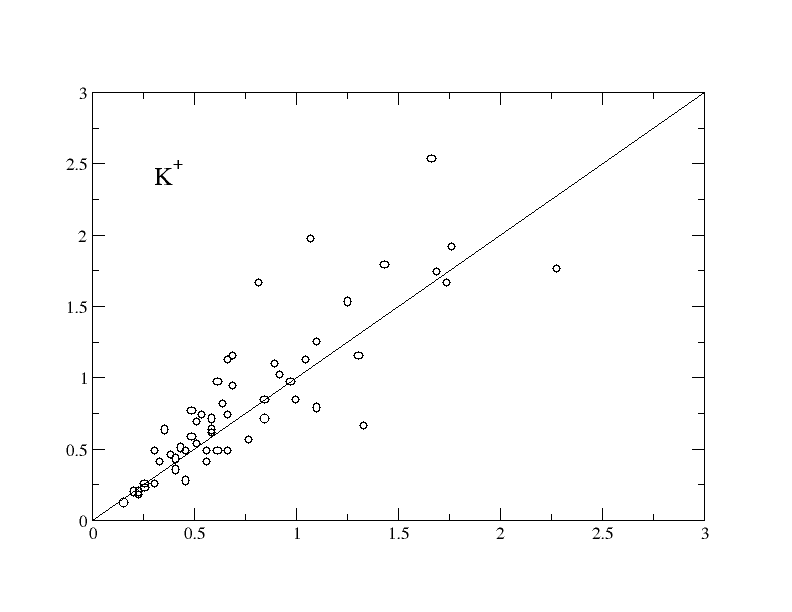

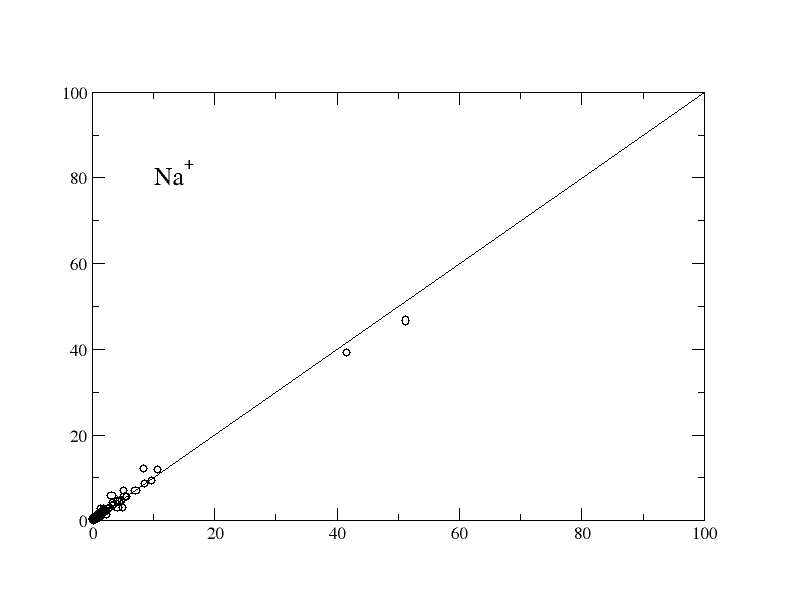

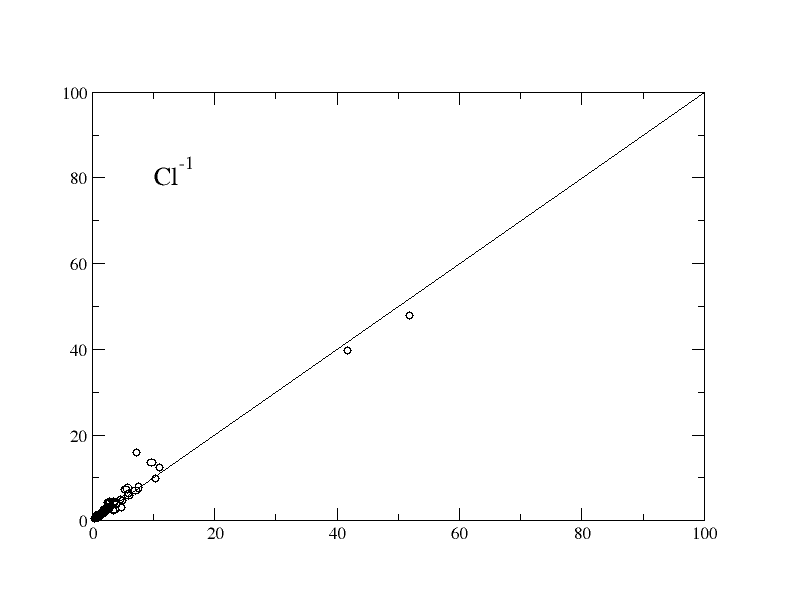


Weekly concentration from weekly samples – EGW (μeq L^-1^)

Weekly concentration from daily samples – EGD (μeq L^-1^)

Fig. S.2 Weekly concentrations (μeq L^-1^) from daily and weekly samples at Egbert, Canada during 2016-2017. EGD and EGW are daily and weekly samples collected with D400 samplers, respectively.
